# MERWACS: Development and external validation of a non-invasive machine learning tool for identifying subjects to be screened for CKD

**DOI:** 10.1371/journal.pdig.0001486

**Published:** 2026-07-09

**Authors:** Daniel Yoo, Vy Kim Nguyen, Umberto Maggiore, Olivier Jolliet

**Affiliations:** 1 Section for Quantitative Sustainability Assessment, Department of Environmental and Resource Engineering, Technical University of Denmark, Kongens Lyngby, Denmark; 2 Department of Environmental Health Sciences, School of Public Health, University of Michigan‌‌, Ann Arbor, Michigan, United States of America; 3 Department of Medicine & Surgery, University of Parma, Parma, Italy; Mayo Clinic Rochester: Mayo Clinic Minnesota, UNITED STATES OF AMERICA

## Abstract

Early detection of chronic kidney disease (CKD) is a critical public health priority. However, a gap exists for non-invasive tools to guide screening selection in the general adult population, leaving many at-risk individuals undiagnosed. We developed and validated MERWACS (Machineborne Early Renal Warning And Control System), a machine learning model designed to identify which individuals should be prioritized for definitive laboratory testing. We used 30 years of data from the U.S. National Health and Nutrition Examination Survey (NHANES; training set n = 9,534) to train MERWACS using a final set of 12 non-invasive parameters derived from demographics, anthropometrics, and medical history. The model predicts a composite outcome of prevalent reduced kidney function, defined as a urine albumin-to-creatinine ratio ≥ 30 mg/g or an estimated glomerular filtration rate (eGFR) below the age- and sex-specific 2.5th percentile, thereby accounting for the natural eGFR decline in healthy aging. To ensure robustness, we developed parallel models for three major eGFR equations. The final XGBoost-based model was validated on an internal test set (n = 4,085) and a separate external dataset from the Korea NHANES (KNHANES; n = 6,454). MERWACS demonstrated moderate and consistent discrimination across all three eGFR equation-based models, achieving ROCAUCs ranging from 0.68 to 0.70 on internal validation and 0.71 to 0.73 on external validation. MERWACS is a robust, externally validated, non-invasive tool that identifies adults and elderly individuals with a high probability of currently having reduced kidney health, helping prioritize them for definitive CKD screening. Its moderate performance is an intentional trade-off for accessibility, as it deliberately excludes laboratory data. By providing a personalized, numerical predicted probability through an open-access online application, MERWACS can empower individuals and support clinicians in identifying at-risk patients, prompting timely conversations and crucial evaluations to improve kidney health outcomes.

## Author summary

Chronic kidney disease (CKD) is a common and serious condition, but it often has no early symptoms, meaning millions of people are unaware they are at risk. In our study, we wanted to create a simple, accessible tool to help identify adult and elderly individuals who might benefit from laboratory screening for CKD, without requiring an initial lab test. A key feature of our tool is that it accounts for the natural decline in kidney function that occurs as we age.

We used artificial intelligence to analyze health information from thousands of adults, focusing only on easily measured factors like age, weight, blood pressure, and self-reported medical history. From this, we built a tool called MERWACS that generates a personalized predicted probability of having reduced kidney function. To ensure it was reliable, we rigorously tested it on large, separate datasets of people from both the United States and South Korea.

We have made MERWACS available as a free, interactive online application. Our goal is not to replace doctors or lab tests, but to provide an early warning that helps identify which adult and elderly subjects should undergo CKD screening, ultimately prompting earlier, life-saving conversations about kidney health.

## Introduction

Chronic kidney disease (CKD) represents a growing public health concern, particularly in aging populations [1,2]. It functions as a powerful risk multiplier for cardiovascular disease (CVD), yet often progresses silently until its advanced stages [3,4]. With the recent advent of highly effective therapies that can slow CKD progression and reduce cardiovascular events, such as SGLT2 inhibitors, the imperative for identifying undiagnosed prevalent CKD in the general population has never been greater [5,6]. However, definitive diagnosis relies on laboratory tests. Under current guidelines, these tests are primarily recommended for individuals with established risk factors, such as diabetes or hypertension, rather than for the general population [1]. The logistical and financial challenges of implementing universal laboratory screening are substantial, creating a significant “screening gap” where a large portion of the at-risk population remains undiagnosed [1,7].

Current clinical guidance for screening often relies on a series of dichotomous cut-offs. For example, screening may be suggested for individuals older than 55 or for those with a formal diagnosis of ‘hypertension’ or ‘obesity’ [7]. While practical, this binary (yes/no) logic has major limitations. It fails to account for the continuous nature of risk, treating a systolic blood pressure of 141 mmHg the same as 180 mmHg and ignoring the graded risk across the full spectrum of age and body mass index. Furthermore, these guidelines typically present risk factors as a checklist, without providing a method to quantify the combined, synergistic effect of multiple concurrent factors. Consequently, a fundamental gap exists: there is no validated tool that integrates these risk factors on a continuous numerical scale to provide a single, personalized probability to help guide screening decisions.

Furthermore, the standard definition of CKD, based on a fixed estimated glomerular filtration rate (eGFR) threshold of <60 ml/min/1.73m^2^ [1], poses an additional clinical challenge as it fails to distinguish pathological renal decline from normal physiological aging [8,9]. Recent meta-analyses providing age- and sex-specific normative eGFR distributions offer a more nuanced approach [10]. Albuminuria, a key marker of kidney damage, is also a potent but underutilized predictor of adverse cardiovascular and renal outcomes [11,12]. Therefore, defining “reduced kidney health” as a deviation from these healthy peer-group norms, combined with the presence of albuminuria, represents a more precise and biologically relevant endpoint for a tool meant to identify who should be screened.

Accurately modeling this complex landscape requires an approach that can overcome the limitations of traditional linear methods. The inter-correlation of risk factors and the non-linear relationship between continuous variables and kidney health necessitate a more sophisticated method [13,14]. Machine learning, particularly non-linear algorithms, offers a powerful alternative specifically designed to learn these intricate patterns directly from the data [15,16]. Therefore, this manuscript presents a novel machine learning framework designed to help identify subjects to be screened for CKD. By providing a numerical assessment of the predicted probability, it is intended to support both physicians and patients.

In this study, we developed and validated a machine learning model—the Machineborne Early Renal Warning And Control System (MERWACS)—designed to address these critical gaps. MERWACS is a non-invasive pre-screening tool that uses only easily accessible parameters—including demographics, anthropometrics, and medical history—to predict a composite outcome of reduced kidney function, defined using the more sophisticated age- and sex-specific eGFR thresholds. To ensure the robustness of our findings, we developed and validated three parallel models, one for each of the major eGFR equations (EKFC, CKD-EPI 2021, and CKD-EPI 2009).

## Materials and methods

### Ethics statement‌‌

This study was based on an analysis of publicly available, de-identified data from the U.S. NHANES and the KNHANES. The original NHANES protocol was approved by the National Center for Health Statistics (NCHS) Research Ethics Review Board, and the KNHANES protocol was approved by the Korea Disease Control and Prevention Agency (KDCA). All participants in both original surveys provided written informed consent. No patients or members of the public were involved in the design, conduct, or reporting of this research.

### Study population

For model derivation, we used data from the U.S. National Health and Nutrition Examination Survey (NHANES), combining the NHANES III (1988–1994) and Continuous NHANES (1999–2018) cycles to create a 30-year serial cross-sectional dataset [17]. From an initial 135,311 participants, the final analysis included 13,619 individuals after excluding those who were: (1) younger than 50 years old, (2) had a history of kidney transplant as identified by specific medication use (e.g., tacrolimus, cyclosporine), or (3) had incomplete data for the 113 exposome parameters and outcome indicators selected for analysis [18]. The study flow chart is detailed in S1 Fig.

For external validation, we used a dataset of 6,454 participants aged 50 years and older from the three most recent cycles (2021–2023) of the Korea National Health and Nutrition Examination Survey (KNHANES) [19]. Applying the derived MERWACS model to this external dataset required several data harmonization steps for predictors that were unavailable in KNHANES. Specifically, missing predictor values were imputed using a random forest algorithm (*missForest* R package) [20], and other derived variables were calculated from source data. Full details of these harmonization steps and the specific variables handled are provided in the legend of S1 Table.

We reported the study findings according to the Strengthening the Reporting of Observational Studies in Epidemiology (STROBE) [21] and the Sex And Gender Equity in Research (SAGER) guidelines [22]; the term ‘gender’ is used to reflect the binary (male/female) data collection format of the source surveys.

### Outcome of interest: Reduced kidney function

The primary outcome was a composite binary endpoint of reduced kidney function, defined by the presence of one or both of the following criteria:

1. Albuminuria: A urine albumin-to-creatinine ratio (uACR) ≥ 30 mg/g, corresponding to moderately or severely increased albuminuria.
2. Age- and Sex-Specific Low eGFR: An estimated glomerular filtration rate that fell below the lower limit of normal for a participant’s corresponding age and sex group.

To establish these eGFR thresholds, we adopted the methodology of Eriksen et al. [10], who defined normal eGFR distributions in large, healthy European populations. The lower limit of normal was defined as the 2.5th percentile of the eGFR distribution within each 5-year age and sex stratum of this healthy reference population. We chose this dynamic, percentile-based approach over a conventional fixed threshold (e.g., eGFR < 60 mL/min/1.73m^2^) to more accurately account for physiological renal senescence and distinguish it from pathological declines in kidney function. Participants meeting one or both above criteria were classified as having the outcome.

### Estimated glomerular filtration rate (eGFR) calculation

We calculated eGFR using three distinct equations to assess the robustness of our findings. This framework, involving three separate, parallel MERWACS models—one for each equation—was designed to comprehensively test our approach against different clinical standards:

1. The European Kidney Function Consortium (EKFC) equation: This equation was selected as our primary approach, as it was developed using iohexol-measured GFR, making it closely aligned with the reference data from Eriksen et al. used to define our outcome [23].
2. The Chronic Kidney Disease Epidemiology (CKD-EPI) 2021 race-free equation: This is the current recommended standard for clinical practice and research, notable for its removal of the race coefficient [24].
3. The CKD-EPI 2009 equation: This was included as a widely used historical standard to ensure comparability with a broad body of previous literature [25].

To ensure consistency across the 30-year span of the NHANES data, serum creatinine measurements were standardized prior to eGFR calculation, following established laboratory recommendations [26]. Specifically, creatinine values from NHANES III (1988–1994) were calibrated using the equation: Standardized Cr = -0.184 + 0.960 * original Cr. Values from NHANES 1999–2000 were calibrated using the equation: Standardized Cr = 0.147 + 1.013 * original Cr. Serum creatinine values from subsequent NHANES cycles were already standardized and required no recalibration.

### Candidate predictor selection

The initial pool of candidate predictors was derived from the harmonized 30-year NHANES dataset and comprised 3,069 unique variables spanning demographic, anthropometric, dietary, questionnaire, urine, serum, and chemical exposome data. We applied a multi-step filtering process to refine this list to a set of manageable, consistently measured, and non-invasive parameters suitable for feature selection.

First, we excluded variables that were not consistently measured across all survey cycles to ensure data continuity. Second, to align with our primary objective of developing an accessible, non-invasive screening tool, we removed all laboratory-based parameters (e.g., serum and urine biomarkers). Finally, we applied a data completeness filter, excluding any remaining variables with more than 20% missing values across the dataset. For example, physical activity variables, though conceptually aligned with our accessibility criteria, were excluded at this stage due to substantial missingness arising from changes in survey questionnaires across NHANES cycles (e.g., 40.0% missing for PAD440 and 65.2% for PAQ100).

This filtering process resulted in a final set of 113 candidate exposome parameters (see S1 Method) that were advanced to the formal feature selection stage for model development.

### Statistical analysis

The development and validation of our prediction model, MERWACS, were reported in accordance with the Transparent Reporting of a Multivariable Prediction Model for Individual Prognosis or Diagnosis + Artificial Intelligence (TRIPOD+AI) statement [27].

Continuous variables were presented as medians and interquartile ranges (IQRs), and categorical variables were presented as counts and percentages. Group comparisons were performed using Mann-Whitney U test, analysis of variance (ANOVA), or Kruskal-Wallis test for continuous variables, and the chi-squared test or Fisher’s exact test for categorical variables, as appropriate. All statistical tests were two tailed.

To evaluate the robustness of MERWACS, we conducted pre-specified subgroup analyses on the internal validation test set. Performance was stratified by eight key demographic and clinical scenarios: (1) age (in 5-year increments from 50 to > 90 years), (2) gender (female or male), (3) combined age and gender, (4) smoking status (defined by serum cotinine level < 14 ng/mL or ≥ 14 ng/mL), (5) ethnicity (Mexican American, other Hispanic, Non-Hispanic White, Non-Hispanic Black, or other multiracial, (6) Body Mass Index (BMI) (< 30 kg/m^2^ or ≥ 30 kg/m^2^), (7) Poverty Income Ratio (PIR): < 1, 1–2, 2–4, or >4), and (8) education level (up to high school or higher).

### Machine learning pre-processing

The NHANES derivation dataset was split into training and internal test sets using a 70:30 ratio. To ensure that the distribution of the “reduced kidney function” outcome was consistent between the two sets, we performed stratified sampling based on the outcome variable. All 113 candidate predictors in both sets were then standardized by scaling each variable to a mean of zero and a standard deviation (SD) of one. This entire pre-process pipeline, including the data split and standardization, was implemented using the *caret* package in R. As the analysis was restricted to participants with complete data for all candidate predictors, no imputation was necessary.

### Identification of significant exposome parameters

To identify the most informative predictors from the initial set of 113 exposome parameters, we performed feature selection on the training dataset using the Boruta algorithm. Boruta is a “wrapper” method, meaning it uses an underlying machine learning model—in our case, an extreme gradient boosting (XGBoost) model—to evaluate the importance of each variable [28,29]. The algorithm works by creating randomized “shadow” copies of every predictor and running an iterative competition: a real predictor is only confirmed as significant if it consistently proves more important than the best of its randomized shadow counterparts. This robust process ensures that the selected predictors have a genuinely strong and stable relationship with the outcome. The algorithm was run for a maximum of 1000 iterations to ensure a stable and exhaustive selection of features significantly associated with reduced kidney function.

Following this initial data-driven selection, final refinement was performed to ensure the resulting MERWACS tool would be practical for widespread clinical and public use. From the set of parameters identified as significant by Boruta, we excluded variables related to dietary intake. This decision was guided by the objective of creating a simple, accessible tool that does not depend on dietary recall, which can be burdensome for users and prone to measurement error. This two-stage process—data-driven selection followed by a utility-focused refinement—yielded the final set of predictors for model development.

### Development of MERWACS

To develop MERWACS, we first compared the performance of three distinct machine learning algorithms: random forest (RF) [30], a model-averaged neural network (NNET) [31], and an extreme gradient boosting (XGBoost) model [29].

The training and hyperparameter optimization process for each algorithm was embedded within a 3-times repeated 10-fold cross-validation framework to minimize sampling bias and prevent overfitting [32]. All models were trained on the training dataset using the final set of predictors identified by our feature selection process. Hyperparameters were tuned using a random search strategy within an adaptive resampling framework. This approach iteratively excluded poorly performing hyperparameter settings based on a generalized least squares (GLS) criterion, thereby improving computational efficiency.

We assessed the performance of the three candidate algorithms using three key metrics calculated across the 30 cross-validation resamples. We primarily evaluated discrimination using the area under the receiver operating characteristic curve (ROCAUC), which measures the model’s ability to distinguish between individuals with and without reduced kidney function (a value of 1.0 indicates perfect discrimination). We also assessed the area under the precision-recall curve (PRAUC), a metric particularly suited for datasets with imbalanced outcomes, where higher is better. Finally, we measured calibration using the Brier score, which quantifies the accuracy of the predicted probabilities themselves (a score of 0 indicates perfect calibration). The algorithm demonstrating the best overall performance, primarily evaluated by ROCAUC, was selected as the final model for MERWACS.

### Model calibration

To ensure the predicted probabilities from the final MERWACS model could be reliably interpreted as accurate probabilities, we applied isotonic regression as a post-processing calibration step [33]. This non-parametric method learns a monotonic function to map the model’s raw outputs to well-calibrated probabilities by minimizing the squared error between predicted and true outcomes, while preserving the rank-ordering of predictions. The optimization is defined as:

$f=arg\;\underset{f}{\text{min}}\sum_{i}{\left(y_{i}-f(p_{i})\right)}^{2}\text{ subject to }f\left(p_{i}\right)\leq f(p_{j})\text{ for all }p_{i}\leq p_{j}$

where $y_{i}$ is the true binary outcome, $p_{i}$ is the uncalibrated probability, and f is the learned optimal calibration function.

To prevent data leakage—a common issue where information from outside the training data inadvertently influences the model, leading to overly optimistic performance estimates—the isotonic regression model was fitted using only the predictions generated on the training set and their corresponding true labels, and not on the testing or validation sets. Once trained, the resulting calibration map was then applied to transform the raw outputs for all subsequent analyses. This approach improves the reliability of the predicted probabilities without degrading the underlying discriminative performance (i.e., the ability of the model to distinguish between people with vs. without reduced kidney health).

### Model performance evaluation

The predictive performance of the final MERWACS model was comprehensively assessed on three distinct datasets: the 30 cross-validation resamples from the training process, the held-out internal test set, and the KNHANES external validation dataset.

Discrimination performance was the primary focus of the evaluation. It was measured using the area under the receiver operating characteristic curve (ROCAUC) and the area under the precision-recall curve (PRAUC). To provide context, PRAUC was also reported as a ratio to the outcome prevalence in each dataset (PRAUC/prevalence).

In addition to discrimination, we evaluated model calibration by generating calibration curves and calculating the Brier score. For reporting binary classification metrics, an optimal probability cut-off was determined on the training dataset using Youden’s J statistic method. This method identifies the threshold that maximizes the sum of sensitivity and specificity. This cut-off was then used to calculate sensitivity, specificity, accuracy, and balanced accuracy (the mean of sensitivity and specificity). To quantify uncertainty, 95% confidence intervals were generated for all performance metrics using 1000 bootstrap replicates. In addition, we quantified calibration numerically using two summary metrics: the calibration-in-the-large intercept, which captures systematic bias in predicted probabilities overall (perfect value = 0; negative values indicate overprediction), and the calibration slope, which captures whether predicted probabilities are appropriately spread (perfect value = 1; values below 1 indicate overconfidence). Both metrics were estimated via logistic regression of the binary outcome on the log-odds of the calibrated predicted probabilities [34].

### Decision curve analysis

To evaluate the clinical utility of MERWACS, we conducted a decision curve analysis (DCA) on the internal test set using the primary EKFC-based outcome [35]. DCA quantifies the net benefit of a prediction model across a range of threshold probabilities, accounting for both the benefit of true positives and the harm of false positives weighted by the chosen threshold. We compared MERWACS against five strategies: (1) Screen All, (2) Screen None, (3) age-based screening, defined as all individuals aged 55 years or older, consistent with cost-effectiveness estimates for CKD screening in the general population [7], (4) hypertension-based screening, defined as a self-reported diagnosis of hypertension, and (5) a simplified KDIGO-based strategy, defined as a self-reported diagnosis of diabetes or hypertension, consistent with KDIGO 2024 screening recommendations [1].

### Model interpretation

To ensure transparency and provide insight into the model’s decision-making process, we employed two distinct explainable AI techniques for global and local interpretation.

For global interpretation, to understand the overall impact of each predictor across the dataset, we applied the SHapley Additive exPlanations (SHAP) algorithm [36]. SHAP values quantify the marginal contribution of each feature to a model’s prediction. We generated SHAP summary plots based on a sample of 2,000 individuals from the training set to visualize the magnitude and direction of each feature’s effect on the predicted probability of reduced kidney health.

For local, case-specific interpretation, we utilized the Local Interpretable Model-agnostic Explanations (LIME) algorithm [37]. LIME explains an individual prediction by fitting a simpler, interpretable model in the local vicinity of the prediction being examined. This allowed for a closer inspection of how specific feature values contributed to the final model prediction for selected individual cases, enhancing clinical interpretability.

### Sensitivity analyses

We conducted three pre-specified sensitivity analyses to evaluate the robustness of our model and methodological choices.

First, to quantify the impact of excluding dietary data, we investigated the potential value of these predictors by training an alternative model that included dietary parameters. This analysis was focused on the primary EKFC-based outcome and compared the performance of the dietary vs. non-dietary MERWACS model on the internal test set. Second, to assess the impact of data harmonization, we evaluated a model version that excluded predictors unavailable in the KNHANES dataset. This analysis was also focused on the EKFC-based outcome and was performed on both the internal and external validation datasets to ensure an equitable comparison. Finally, to provide a benchmark for our choice of a machine learning algorithm, we compared MERWACS to a baseline multivariable logistic regression model. This comparison was conducted across all three eGFR-based outcomes on both the internal and external validation datasets to comprehensively assess the performance difference between a non-linear and a linear approach.

### Software and packages

Statistical software used was R software (version 4.3.2, R Foundation for Statistical Computing) and RStudio (version 2024.9.0.375). Packages used were: tidyverse (version 2.0.0), tidyr (version 1.3.1), writexl (version 1.5.0), forcats (version 1.0.0), Boruta (version 8.0.0), caret (version 6.0-94), caretEnsemble (version 2.0.3), randomForest (version 4.7-1.1), xgboost (version 1.7.8.1), nnet (version 7.3-19), parallel (version 4.3.2), doParallel (version 1.0.17), kernelshap (version 0.7.0), shapviz (version 0.9.4), patchwork (version 1.3.0), MLmetrics (version 1.1.3), ggpubr (version 0.6.0), compareGroups (version 4.8.0), geomtextpath (version 0.1.5), pROC (version 1.18.5), yardstick (version 1.3.1), ggsci (version 3.2.0), rsample (version 1.2.1), haven (version 2.5.4), missForest (version 1.5), missForestPredict (version 1.0), viridis (version 0.6.5), ggh4x (version 0.3.0), lime (version 0.5.3), shiny (version 1.9.1), ggplot2 (version 3.5.1), highcharter (version 0.9.4), httr (version 1.4.7), jsonlite (version 1.8.8), shinythemes (version 1.2.0), RVAideMemoire (version 0.9-83-12), and dcurves (version 0.5.0).

### Data and code availability

The datasets analyzed in the current study were collected by the Centers of Disease Control and Prevention and our curated dataset is available in the Kaggle repository, https://www.kaggle.com/datasets/nguyenvy/nhanes-19882018 [18]. MERWACS model is available in the public GitHub repository: https://github.com/udaniel/MERWACS. Complete code and data to reproduce the figures is also available in the public GitHub repository: https://github.com/udaniel/MERWACS.

## Results

### Baseline characteristics of the derivation dataset

The final derivation dataset included 13,619 participants. This population of U.S. adults aged over 50 and older had a median age of 65.0 years (IQR 57.0 – 73.0); with a nearly even gender distribution (50.7% female). The median BMI was 27.9 kg/m^2^ (24.6 – 31.6) and the median poverty income ratio (PIR) was 2.6 (1.4 – 4.6). The training (n = 9,534) and internal test (n = 4,085) sets were well-balanced and largely comparable, with only statistically significant difference observed in the prevalence of self-reported congestive heart failure (5.2% in train vs. 6.1% in test; p = 0.023). A comprehensive summary of all demographic and clinical characteristics is provided in Table 1.

**Table 1:** Baseline characteristics of the NHANES derivation dataset, by train and test set‌‌ split.

	Overall (n = 13619)	Train set (n = 9534)	Test set (n = 4085)	p-value
Age (years), Median [25th;75th]	65.0 [57.0;73.0]	65.0 [57.0;73.0]	65.0 [57.0;73.0]	0.655
Gender female, No. (%)	6902 (50.7%)	4823 (50.6%)	2079 (50.9%)	0.758
Recode of reported race and Hispanic origin information, No. (%)				0.246
Mexican American	1921 (14.1%)	1334 (14.0%)	587 (14.4%)	
Other Hispanic	611 (4.5%)	449 (4.7%)	162 (4.0%)	
Non-Hispanic White	8103 (59.5%)	5682 (59.6%)	2421 (59.3%)	
Non-Hispanic Black	2386 (17.5%)	1664 (17.5%)	722 (17.7%)	
Other Race - Including Multi-Racial	598 (4.4%)	405 (4.2%)	193 (4.7%)	
Poverty income ratio (PIR), Median [25th;75th]	2.6 [1.4;4.6]	2.6 [1.4;4.6]	2.5 [1.4;4.5]	0.101
Education level - Adults 20 + , No. (%)				0.173
Less than 9th grade	2236 (16.4%)	1567 (16.4%)	669 (16.4%)	
9-11th Grade (Includes 12th grade with no diploma)	1954 (14.3%)	1382 (14.5%)	572 (14.0%)	
High School Grad/GED or Equivalent	3481 (25.6%)	2427 (25.5%)	1054 (25.8%)	
Some College or AA degree	3343 (24.5%)	2295 (24.1%)	1048 (25.7%)	
College Graduate or above	2605 (19.1%)	1863 (19.5%)	742 (18.2%)	
Systolic: Average blood pressure (mm Hg), Median [25th;75th]	131.3 [119.3;145.3]	131.3 [119.3;145.3]	131.0 [119.3;145.3]	0.562
Diastolic: Average blood pressure (mm Hg), Median [25th;75th]	71.3 [64.0;78.7]	71.3 [64.0;78.7]	72.0 [64.0;78.7]	0.256
Body Mass Index (kg/m2), Median [25th;75th]	27.9 [24.6;31.6]	27.8 [24.6;31.6]	28.0 [24.6;31.8]	0.274
Serum cotinine (ng/mL), Median [25th;75th]	0.1 [<0.1;0.6]	0.1 [<0.1;0.6]	0.1 [<0.1;0.5]	0.898
Doctor told you have diabetes, No. (%)	2340 (17.2%)	1615 (16.9%)	725 (17.7%)	0.262
Ever been told by a health professional that you had hypertension?, No. (%)	6937 (50.9%)	4833 (50.7%)	2104 (51.5%)	0.395
Ever told had congestive heart failure, No. (%)	743 (5.5%)	492 (5.2%)	251 (6.1%)	0.023
Ever told you had heart attack, No. (%)	1143 (8.4%)	785 (8.2%)	358 (8.8%)	0.323
Ever told you had a stroke, No. (%)	759 (5.6%)	533 (5.6%)	226 (5.5%)	0.925
Serum creatinine (mg/dL), Median [25th;75th]	0.9 [0.8;1.1]	0.9 [0.8;1.1]	1.0 [0.8;1.1]	0.629
Urinary albumin (ug/mL), Median [25th;75th]	8.0 [3.9;18.4]	8.0 [3.9;18.6]	8.0 [3.8;18.1]	0.787
Urinary creatinine (mg/dL), Median [25th;75th]	98.0 [55.4;147.1]	98.0 [55.4;148.0]	97.3 [55.0;146.0]	0.358
Urinary albumin-to-creatinine ratio (uACR), Median [25th;75th]	8.2 [4.9;17.9]	8.2 [4.9;17.8]	8.2 [4.9;18.2]	0.808
eGFR (EKFC), Median [25th;75th]	72.8 [60.0;84.4]	72.8 [60.1;84.5]	72.8 [59.7;84.2]	0.785
eGFR (CKD-EPI 2021), Median [25th;75th]	82.8 [68.7;95.3]	82.9 [68.8;95.3]	82.7 [68.4;95.1]	0.696
eGFR (CKD-EPI 2009), Median [25th;75th]	80.1 [66.2;92.4]	80.1 [66.3;92.5]	80.3 [65.7;92.4]	0.823
**Outcome of the study, No. (%)**				
Reduced Kidney Health (eGFR EKFC)	4814 (35.3%)	3370 (35.3%)	1444 (35.3%)	1
Reduced Kidney Health (eGFR CKD-EPI2021)	3550 (26.1%)	2483 (26.0%)	1067 (26.1%)	0.943
Reduced Kidney Health (eGFR CKD-EPI 2009)	3821 (28.1%)	2659 (27.9%)	1162 (28.4%)	0.522

Abbreviation: NHANES, National Health and Nutrition Examination Survey.

### Prevalence of reduced kidney function

The prevalence of our primary composite outcome, reduced kidney function, varied substantially across the three eGFR equations used to define the age- and sex-specific low eGFR component. The highest prevalence was observed using the EKFC equation, which classified 4,814 participants (35.3%) as having reduced kidney health. The prevalence was lower when using the CKD-EPI 2021 definition (3,550 participants; 26.1%) and the CKD-EPI 2009 definition (3,821 participants; 28.1%). These differences in classification were statistically significant (Cochran’s Q test, p < 0.001). When applying NHANES survey weights to project to the U.S. population, these prevalences corresponded to 31.7%, 22.0%, 25.7%, respectively. Detailed data for the outcome are available in Table 1.

It is important to note that these high prevalence rates are expected. First, the eGFR threshold is the 2.5^th^ percentile derived from a separate, healthy European population; it is anticipated that a much larger proportion of a general, real-world population like NHANES would fall below this cutoff. Second, our outcome is a composite of either low eGFR or the presence of albuminuria, which further increases the total prevalence.

To further characterize the composite outcome, we decomposed the prevalence into mutually exclusive contributions from each criterion. For the primary EKFC-based model in NHANES, 19.0% of participants were classified by low eGFR alone, 10.4% by elevated uACR alone, and 6.0% by both criteria simultaneously. The eGFR-only contribution was lower for the CKD-EPI 2021 (9.7%) and CKD-EPI 2009 (11.7%) equations, reflecting differences in equation sensitivity to age-related eGFR decline. Full decomposition results for both datasets are provided in S2 Table.

**Fig 1:**
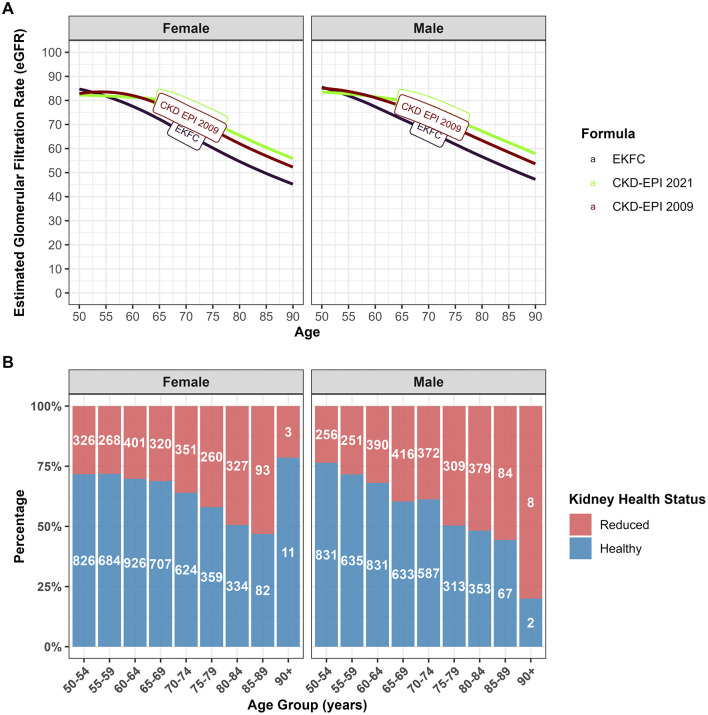
Overview of estimated glomerular filtration rate (eGFR) and prevalence of reduced kidney health in the NHANES derivation dataset. **(A)** This panel illustrates the trend of eGFR distribution across age, stratified by gender, visualized using a Locally Estimated Scatterplot Smoothing (LOESS) method. Each of the three lines represents a different eGFR calculation: the European Kidney Function Consortium (EKFC), the Chronic Kidney Disease Epidemiology Collaboration (CKD-EPI) 2021, and the CKD-EPI 2009 equation. **(B)** This panel shows the proportion of participants classified as having reduced kidney health (red) or healthy status (blue) across 5-year age groups, stratified by gender. The outcome classification in this panel is based on a composite definition of either a uACR ≥ 30 mg/g or an eGFR below the 2.5^th^ percentile of a healthy reference population from Eriksen et al [10]. As the NHANES population is a general population and the outcome is a composite, the observed prevalence of reduced kidney health is expected to be higher than 2.5%. The numbers within each bar represent the absolute count of participants.

Fig 1A visually contrasts the different continuous eGFR distributions generated by the three equations across age groups. Subsequently, Fig 1B illustrates the distribution of the binary outcome (healthy vs. reduced kidney status) using the EKFC-based definition, stratified by age group and gender.

### Predictor importance

We used the Boruta algorithm to identify the most influential predictors of reduced kidney health from the initial set of 113 exposome parameters. The analysis was performed separately for outcomes defined by each of the three eGFR equations.

**Fig 2:**
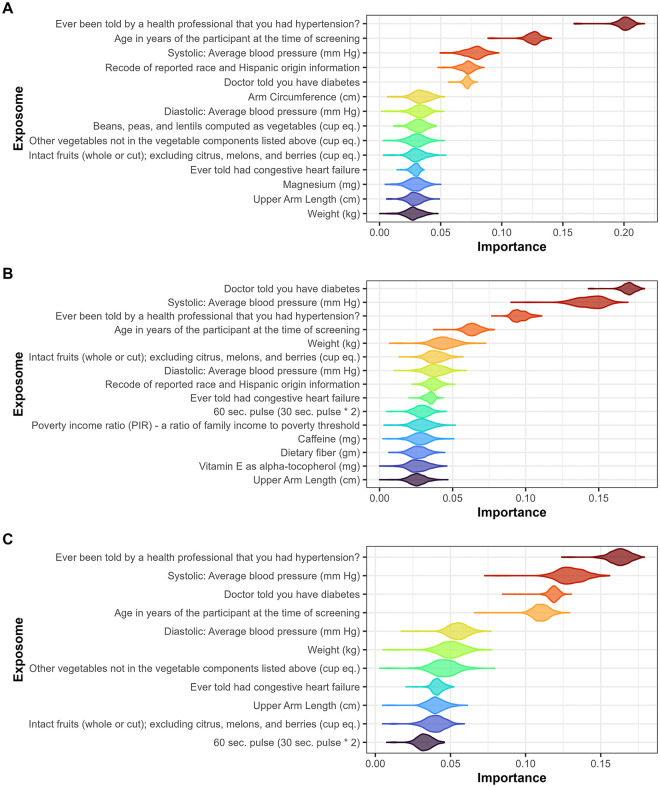
Predictor importance for reduced kidney health as determined by the initial data-driven Boruta algorithm. The violin plots illustrate the distribution of importance scores for the exposome parameters identified as significant by the Boruta feature selection algorithm. Predictors are ranked vertically by their median importance score. This feature selection process was performed independently for the primary outcome as defined by each of the three different eGFR equations. The dietary parameters shown here were subsequently excluded in a final refinement step to maximize clinical usability. **(A)** The 14 predictors identified as significant when using the European Kidney Function Consortium (EKFC) equation. **(B)** The 15 predictors identified as significant when using the Chronic Kidney Disease Epidemiology Collaboration (CKD-EPI) 2021 equation. **(C)** The 11 predictors identified as significant when using the CKD-EPI 2009 equation.

As shown in Fig 2, the algorithm selected 14, 15, and 11 significant parameters for the EKFC, CKD-EPI 2021, and CKD-EPI 2009 equations, respectively. Several key predictors were consistently identified as important across all three outcome definitions, including a history of hypertension, a history of diabetes, a history of congestive heart failure, age, average systolic and diastolic blood pressure, and weight. Other parameters were uniquely important to specific eGFR equations, such as ethnicity for the CKD-EPI 2021 equation and arm circumference for the EKFC equation. The dietary parameters identified by Boruta were subsequently excluded from the final model derivation to maximize accessibility, as described in the Methods.

### Algorithm selection

We assessed three candidate machine learning models—random forest, model-averaged neural network (NNET), and XGBoost—during the cross-validation process. The XGBoost consistently achieved the highest mean discrimination performance across all three eGFR equation-based outcomes.

For the primary EKFC equation-based outcome, the mean ROCAUC for XGBoost was 0.700 (SD 0.017), compared to 0.699 (SD 0.017) for NNET and 0.686 (SD 0.017) for random forest. However, as is common in model comparison studies, the 95% confidence intervals for these performance metrics showed overlap, indicating that the differences were not statistically significant. Similar trends were observed for the outcomes defined by the CKD-EPI 2021 and CKD-EPI 2009 equations.

Based on its highest point-estimate performance, the XGBoost algorithm was selected for the final MERWACS model. Detailed cross-validation results are available in S3-S5 Tables, and a list of the final model hyperparameters is provided in S6 Table.

### MERWACS performance on internal validation

On the held-out internal test set (n = 4,085), MERWACS demonstrated moderate and consistent discrimination performance for predicting reduced kidney health across all three outcome definitions. The model achieved a ROCAUC of 0.688 (95% CI 0.672 – 0.705) for the EKFC-defined outcome, with comparable results for the CKD-EPI 2021 outcome (ROCAUC 0.699; 95% CI 0.680 – 0.718) and the CKD-EPI 2009 outcome (ROCAUC 0.684; 95% CI 0.666 – 0.702).

**Fig 3:**
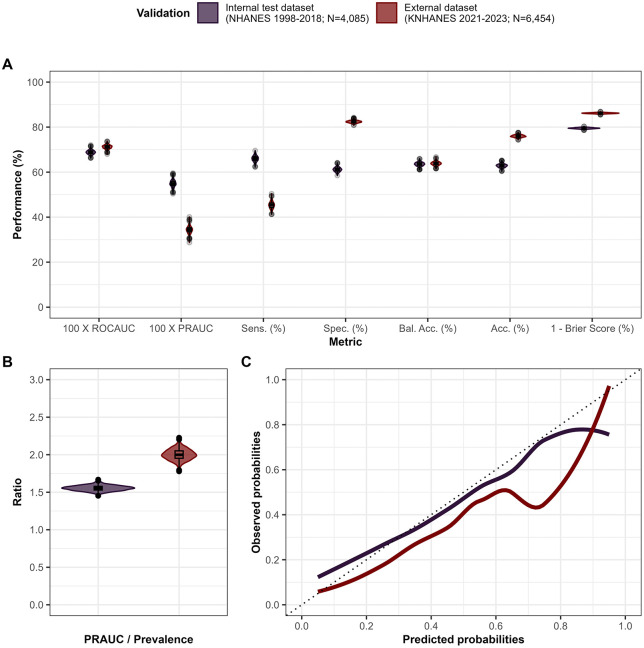
Discrimination and calibration performance of MERWACS for the EKFC-defined outcome. The figure compares the performance of the final MERWACS model on the internal test dataset (NHANES 1999–2018; n = 4,085) and the external validation dataset (KNHANES 2021–2023; n = 6,454). The outcome is defined using the European Kidney Function Consortium (EKFC) equation. Discrimination performance was assessed using 1000 times bootstrapping. **(A)** Discrimination performance across seven key metrics. All box plots comprise the median line, the box indicates the interquartile range (IQR), whiskers denote the rest of the data distribution and outliers are denoted by points greater than ±1.5 × IQR. The surrounding violin plots show the distribution of estimates from bootstrap replicates. The binary metrics (Sensitivity, Specificity, Balanced Accuracy, Accuracy) are calculated using the optimal probability cut-off of 0.352, as determined by Youden’s J statistic. **(B)** The ratio of the PRAUC to the prevalence of the outcome in each dataset. This metric provides a measure of performance relative to a random baseline. **(C)** Calibration plot comparing the predicted probabilities generated by the MERWACS model (x-axis) against the observed probabilities (y-axis). The dotted diagonal line represents perfect calibration, where predicted and observed probabilities are identical. Abbreviations: MERWACS, Machineborne Early Renal Warning And Control System; ROCAUC, area under the receiver operating characteristic curve; PRAUC, area under the precision-recall curve; Sens., sensitivity; Spec., specificity; Bal. Acc., balanced accuracy; Acc., accuracy.

The model’s performance across other metrics, including calibration, was also robust. For the primary EKFC-based model, this included a PRAUC of 0.549 (95% CI 0.523 – 0.576) and a Brier score of 0.205 (95% CI 0.200 – 0.210). Calibration-in-the-large metrics for the primary EKFC-based model confirmed good overall calibration on the internal test set, with an intercept of -0.010 (95% CI -0.080 to 0.059) and a slope of 0.754 (95% CI 0.673 to 0.838), indicating near-zero systematic bias but modest overconfidence in predicted probabilities. Age-stratified calibration metrics are provided in S7 Table. A comprehensive visual summary of the model’s discrimination—including sensitivity, specificity, and balanced accuracy at the optimal cut-off—and its calibration performance for the EKFC outcome is presented in Fig 3. Corresponding performance plots for the CKD-EPI 2021 and CKD-EPI 2009 outcomes, demonstrating similar performance, are provided in S2 Fig and S3 Fig, respectively.

### External validation of MERWACS

The MERWACS model was tested on an external validation dataset of 6,454 participants from the KNHANES survey in South Korea. This population had a median age of 64.0 years (IQR 57.0 – 72.0) and was 56.4% female, with a median BMI of 24.1 kg/m^2^ (22.0 – 26.2). The prevalence of reduced kidney health was 17.2%, 14.2%, and 15.4% for outcomes defined by the EKFC, CKD-EPI 2021, and CKD-EPI 2009 equations, respectively. Decomposition of the composite outcome in KNHANES revealed that the uACR component was the predominant driver of classification, with 9.4% of participants classified by elevated uACR alone compared to 4.9% by low eGFR alone (EKFC-based model). Full decomposition results are provided in S2 Table. Detailed characteristics of this validation population are in S1 Table.

In this independent South Korean dataset, MERWACS demonstrated moderate discrimination performance that was consistent with the results from the internal validation. The model achieved a ROCAUC of 0.712 (95% CI 0.696 – 0.729) for the EKFC-defined outcome, 0.728 (95% CI 0.710 – 0.745) for the CKD-EPI 2021 outcome, and 0.717 (95% CI 0.699 – 0.734) for the CKD-EPI 2009 outcome. Detailed discrimination and calibration plots for the external validation are presented alongside the internal validation results in Fig 3, S2 Fig, and S3 Fig, confirming the model’s generalizability. Calibration-in-the-large metrics indicated a systematic tendency toward overprediction in the KNHANES population, with an overall intercept of -0.441 (95% CI -0.509 to -0.374), consistent across all age groups. The calibration slope of 0.957 (95% CI 0.865 to 1.05) was close to 1, suggesting the spread of predicted probabilities was appropriate. Full age-stratified calibration metrics are provided in S7 Table.

### Model interpretation

#### Global predictor importance (SHAP).

**Fig 4:**
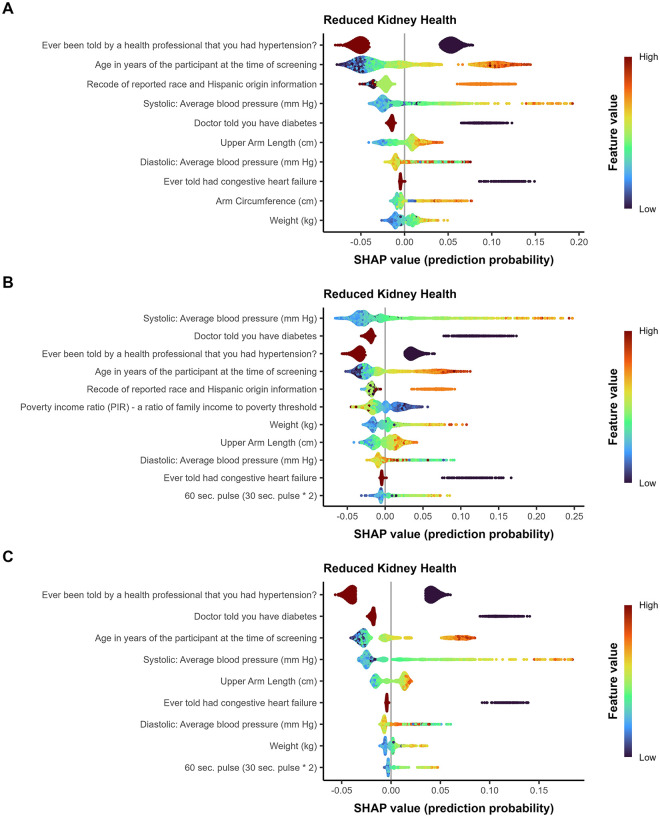
Global predictor importance as visualized by SHAP summary plots. The figure presents SHapley Additive exPlanations (SHAP) summary plots, illustrating the overall importance and impact of each predictor on the MERWACS model output. Each plot is based on a sample of 2,000 participants from the training dataset. Predictors are ranked vertically by their mean absolute SHAP value. The SHAP value on the x-axis represents the predictor’s impact on the model’s output probability; positive values increase the predicted probability of reduced kidney health. The color of each point indicates the feature’s original value, ranging from low (blue) to high (red). For categorical variables, the numerical coding is as follows: Gender: Male = 1 (low), Female = 2 (high); History of hypertension, diabetes, or congestive heart failure: “Yes” = 1 (low), “No” = 2 (high); Ethnicity: Mexican American = 1, Other Hispanic = 2, Non-Hispanic White = 3, Non-Hispanic Black = 4, and Other Race = 5 (high). **Panel A** shows the analysis using the European Kidney Function Consortium (EKFC) equation, **Panel B** uses the Chronic Kidney Disease Epidemiology Collaboration (CKD-EPI) 2021 equation, and **Panel C** uses the CKD-EPI 2009 equation. Abbreviation: MERWACS, Machineborne Early Renal Warning And Control System.

To understand the overall impact of each predictor on model output, we generated SHAP summary plots (Fig 4). Across all three eGFR equation-based models, four predictors consistently demonstrated the most significant influence on the prediction of outcome: a history of hypertension, age, a history of diabetes, and average systolic blood pressure. The SHAP plots showed that for these predictors, values associated with higher clinical risk (e.g., older age, presence of a comorbidity, higher average systolic blood pressure) consistently drove the model’s prediction towards a higher probability of reduced kidney health.

#### Local, case-specific interpretation of predicted probability (LIME).

**Fig 5:**
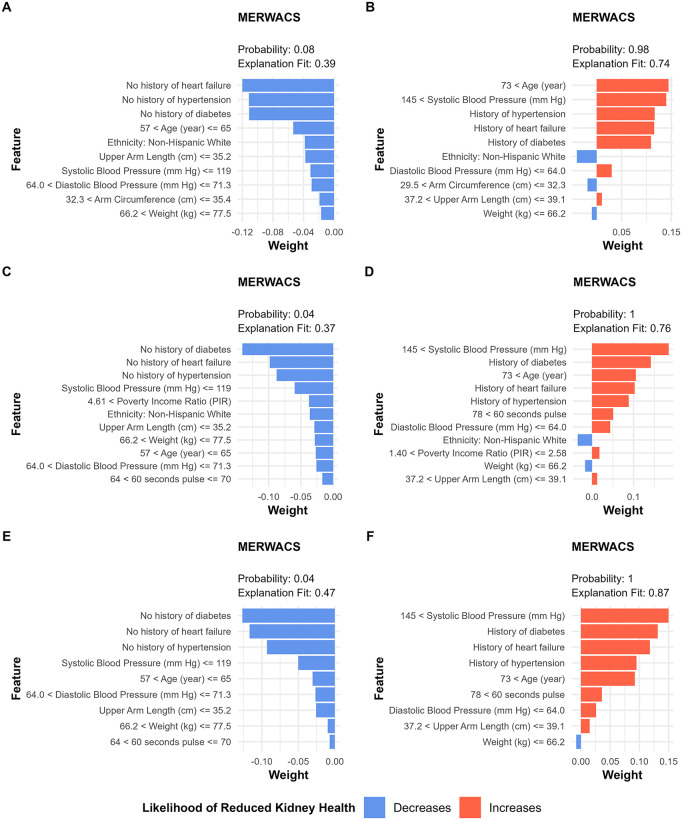
Case-specific interpretations of MERWACS predictions using LIME. The figure shows Local Interpretable Model-agnostic Explanations (LIME) for two representative individuals, demonstrating how the model’s prediction is driven by their unique features across the three different eGFR equations. The left column **(A, C, E)** shows the analysis for a single low-risk individual, while the right column **(B, D, F)** shows the analysis for a single high-risk individual. For each panel, features with blue bars act as protective factors that decrease the predicted probability of “Reduced Kidney Health”, while red bars indicate risk factors that increase it. The length of each bar is proportional to the magnitude of the feature’s contribution. The analysis for each individual is repeated for the three eGFR equations: 1) Panels A and B use the European Kidney Function Consortium (EKFC) equation; 2) Panels C and D use the Chronic Kidney Disease Epidemiology Collaboration (CKD-EPI) 2021 equation; and 3) Panels E and F use the CKD-EPI 2009 equation. Abbreviation: MERWACS, Machineborne Early Renal Warning And Control System; eGFR, estimated glomerular filtration rate.

To illustrate how MERWACS provides personalized explanations for its predictions, Fig 5 presents LIME plots for two individuals with opposing risk profiles across the three different eGFR equation-based models. The plots show how the model’s prediction for a specific person is driven by their unique combination of risk and protective factors. For individuals with a low predicted probability of reduced kidney health (Figs 5A, 5C, 5E), the LIME analyses consistently highlight protective factors—such as the absence of hypertension and diabetes—as the primary features decreasing their predicted probability. Conversely, for individuals with a high predicted probability (Figs 5B, 5D, 5F), the analyses identify risk factors like older age and the presence of comorbidities as the key drivers that increased the predicted probability. These examples demonstrate the model’s capacity to deliver case-specific, interpretable predictions regardless of the underlying eGFR equation used.

### Performance in subpopulations

To assess the robustness of MERWACS, we evaluated its performance across eight demographic and clinical subgroups on the internal test set, using the EKFC-based model.

MERWACS demonstrated moderate and generally consistent discrimination performance across all evaluated subpopulations. The model’s performance was notably strong in participants in their 60s, achieving a ROCAUC of 0.707 (95% CI 0.667 – 0.749) for those aged 65–69. Similarly, performance was higher among individuals with lower education levels (ROCAUC 0.705; 95% CI 0.683 – 0.727). While performance varied slightly across different ages, genders, and socioeconomic strata, no subgroup demonstrated a critical drop in predictive ability. Detailed performance metrics for all subpopulations are available in S8 Table.

### Sensitivity analysis

#### Impact of dietary predictors.

The final MERWACS model was designed for maximum accessibility and ease of use by excluding dietary predictors, which are often difficult for individuals to track accurately. To quantify the impact of this design choice on performance, we tested an alternative model that included the dietary parameters identified by Boruta. On the internal test set, this more complex model showed no meaningful improvement in discrimination (ROCAUC 0.690; 95% CI 0.670 – 0.706) compared to the final, streamlined MERWACS model (ROCAUC 0.688; 95% CI 0.672 – 0.705). This finding validates our strategy of prioritizing a practical, user-friendly tool.

#### Impact of predictors unavailable in external validation.

To ensure a fair comparison with the KNHANES dataset, we also evaluated a model version that excluded the two predictors unavailable in that dataset: a history of congestive heart failure and arm length. On the internal test set, this reduced model performed comparable to the full model (ROCAUC 0.686 vs. 0.688, respectively). When applied to the external KNHANES dataset, this version achieved a ROCAUC of 0.711 (95% CI 0.694 – 0.727), confirming that the model’s performance on the external dataset is robust and not substantially impacted by the harmonization of a few unavailable predictors.

#### Performance against a baseline linear model.

As a benchmark, we compared MERWAC’s performance to that of a traditional logistic regression model. On the internal test set, the logistic regression model achieved ROCAUCs of 0.683, 0.692, and 0.675 for the EKFC, CKD-EPI 2021, and CKD-EPI 2009 outcomes, respectively. On the external validation dataset, the logistic regression model achieved ROCAUCs of 0.712, 0.725, and 0.717 for the same outcomes. In both validation sets, the MERWACS XGBoost model demonstrated consistently, albeit modestly, higher discrimination performance compared to this linear baseline across all three outcome definitions.

### Decision curve analysis

**Fig 6:**
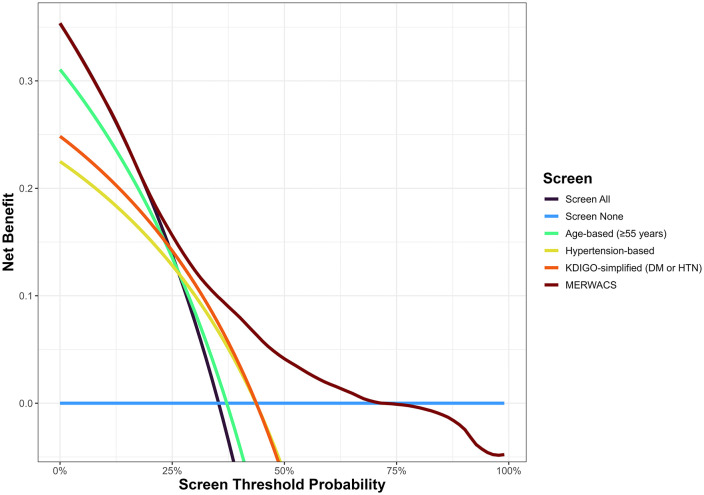
Decision curve analysis of MERWACS for the primary EKFC-based outcome. We performed a decision curve analysis (DCA) to assess the clinical utility of MERWACS in identifying individuals with reduced kidney health. DCA compared six strategies: (1) Screen All: screening all individuals, (2) Screen None: screening no individuals, (3) Age-based: screening all individuals aged ≥55 years, consistent with cost-effectiveness estimates for CKD screening (De Nicola et al., 2025), (4) Hypertension-based: screening individuals with a self-reported diagnosis of hypertension, (5) KDIGO-simplified: screening individuals with a self-reported diagnosis of diabetes or hypertension, consistent with KDIGO 2024 recommendations, and (6) MERWACS: selecting individuals for screening based on the calibrated predicted probability. The analysis accounted for false positive and false negative rates to determine the net benefit of each strategy. Abbreviations: DM, diabetes mellitus; EKFC, European Kidney Function Consortium; HTN, hypertension; KDIGO, Kidney Disease Improving Global Outcomes; MERWACS, Machineborne Early Renal Warning And Control System.

To evaluate the clinical utility of MERWACS, we conducted a decision curve analysis comparing MERWACS against five reference strategies: Screen All, Screen None, age-based screening (≥55 years), hypertension-based screening, and a simplified KDIGO-based strategy (known diabetes or hypertension). As shown in Fig 6, MERWACS provided consistently higher net benefit than all three active comparator strategies across the full range of threshold probabilities. Age-based and Screen All strategies declined rapidly, reaching negative net benefit around 35%. The KDIGO-simplified and hypertension-based strategies went negative around 50%, while MERWACS maintained positive net benefit up to approximately 75%.

### MERWACS online application

To translate our findings into a practical tool, we developed MERWACS, a ready-to-use, open access online application for both individuals and clinicians (S1 Movie). This application allows users to input a set of 12 non-invasive parameters—such as age, BMI, weight, and history of hypertension—to calculate a personalized probability of having reduced kidney health. The resulting predicted probability is immediately visualized using an intuitive gauge chart.

To enhance usability and promote trust in the model, the application incorporates two key features for interactive interpretation. First, a “What-If” scenario enables users to adjust their own parameters to prospectively observe the impact on their predicted probability. Second, individualized LIME plots are generated to visualize which specific factors are most influential in driving a person’s predicted probability higher or lower. These features provide a layer of interpretability that is crucial for clinical applicability. S4 Fig provides examples of the application’s user interface and output. The MERWACS tool is publicly available at: https://dtu-quantitative-sustainability-assessment.shinyapps.io/MERWACS/.

## Discussion

In this study, we developed and validated MERWACS, a non-invasive machine-learning system that uses easily accessible parameters to facilitate the identification of adult and elderly individuals eligible for chronic kidney disease screening. By defining its outcome with a composite of albuminuria and age- and sex-specific dynamic eGFR thresholds, MERWACS moves beyond traditional fixed cutoffs to more accurately distinguish pathological renal decline from healthy aging. Given that we deliberately excluded biomarkers of primary kidney disease to prioritize accessibility, the model’s moderate discrimination (ROCAUC ~0.7) was an expected outcome. Nonetheless, our study confirmed that MERWACS achieved moderate but consistent discrimination and calibration across internal, external validation in a non-U.S. population, and diverse clinical subpopulations.

Current CKD screening guidelines from bodies like KDIGO rightly focus on individuals with major risk factors such as hypertension and diabetes [1]. However, existing risk calculators are not designed for the specific purpose of initial, non-invasive public screening. For example, the widely used Kidney Failure Risk Equation (KFRE) is a prognostic tool used to predict the progression of kidney failure in patients already diagnosed with CKD, rather than an initial screening tool for the undiagnosed general population [38]. Other tools, like the CDC’s SCORED model or the CKD Prognosis Consortium (CKD-PC) equation [39,40], require laboratory data (e.g., proteinuria, existing eGFR) and are designed for use by physicians with access to medical records. MERWACS fills a distinct and critical gap by providing a public-facing pre-screening tool that requires no prior lab tests, empowering individuals to assess their predicted probability and engage with the healthcare system for definitive testing.

It is important to contextualize what a screen-positive MERWACS result means. The tool is not designed to diagnose CKD under KDIGO criteria, which requires persistent markers of kidney damage for more than three months [1]. Rather, a high predicted probability should be interpreted as a signal warranting clinical evaluation, including measurement of eGFR and uACR, which then feeds into formal KDIGO staging, positioning MERWACS explicitly upstream of existing clinical frameworks and complementary to them. Consistent with established screening principles, the tool prioritizes sensitivity over specificity to minimize missed cases at the population level [41], accepting a higher referral rate as a low-cost, low-risk trade-off relative to undiagnosed CKD.

In practice, MERWACS adds value in three settings: (1) identifying individuals without a formal diagnosis of diabetes or hypertension but with a high cumulative burden of continuous risk factors; (2) supporting triage in non-clinical or low-resource environments where laboratory testing is unavailable; and (3) empowering individuals to self-assess and initiate informed clinical conversations. In each scenario, MERWACS prompts evaluation rather than replacing it. The observed discrimination (ROCAUC ~0.68–0.73) is operationally appropriate for this pre-screening role, consistent with other validated non-invasive CKD screening tools [39], and reflects a deliberate trade-off that prioritizes sensitivity over specificity in line with established screening principles [41].

A common barrier to the clinical adoption of machine learning models is their perceived “black box” nature. A key strength of MERWACS is therefore its interpretability. Our feature importance analysis confirms that the model’s predictions are predominantly driven by well-established clinical risk factors for CKD, such as a history of hypertension, diabetes, older age, and elevated blood pressure [1,3]. These are the same foundational predictors used in the existing epidemiological literature and in established clinical tools [38–40], although MERWACS uses them for a different screening purpose. This alignment with known pathophysiology is critical, as it demonstrates that our model has learned clinically plausible relationships from the data, rather than relying on spurious correlations. Furthermore, the primary rationale for adopting a non-linear XGBoost model over logistic regression lies in its flexibility to capture complex, non-linear predictor interactions and its compatibility with SHAP and LIME explainability frameworks, which are essential for clinical interpretability and user trust. The modest but consistent discrimination advantage over logistic regression across both internal and external validation datasets provides additional, though secondary, support for this choice. By providing this level of transparency, MERWACS is positioned as a trustworthy tool to support shared decision-making.

A key strength of MERWACS is its design philosophy, which combines a sophisticated outcome definition with accessible predictors. By using the 2.5^th^ percentile of eGFR for specific age and sex groups, our model avoids the well-known pitfall of misclassifying healthy elderly individuals while being more sensitive to early kidney function loss in younger adults [10]. Furthermore, the inclusion of albuminuria—a potent but underutilized predictor—aligns our approach with contemporary cardiovascular risk calculators like the AHA/ACC PREVENT tool, enhancing its utility for broader health awareness where CKD is a major risk multiplier [11,42,43]. The data-driven feature selection process also identified less conventional but clinically plausible predictors, exemplified by the inclusion of mid-arm circumference—a known surrogate for adiposity in older adults that has previously been associated with CKD [44]. Similarly, the model’s identification of resting heart rate as a predictor aligns with recent findings; elevated resting heart rate is associated with an increased prevalence of CKD, especially among individuals with diabetes [45], and has also been identified as a predictor of age-related decline in kidney function [46].

The credibility of this framework is underscored by its rigorous validation [47]. We demonstrated its robustness by showing comparable performance across three different, commonly used eGFR equations (EKFC, CKD-EPI 2021, and CKD-EPI 2009). Crucially, the model’s performance was successfully validated in an external South Korean dataset (KNHANES), demonstrating strong generalizability to a geographically and ethnically distinct population. This is particularly important given the urgent need for accessible screening tools to guide the use of new, effective therapies like SGLT2 inhibitors, which have shown efficacy even in elderly populations [48,49]. Finally, by developing an interactive online application, we have bridged the research-to-practice gap, creating a tangible tool for patient education and shared decision-making, not just an abstract model.

We acknowledge this study has several limitations. First, while MERWACS demonstrated consistent performance, its discrimination was moderate (ROCAUC ~0.70). This level of performance is not sufficient for a standalone diagnostic tool. However, this was an intentional trade-off. The model was specifically designed for accessibility by deliberately excluding powerful laboratory predictors like serum creatinine. Its intended role is not to replace lab tests but to function as a cost-effective, population-level pre-screening tool to identify individuals who would benefit most from a definitive clinical evaluation. Second, our study utilized a cross-sectional design. Consequently, MERWACS predicts the probability of prevalent (existing) reduced kidney function, not incident (future) disease. While this design is highly valuable for its primary goal of identifying the large number of undiagnosed cases currently in the population, it cannot establish causality or predict the short- to mid-term risk of developing CKD. Future research should involve longitudinal validation to determine if the MERWACS prediction can also identify individuals at high risk for incident (future) CKD. This could be practically achieved by linking the NHANES survey data to long-term follow-up data from the Centers for Medicare & Medicaid Services (CMS) to ascertain outcomes such as new diagnoses of CKD, end-stage renal disease, or the initiation of dialysis. Third, while we significantly strengthened the study by externally validating it in a South Korean dataset, the model’s generalizability to other global populations (e.g., European, African) remains unknown. Furthermore, the very definition of our outcome relied on age- and sex-specific eGFR thresholds derived from healthy European cohorts. The applicability of these specific percentile cutoffs to non-European ethnic populations is uncertain and represents a limitation, particularly for populations of African or South Asian origin where eGFR distributions and creatinine metabolism may differ systematically. Validating the outcome definition itself, not just the model, in these populations is an important future step. Additionally, ethnicity (one of the predictors in MERWACS) is a variable that KNHANES does not collect, as it surveys an ethnically homogeneous Korean population [19]. This structural difference limits our ability to assess predictor stability across ethnic groups, and future validation in ethnically diverse non-U.S. populations would be needed to fully characterize this source of variability. However, we mitigated this concern by demonstrating the model’s consistent performance across three different eGFR equations, including the race-free CKD-EPI 2021 equation, which suggests the final MERWACS prediction is robust and less dependent on the potential biases of any single equation [50]. Finally, the external validation required imputation of several predictors that were unavailable in the KNHANES dataset. This approach assumes that the correlational structure between predictors is similar enough between the U.S. and South Korean populations to allow for valid imputation. However, to address this, our pre-specified sensitivity analysis showed that the model was robust to the exclusion of these variables, increasing our confidence that their imputation did not substantially affect the external validation results. Importantly, our pre-specified sensitivity analysis demonstrated that excluding these imputed predictors entirely yielded virtually identical external validation performance (ROCAUC 0.711 vs. 0.712), providing direct empirical evidence that the imputation did not meaningfully inflate the reported external validation results. Nevertheless, validating the full, unmodified model in an external dataset where all predictors are directly measured would be an important future step.

Translating MERWACS into practice raises important implementation considerations. First, regarding safeguards, the online application is explicitly framed as a pre-screening aid rather than a diagnostic tool, and results are accompanied by a recommendation to seek clinical evaluation for individuals with elevated predicted probabilities. To minimize misinterpretation, probability outputs are presented in tiered categories with plain-language guidance rather than as raw numerical values alone. Second, regarding communication, the tool is designed for dual use: individuals can self-assess and arrive at clinical encounters with greater awareness of their kidney health status, while clinicians can use the predicted probability to support and document screening decisions. Third, regarding integration with healthcare systems, MERWACS is most naturally positioned at points of care with broad population reach but limited laboratory access, such as community pharmacies, telemedicine platforms, or public health screening programs. Future implementation should involve prospective evaluation of whether MERWACS-guided triage increases the rate of appropriate laboratory testing and early CKD detection in these settings.

In summary, MERWACS is a novel, non-invasive tool that addresses key gaps in current CKD screening strategies. By integrating multiple easily measured risk factors, employing a biologically nuanced outcome definition, and being translated into a usable application, it offers a personalized and accessible method for identifying individuals for screening. By placing a validated, interpretable predicted probability directly into the hands of individuals and clinicians, MERWACS can prompt crucial, timely conversations and evaluations that are essential for improving kidney and cardiovascular health outcomes.

## Supporting information

S1 Method List of a final set of candidate parameters. (DOCX)

S1 Table Baseline characteristics of the KNHANES external validation dataset. Abbreviation: KNHANES, Korea National Health and Nutrition Examination Survey. Data harmonization notes: The MERWACS model requires predictors that were not uniformly collected in the KNHANES 2021–2023 dataset. *To enable external validation, the following steps were taken: 1) Ethnicity is not collected in KNHANES. To ensure model compatibility, all participants were assigned to the ‘Other Race – Including Multi-Racial’ category. 2) History of congestive heart failure, arm length, and arm circumference, which were missing in some or all KNHANES cycles, were imputed. This was achieved using a random forest imputation model (missForest R package) that was pre-trained on the complete NHANES training dataset; this pre-trained model was then applied to the KNHANES data to predict the missing values, ensuring no outcome information from the KNHANES dataset was used in its own imputation process. 3) The poverty income ratio (PIR) was calculated from household income and national poverty metrics provided by Statistics Korea (KOSTAT). All other variables were directly available.
(DOCX)

S2 Table Decomposition of the composite reduced kidney function outcome by individual criterion, for NHANES and KNHANES datasets across three eGFR equations. The table presents the number and percentage of participants classified into four mutually exclusive groups based on the two components of the composite outcome: low eGFR (below the age- and sex-specific 2.5th percentile from Eriksen et al., 2020) and elevated uACR (urine albumin-to-creatinine ratio ≥ 30 mg/g). The composite row corresponds to the primary outcome used in the manuscript (one or both criteria met) and serves as an internal consistency check against the prevalence figures reported in Table 1. Abbreviations: CKD-EPI, Chronic Kidney Disease Epidemiology Collaboration; EKFC, European Kidney Function Consortium; KNHANES, Korea National Health and Nutrition Examination Survey; NHANES, National Health and Nutrition Examination Survey; uACR, urine albumin-to-creatinine ratio.
(DOCX)

S3 Table Cross-validation results of the three ML algorithms (EKFC formula). Abbreviations: ML, machine learning; EKFC, European Kidney Function Consortium; ROCAUC, area under the receiver operating characteristic curve; PRAUC, area under the precision-recall curve; SD, standard deviation.
(DOCX)

S4 Table Cross-validation results of the three ML algorithms (CKD-EPI 2021 formula). Abbreviations: ML, machine learning; CKD-EPI, Chronic Kidney Disease Epidemiology; ROCAUC, area under the receiver operating characteristic curve; PRAUC, area under the precision-recall curve; SD, standard deviation.
(DOCX)

S5 Table Cross-validation results of the three ML algorithms (CKD-EPI 2009 formula). Abbreviations: ML, machine learning; CKD-EPI, Chronic Kidney Disease Epidemiology; ROCAUC, area under the receiver operating characteristic curve; PRAUC, area under the precision-recall curve; SD, standard deviation.
(DOCX)

S6 Table Hyperparameters tuning and results for the three ML algorithms. Abbreviations: ML, machine learning; eGFR, estimated glomerular filtration rate; EKFC, European Kidney Function Consortium; CKD-EPI, Chronic Kidney Disease Epidemiology.
(DOCX)

S7 Table Calibration-in-the-large metrics for MERWACS across three eGFR equations, overall and stratified by age group, for internal and external validation datasets. Calibration intercept and slope for the MERWACS model computed for the internal test set (NHANES 1988–2018; n = 4,085) and external validation dataset (KNHANES 2021–2023; n = 6,454), overall and within each 5-year age stratum, for all three eGFR equation-based models. Values are presented as estimate (95% confidence interval). Estimates for the 85–89 and 90+ strata in the internal dataset should be interpreted with caution due to small sample sizes. In the 90+ stratum, the upper confidence interval for the CKD-EPI 2009 calibration slope could not be estimated because the sparse data prevented the profile likelihood from converging to an upper bound. No external estimates are available for these age groups as KNHANES participants were aged up to 80 years. Abbreviations: EKFC, European Kidney Function Consortium; CKD-EPI, Chronic Kidney Disease Epidemiology Collaboration; NHANES, National Health and Nutrition Examination Survey; KNHANES, Korea National Health and Nutrition Examination Survey.
(DOCX)

S8 Table Internal validation of MERWACS across various subpopulations and scenarios. Abbreviation: MERWACS, Machineborne Early Renal Warning And Control System; ROCAUC, area under the receiver operating characteristic curve; PRAUC, area under the precision-recall curve.
(DOCX)

S1 Fig Flowchart detailing the number of participants involved in the study. (DOCX)

S2 Fig Discrimination and calibration performance of MERWACS in predicting reduced kidney health status using CKD-EPI 2021 equation. The figure compares the performance of the MERWACS model on the internal test and external validation datasets for the outcome defined using the CKD-EPI 2021 equation. (A) Discrimination performance across key metrics, calculated using an optimal probability cut-off of 0.252. (B) PRAUC-to-prevalence ratio. (C) Calibration plot. Abbreviations: MERWACS, Machineborne Early Renal Warning And Control System; CKD-EPI, Chronic Kidney Disease Epidemiology; ROCAUC, area under the receiver operating characteristic curve; PRAUC, area under the precision-recall curve; Sens., sensitivity; Spec., specificity; Bal. Acc., balanced accuracy; Acc., accuracy.
(DOCX)

S3 Fig Discrimination and calibration performances of MERWACS in predicting reduced kidney health status using CKD-EPI 2009 equation. The figure compares the performance of the MERWACS model on the internal test and external validation datasets for the outcome defined using the CKD-EPI 2009 equation. (A) Discrimination performance across key metrics, calculated using an optimal probability cut-off of 0.284. (B) PRAUC-to-prevalence ratio. (C) Calibration plot. Abbreviations: MERWACS, Machineborne Early Renal Warning And Control System; CKD-EPI, Chronic Kidney Disease Epidemiology; ROCAUC, area under the receiver operating characteristic curve; PRAUC, area under the precision-recall curve; Sens., sensitivity; Spec., specificity; Bal. Acc., balanced accuracy; Acc., accuracy.
(DOCX)

S4 Fig User interface and functionality of the MERWACS online application. The figure demonstrates the two primary functions of the open-access MERWACS web application, a tool designed to facilitate self-assessment and support clinical conversations about kidney health screening. (A) Prediction and ‘What-If’ Simulation Interface. This panel shows the main user interface. The specific example shown uses the European Kidney Function Consortium (EKFC)-based model to assess an 86-year-old individual with uncontrolled hypertension (systolic/diastolic blood pressure of 157/96 mmHg) and a weight of 80 kg, resulting in a “Current Predicted Probability” of 45.8%. The “Explore ‘What If’ Scenarios” feature then demonstrates that if this individual were to achieve a target weight of 54 kg and better blood pressure control (138/81 mmHg), the predicted probability drops substantially to 25.5%. The application provides general recommendations based on these probability tiers. (B) Case-Specific Prediction Interpretation (LIME). This panel displays the “How Parameters Impact Probability” tab, which provides model interpretability using a Local Interpretable Model-agnostic Explanations (LIME) plot. The plot visualizes the contribution of each input feature to the final predicted probability. Features with red bars are identified as increasing the probability, while features with blue bars are identified as decreasing it. The length of each bar is proportional to the magnitude of that feature’s influence, offering a transparent explanation of the “why” behind a user’s result. Abbreviation: MERWACS, Machineborne Early Renal Warning And Control System.
(DOCX)

S1 Movie MERWACS application usage. (MP4)

## Abbreviations

CKD: chronic kidney disease
CVD: cardiovascular disease
eGFR: estimated glomerular filtration rate
MERWACS: Machineborne Early Renal Warning And Control System
NHANES: National Health and Nutrition Examination Survey
KNHANES: Korea National Health and Nutrition Examination Survey
NCHS: National Center for Health Statistics
KDCA: Korea Disease Control and Prevention Agency
uACR: urine Albumin-to-Creatinine Ratio
EKFC: European Kidney Function Consortium
CKD-EPI: Chronic Kidney Disease Epidemiology
IQR: Interquartile Range
ANOVA: Analysis Of Variance
BMI: Body Mass Index
PIR: Poverty Income Ratio
SD: standard deviation
XGBoost: extreme gradient boosting
RF: random forest
NNET: model averaged neural network
GLS: generalized least squares
ROCAUC: area under the receiver operating characteristic curve
PRAUC: area under the precision-recall curve
SHAP: SHapley Additive exPlanations
LIME: Local Interpretable Model-agnostic Explanations
DCA: Decision Curve Analysis
