# Supplementary material for: MERWACS: Development and external validation of a non-invasive machine learning tool for identifying subjects to be screened for CKD
Source: PLOS Digit Health. 2026 Jul 9;5(7):e0001486. doi: 10.1371/journal.pdig.0001486 (PMC13349138; doi:10.1371/journal.pdig.0001486)
Supplement: S1 Method — (DOCX) [file pdig.0001486.s001.docx]

**S1 Method. List of a final set of candidate parameters**

**Demographics**

Age in years of the participant at the time of screening

Gender of the participant

In what country {were you/was SP} born?

Education level - Adults 20+

Total number of people in the Household

Age in years of the household reference person at the time of HH screening.

HH reference person's education level

Gender of the household reference person

Marital Status of household reference person

Marital status

Total family income (reported as a range value in dollars)

Poverty income ratio (PIR) - a ratio of family income to poverty threshold

Recode of reported race and Hispanic origin information

**Body Measures**

Arm Circumference (cm)

Upper Arm Length (cm)

Body Mass Index (kg/m**2)

Standing Height (cm)

Upper Leg Length (cm)

Waist Circumference (cm)

Weight (kg)

**Blood Pressure**

Cuff size (cm)

MIL: maximum inflation levels (mm Hg)

60 sec. pulse (30 sec. pulse * 2)

Systolic: Average blood pressure (mm Hg)

Diastolic: Average blood pressure (mm Hg)

**Questionnaire**

Ever been told by a health professional that you had hypertension?

Ever had your blood cholesterol checked?

About how long has it been since {you/SP} last had {your/his/her} blood cholesterol checked?

Ever been told by a doctor or other health professional that your blood cholesterol level was high?

Doctor told you have diabetes

**Food Patterns Equivalents**

Grains defined as whole grains and contain the entire grain kernel â€• the bran, germ, and endosperm (oz. eq.)

Refined grains that do not contain all of the components of the entire grain kernel (oz. eq.)

Dark green vegetables (cup eq.)

Other red and orange vegetables, excluding tomatoes and tomato products (cup eq.)

White potatoes (cup eq.)

Other starchy vegetables, excluding white potatoes (cup eq.)

Tomatoes and tomato products (cup eq.)

Other vegetables not in the vegetable components listed above (cup eq.)

Intact fruits (whole or cut) of citrus, melons, and berries (cup eq.)

Intact fruits (whole or cut); excluding citrus, melons, and berries (cup eq.)

Fluid milk, buttermilk, evaporated milk, dry milk, and calcium fortified soy milk (cup eq.)

Cheeses (cup eq.)

Beef, veal, pork, lamb, and game meat; excludes organ meat and cured meat (oz. eq.)

Frankfurters, sausages, corned beef, cured ham and luncheon meat that are made from beef, pork, or poultry (oz. eq.)

Chicken, turkey, Cornish hens, duck, goose, quail, and pheasant; excludes organ meat and cured meat (oz. eq.)

Seafood high in n-3 fatty acids (oz. eq.)

Seafood low in n-3 fatty acids (oz. eq.)

Eggs and egg substitutes (oz. eq.)

Soy products, excluding calcium fortified soy milk and raw soybeans products (oz. eq.)

Peanuts, tree nuts, and seeds; excludes coconut (oz. eq.)

Beans, peas, and lentils computed as vegetables (cup eq.)

Discretionary oil (grams)

Discretionary solid fat (grams)

Alcoholic beverages and alcohol added to foods after cooking (no. of drinks)

**Medical Conditions**

Ever been told you have asthma

Doctor ever said you had arthritis

Ever told had congestive heart failure

Ever told you had heart attack

Ever told you had a stroke

Ever told you had emphysema

Ever told you had chronic bronchitis

Ever told you had a thyroid problem

Ever told you had cancer or malignancy

Close relative had heart attack?

Close relative had diabetes?

**Total Nutrient Intakes**

Energy (kcal)

Protein (gm)

Carbohydrate (gm)

Total fat (gm)

Cholesterol (mg)

Dietary fiber (gm)

Vitamin A as retinol activity equivalents (mcg)

Thiamin (Vitamin B1) (mg)

Riboflavin (Vitamin B2) (mg)

Niacin (mg)

Vitamin B6 (mg)

Vitamin B12 (mcg)

Vitamin C (mg)

Vitamin E as alpha-tocopherol (mg)

Calcium (mg)

Phosphorus (mg)

Magnesium (mg)

Iron (mg)

Zinc (mg)

Copper (mg)

Sodium (mg)

Potassium (mg)

Selenium (mcg)

Caffeine (mg)

Moisture (gm)

SFA 4:0 (Butanoic) (gm)

SFA 6:0 (Hexanoic) (gm)

SFA 8:0 (Octanoic) (gm)

SFA 10:0 (Decanoic) (gm)

SFA 12:0 (Dodecanoic) (gm)

SFA 14:0 (Tetradecanoic) (gm)

SFA 16:0 (Hexadecanoic) (gm)

SFA 18:0 (Octadecanoic) (gm)

MFA 16:1 (Hexadecenoic) (gm)

MFA 18:1 (Octadecenoic) (gm)

MFA 20:1 (Eicosenoic) (gm)

MFA 22:1 (Docosenoic) (gm)

PFA 18:2 (Octadecadienoic) (gm)

PFA 18:3 (Octadecatrienoic) (gm)

PFA 18:4 (Octadecatetraenoic) (gm)

PFA 20:4 (Eicosatetraenoic) (gm)

PFA 20:5 (Eicosapentaenoic) (gm)

PFA 22:5 (Docosapentaenoic) (gm)

PFA 22:6 (Docosahexaenoic) (gm)

Retinol (mcg)

Beta-carotene (mcg)

**Others**

Serum cotinine (ng/mL)

How much plain water drink in 24 hrs (gm)
