# Supplementary material for: MERWACS: Development and external validation of a non-invasive machine learning tool for identifying subjects to be screened for CKD
Source: PLOS Digit Health. 2026 Jul 9;5(7):e0001486. doi: 10.1371/journal.pdig.0001486 (PMC13349138; doi:10.1371/journal.pdig.0001486)
Supplement: S1 Table — Abbreviation: KNHANES, Korea National Health and Nutrition Examination Survey. Data harmonization notes: The MERWACS model requires predictors that were not uniformly collected in the KNHANES 2021–2023 dataset. *To enable external validation, the following steps were taken: 1) Ethnicity is not collected in KNHANES. To ensure model compatibility, all participants were assigned to the ‘Other Race – Including Multi-Racial’ category. 2) History of congestive heart failure, arm length, and arm circumference, which were missing in some or all KNHANES cycles, were imputed. This was achieved using a random forest imputation model (missForest R package) that was pre-trained on the complete NHANES training dataset; this pre-trained model was then applied to the KNHANES data to predict the missing values, ensuring no outcome information from the KNHANES dataset was used in its own imputation process. 3) The poverty income ratio (PIR) was calculated from household income and national poverty metrics provided by Statistics Korea (KOSTAT). All other variables were directly available. (DOCX) [file pdig.0001486.s002.docx]

**S1 Table. Baseline characteristics of the KNHANES external validation dataset**

|  | **Overall (n=6454)** |
| --- | --- |
| Age (years), Median [25th;75th] | 64.0 [57.0;72.0] |
| Gender female, No. (%) | 3643 (56.4%) |
| Recode of reported race and Hispanic origin information, No. (%)* |  |
| Mexican American | 0 (0.0%) |
| Other Hispanic | 0 (0.0%) |
| Non-Hispanic White | 0 (0.0%) |
| Non-Hispanic Black | 0 (0.0%) |
| Other Race - Including Multi-Racial | 6454 (100.0%) |
| Poverty income ratio (PIR), Median [25th;75th]* | 1.8 [0.9;3.1] |
| Systolic: Average blood pressure (mm Hg), Median [25th;75th] | 123.5 [113.5;133.5] |
| Diastolic: Average blood pressure (mm Hg), Median [25th;75th] | 75.0 [69.0;81.5] |
| 60 sec. pulse (30 sec. pulse * 2), Median [25th;75th] | 70.0 [64.0;76.0] |
| Body Mass Index (kg/m2), Median [25th;75th] | 24.1 [22.0;26.2] |
| Doctor told you have diabetes, No. (%) | 1257 (19.5%) |
| Ever been told by a health professional that you had hypertension?, No. (%) | 2791 (43.2%) |
| Serum creatinine (mg/dL), Median [25th;75th] | 0.8 [0.7;0.9] |
| Urinary albumin (ug/mL), Median [25th;75th] | 7.2 [4.5;13.9] |
| Urinary creatinine (mg/dL), Median [25th;75th] | 97.2 [66.6;139.5] |
| Urinary albumin-to-creatinine ratio (uACR), Median [25th;75th] | 7.2 [4.7;13.8] |
| eGFR (EKFC), Median [25th;75th] | 82.4 [73.1;90.4] |
| eGFR (CKD-EPI 2021), Median [25th;75th] | 94.1 [84.5;100.8] |
| eGFR (CKD-EPI 2009), Median [25th;75th] | 89.5 [79.6;96.8] |
| **Outcome of the study, No. (%)** |  |
| Reduced Kidney Health (eGFR EKFC) | 1109 (17.2%) |
| Reduced Kidney Health (eGFR CKD-EPI2021) | 916 (14.2%) |
| Reduced Kidney Health (eGFR CKD-EPI 2009) | 995 (15.4%) |

Abbreviation: KNHANES, Korea National Health and Nutrition Examination Survey. Data harmonization notes: The MERWACS model requires predictors that were not uniformly collected in the KNHANES 2021–2023 dataset. *To enable external validation, the following steps were taken: 1) Ethnicity is not collected in KNHANES. To ensure model compatibility, all participants were assigned to the ‘Other Race – Including Multi-Racial’ category. 2) History of congestive heart failure, arm length, and arm circumference, which were missing in some or all KNHANES cycles, were imputed. This was achieved using a random forest imputation model (missForest R package) that was pre-trained on the complete NHANES training dataset this pre-trained model was then applied to the KNHANES data to predict the missing values, ensuring no outcome information from the KNHANES dataset was used in its own imputation process. 3) The poverty income ratio (PIR) was calculated from household income and national poverty metrics provided by Statistics Korea (KOSTAT). All other variables were directly available.
