# Supplementary material for: MERWACS: Development and external validation of a non-invasive machine learning tool for identifying subjects to be screened for CKD
Source: PLOS Digit Health. 2026 Jul 9;5(7):e0001486. doi: 10.1371/journal.pdig.0001486 (PMC13349138; doi:10.1371/journal.pdig.0001486)
Supplement: S2 Table — The table presents the number and percentage of participants classified into four mutually exclusive groups based on the two components of the composite outcome: low eGFR (below the age- and sex-specific 2.5th percentile from Eriksen et al., 2020) and elevated uACR (urine albumin-to-creatinine ratio ≥ 30 mg/g). The composite row corresponds to the primary outcome used in the manuscript (one or both criteria met) and serves as an internal consistency check against the prevalence figures reported in Table 1. Abbreviations: CKD-EPI, Chronic Kidney Disease Epidemiology Collaboration; EKFC, European Kidney Function Consortium; KNHANES, Korea National Health and Nutrition Examination Survey; NHANES, National Health and Nutrition Examination Survey; uACR, urine albumin-to-creatinine ratio. (DOCX) [file pdig.0001486.s003.docx]

**S2 Table. Decomposition of the composite reduced kidney function outcome by individual criterion, for NHANES and KNHANES datasets across three eGFR equations**

The table presents the number and percentage of participants classified into four mutually exclusive groups based on the two components of the composite outcome: low eGFR (below the age- and sex-specific 2.5^th^ percentile from Eriksen et al., 2020) and elevated uACR (urine albumin-to-creatinine ratio ≥ 30 mg/g). The composite row corresponds to the primary outcome used in the manuscript (one or both criteria met) and serves as an internal consistency check against the prevalence figures reported in Table 1.

| **Outcome component** | **NHANES (n=13,619)** | | | **KNHANES (n=6,454)** | | |
| --- | --- | --- | --- | --- | --- | --- |
|  | **EKFC** | **CKD-EPI 2021** | **CKD-EPI 2009** | **EKFC** | **CKD-EPI 2021** | **CKD-EPI 2009** |
| Neither | 8805 (64.7%) | 10069 (73.9%) | 9798 (71.9%) | 5345 (82.8%) | 5538 (85.8%) | 5459 (84.6%) |
| Low eGFR only | 2585 (19%) | 1321 (9.7%) | 1592 (11.7%) | 318 (4.9%) | 125 (1.9%) | 204 (3.2%) |
| High uACR only | 1417 (10.4%) | 1665 (12.2%) | 1611 (11.8%) | 605 (9.4%) | 664 (10.3%) | 636 (9.9%) |
| Both | 812 (6%) | 564 (4.1%) | 618 (4.5%) | 186 (2.9%) | 127 (2%) | 155 (2.4%) |
| **Composite (any criterion)** | 4814 (35.3%) | 3550 (26.1%) | 3821 (28.1%) | 1109 (17.2%) | 916 (14.2%) | 995 (15.4%) |

Abbreviations: CKD-EPI, Chronic Kidney Disease Epidemiology Collaboration; EKFC, European Kidney Function Consortium; KNHANES, Korea National Health and Nutrition Examination Survey; NHANES, National Health and Nutrition Examination Survey; uACR, urine albumin-to-creatinine ratio.
