# Supplementary material for: MERWACS: Development and external validation of a non-invasive machine learning tool for identifying subjects to be screened for CKD
Source: PLOS Digit Health. 2026 Jul 9;5(7):e0001486. doi: 10.1371/journal.pdig.0001486 (PMC13349138; doi:10.1371/journal.pdig.0001486)
Supplement: S3 Table — Abbreviations: ML, machine learning; EKFC, European Kidney Function Consortium; ROCAUC, area under the receiver operating characteristic curve; PRAUC, area under the precision-recall curve; SD, standard deviation. (DOCX) [file pdig.0001486.s004.docx]

**S3 Table. Cross-validation results of the three ML algorithms (EKFC formula)**

| **Resample** | **Random Forest** | | | **Model Averaged Neural Network** | | | **Extreme Gradient Boosting** | | |
| --- | --- | --- | --- | --- | --- | --- | --- | --- | --- |
|  | **ROCAUC** | **PRAUC** | **Brier** | **ROCAUC** | **PRAUC** | **Brier** | **ROCAUC** | **PRAUC** | **Brier** |
| Rep1.Fold01 | 0.716 | 0.577 | 0.211 | 0.716 | 0.567 | 0.199 | 0.726 | 0.588 | 0.197 |
| Rep1.Fold02 | 0.649 | 0.496 | 0.232 | 0.672 | 0.524 | 0.210 | 0.670 | 0.511 | 0.210 |
| Rep1.Fold03 | 0.693 | 0.562 | 0.214 | 0.707 | 0.559 | 0.201 | 0.710 | 0.554 | 0.201 |
| Rep1.Fold04 | 0.695 | 0.565 | 0.216 | 0.706 | 0.569 | 0.201 | 0.712 | 0.572 | 0.201 |
| Rep1.Fold05 | 0.690 | 0.581 | 0.213 | 0.711 | 0.586 | 0.198 | 0.705 | 0.605 | 0.199 |
| Rep1.Fold06 | 0.664 | 0.536 | 0.222 | 0.665 | 0.532 | 0.211 | 0.670 | 0.538 | 0.209 |
| Rep1.Fold07 | 0.705 | 0.554 | 0.218 | 0.714 | 0.579 | 0.199 | 0.716 | 0.570 | 0.200 |
| Rep1.Fold08 | 0.689 | 0.560 | 0.217 | 0.702 | 0.575 | 0.201 | 0.703 | 0.572 | 0.202 |
| Rep1.Fold09 | 0.688 | 0.568 | 0.215 | 0.703 | 0.585 | 0.200 | 0.704 | 0.589 | 0.200 |
| Rep1.Fold10 | 0.680 | 0.535 | 0.221 | 0.687 | 0.535 | 0.206 | 0.692 | 0.553 | 0.204 |
| Rep2.Fold01 | 0.698 | 0.551 | 0.217 | 0.709 | 0.565 | 0.200 | 0.710 | 0.572 | 0.201 |
| Rep2.Fold02 | 0.701 | 0.571 | 0.214 | 0.708 | 0.577 | 0.200 | 0.714 | 0.587 | 0.199 |
| Rep2.Fold03 | 0.665 | 0.529 | 0.225 | 0.670 | 0.527 | 0.210 | 0.681 | 0.546 | 0.206 |
| Rep2.Fold04 | 0.661 | 0.544 | 0.223 | 0.687 | 0.547 | 0.205 | 0.689 | 0.558 | 0.204 |
| Rep2.Fold05 | 0.693 | 0.558 | 0.217 | 0.708 | 0.575 | 0.200 | 0.707 | 0.574 | 0.201 |
| Rep2.Fold06 | 0.685 | 0.559 | 0.216 | 0.694 | 0.570 | 0.203 | 0.695 | 0.563 | 0.203 |
| Rep2.Fold07 | 0.677 | 0.551 | 0.217 | 0.701 | 0.573 | 0.201 | 0.693 | 0.562 | 0.204 |
| Rep2.Fold08 | 0.713 | 0.582 | 0.210 | 0.721 | 0.585 | 0.197 | 0.722 | 0.583 | 0.198 |
| Rep2.Fold09 | 0.684 | 0.549 | 0.220 | 0.695 | 0.561 | 0.203 | 0.690 | 0.573 | 0.204 |
| Rep2.Fold10 | 0.677 | 0.552 | 0.220 | 0.692 | 0.560 | 0.204 | 0.689 | 0.555 | 0.205 |
| Rep3.Fold01 | 0.675 | 0.544 | 0.221 | 0.691 | 0.557 | 0.204 | 0.691 | 0.560 | 0.204 |
| Rep3.Fold02 | 0.697 | 0.559 | 0.213 | 0.709 | 0.572 | 0.200 | 0.713 | 0.571 | 0.201 |
| Rep3.Fold03 | 0.690 | 0.586 | 0.215 | 0.689 | 0.579 | 0.203 | 0.693 | 0.585 | 0.202 |
| Rep3.Fold04 | 0.652 | 0.519 | 0.227 | 0.658 | 0.521 | 0.213 | 0.654 | 0.521 | 0.212 |
| Rep3.Fold05 | 0.688 | 0.552 | 0.221 | 0.709 | 0.557 | 0.201 | 0.709 | 0.564 | 0.202 |
| Rep3.Fold06 | 0.679 | 0.539 | 0.220 | 0.684 | 0.542 | 0.206 | 0.689 | 0.562 | 0.204 |
| Rep3.Fold07 | 0.681 | 0.538 | 0.220 | 0.699 | 0.556 | 0.202 | 0.705 | 0.560 | 0.202 |
| Rep3.Fold08 | 0.717 | 0.615 | 0.204 | 0.731 | 0.628 | 0.192 | 0.725 | 0.613 | 0.195 |
| Rep3.Fold09 | 0.698 | 0.557 | 0.215 | 0.717 | 0.567 | 0.199 | 0.715 | 0.567 | 0.200 |
| Rep3.Fold10 | 0.694 | 0.551 | 0.218 | 0.702 | 0.568 | 0.202 | 0.710 | 0.580 | 0.200 |
| **Mean (SD)** | 0.686  (0.0170) | 0.555  (0.0221) | 0.218  (0.00538) | 0.699  (0.0169) | 0.563  (0.0221) | 0.202  (0.00444) | 0.700  (0.0169) | 0.567  (0.0213) | 0.202  (0.00366) |

Abbreviations: ML, machine learning; EKFC, European Kidney Function Consortium; ROCAUC, area under the receiver operating characteristic curve; PRAUC, area under the precision-recall curve; SD, standard deviation.
