# Supplementary material for: MERWACS: Development and external validation of a non-invasive machine learning tool for identifying subjects to be screened for CKD
Source: PLOS Digit Health. 2026 Jul 9;5(7):e0001486. doi: 10.1371/journal.pdig.0001486 (PMC13349138; doi:10.1371/journal.pdig.0001486)
Supplement: S4 Table — Abbreviations: ML, machine learning; CKD-EPI, Chronic Kidney Disease Epidemiology; ROCAUC, area under the receiver operating characteristic curve; PRAUC, area under the precision-recall curve; SD, standard deviation. (DOCX) [file pdig.0001486.s005.docx]

**S4 Table. Cross-validation results of the three ML algorithms (CKD-EPI 2021 formula)**

| **Resample** | **Random Forest** | | | **Model Averaged Neural Network** | | | **Extreme Gradient Boosting** | | |
| --- | --- | --- | --- | --- | --- | --- | --- | --- | --- |
|  | **ROCAUC** | **PRAUC** | **Brier** | **ROCAUC** | **PRAUC** | **Brier** | **ROCAUC** | **PRAUC** | **Brier** |
| Rep1.Fold01 | 0.697 | 0.450 | 0.181 | 0.693 | 0.460 | 0.174 | 0.701 | 0.461 | 0.173 |
| Rep1.Fold02 | 0.692 | 0.460 | 0.180 | 0.703 | 0.466 | 0.173 | 0.699 | 0.455 | 0.173 |
| Rep1.Fold03 | 0.723 | 0.502 | 0.174 | 0.735 | 0.520 | 0.164 | 0.740 | 0.513 | 0.165 |
| Rep1.Fold04 | 0.690 | 0.475 | 0.181 | 0.713 | 0.499 | 0.169 | 0.710 | 0.480 | 0.170 |
| Rep1.Fold05 | 0.706 | 0.476 | 0.178 | 0.718 | 0.486 | 0.169 | 0.724 | 0.487 | 0.168 |
| Rep1.Fold06 | 0.721 | 0.512 | 0.173 | 0.717 | 0.496 | 0.168 | 0.722 | 0.512 | 0.167 |
| Rep1.Fold07 | 0.708 | 0.483 | 0.177 | 0.740 | 0.519 | 0.163 | 0.739 | 0.520 | 0.165 |
| Rep1.Fold08 | 0.741 | 0.566 | 0.168 | 0.744 | 0.556 | 0.161 | 0.750 | 0.562 | 0.160 |
| Rep1.Fold09 | 0.668 | 0.434 | 0.186 | 0.688 | 0.442 | 0.175 | 0.694 | 0.446 | 0.174 |
| Rep1.Fold10 | 0.711 | 0.494 | 0.176 | 0.731 | 0.505 | 0.166 | 0.731 | 0.502 | 0.167 |
| Rep2.Fold01 | 0.701 | 0.497 | 0.177 | 0.707 | 0.486 | 0.171 | 0.717 | 0.503 | 0.168 |
| Rep2.Fold02 | 0.691 | 0.470 | 0.180 | 0.709 | 0.470 | 0.171 | 0.712 | 0.474 | 0.171 |
| Rep2.Fold03 | 0.713 | 0.504 | 0.173 | 0.720 | 0.506 | 0.168 | 0.723 | 0.513 | 0.166 |
| Rep2.Fold04 | 0.661 | 0.429 | 0.187 | 0.676 | 0.431 | 0.178 | 0.685 | 0.437 | 0.176 |
| Rep2.Fold05 | 0.705 | 0.481 | 0.177 | 0.725 | 0.506 | 0.167 | 0.725 | 0.496 | 0.167 |
| Rep2.Fold06 | 0.704 | 0.475 | 0.180 | 0.712 | 0.471 | 0.170 | 0.724 | 0.500 | 0.168 |
| Rep2.Fold07 | 0.687 | 0.444 | 0.182 | 0.696 | 0.465 | 0.173 | 0.697 | 0.456 | 0.173 |
| Rep2.Fold08 | 0.739 | 0.531 | 0.172 | 0.754 | 0.563 | 0.161 | 0.744 | 0.538 | 0.164 |
| Rep2.Fold09 | 0.712 | 0.494 | 0.176 | 0.730 | 0.514 | 0.166 | 0.734 | 0.517 | 0.165 |
| Rep2.Fold10 | 0.745 | 0.527 | 0.172 | 0.740 | 0.512 | 0.165 | 0.745 | 0.530 | 0.164 |
| Rep3.Fold01 | 0.709 | 0.494 | 0.177 | 0.709 | 0.491 | 0.169 | 0.710 | 0.501 | 0.168 |
| Rep3.Fold02 | 0.721 | 0.491 | 0.175 | 0.726 | 0.498 | 0.168 | 0.730 | 0.502 | 0.167 |
| Rep3.Fold03 | 0.696 | 0.481 | 0.178 | 0.713 | 0.483 | 0.169 | 0.714 | 0.489 | 0.169 |
| Rep3.Fold04 | 0.715 | 0.519 | 0.175 | 0.740 | 0.540 | 0.163 | 0.744 | 0.542 | 0.163 |
| Rep3.Fold05 | 0.680 | 0.441 | 0.183 | 0.691 | 0.453 | 0.176 | 0.691 | 0.451 | 0.175 |
| Rep3.Fold06 | 0.687 | 0.464 | 0.183 | 0.701 | 0.478 | 0.172 | 0.703 | 0.475 | 0.172 |
| Rep3.Fold07 | 0.706 | 0.500 | 0.176 | 0.710 | 0.500 | 0.169 | 0.717 | 0.517 | 0.168 |
| Rep3.Fold08 | 0.697 | 0.470 | 0.179 | 0.710 | 0.473 | 0.171 | 0.718 | 0.479 | 0.170 |
| Rep3.Fold09 | 0.727 | 0.487 | 0.176 | 0.740 | 0.510 | 0.164 | 0.744 | 0.509 | 0.165 |
| Rep3.Fold10 | 0.709 | 0.478 | 0.176 | 0.727 | 0.503 | 0.167 | 0.729 | 0.498 | 0.167 |
| **Mean (SD)** | 0.705 (0.0195) | 0.484 (0.0300) | 0.178 (0.00422) | 0.717 (0.0188) | 0.493 (0.0304) | 0.169 (0.00430) | 0.721 (0.0179) | 0.496 (0.0300) | 0.168 (0.00381) |

Abbreviations: ML, machine learning; CKD-EPI, Chronic Kidney Disease Epidemiology; ROCAUC, area under the receiver operating characteristic curve; PRAUC, area under the precision-recall curve; SD, standard deviation.
