# Supplementary material for: MERWACS: Development and external validation of a non-invasive machine learning tool for identifying subjects to be screened for CKD
Source: PLOS Digit Health. 2026 Jul 9;5(7):e0001486. doi: 10.1371/journal.pdig.0001486 (PMC13349138; doi:10.1371/journal.pdig.0001486)
Supplement: S5 Table — Abbreviations: ML, machine learning; CKD-EPI, Chronic Kidney Disease Epidemiology; ROCAUC, area under the receiver operating characteristic curve; PRAUC, area under the precision-recall curve; SD, standard deviation. (DOCX) [file pdig.0001486.s006.docx]

**S5 Table. Cross-validation results of the three ML algorithms (CKD-EPI 2009 formula)**

| **Resample** | **Random Forest** | | | **Model Averaged Neural Network** | | | **Extreme Gradient Boosting** | | |
| --- | --- | --- | --- | --- | --- | --- | --- | --- | --- |
|  | **ROCAUC** | **PRAUC** | **Brier** | **ROCAUC** | **PRAUC** | **Brier** | **ROCAUC** | **PRAUC** | **Brier** |
| Rep1.Fold01 | 0.697 | 0.489 | 0.184 | 0.698 | 0.493 | 0.179 | 0.693 | 0.488 | 0.181 |
| Rep1.Fold02 | 0.667 | 0.468 | 0.189 | 0.677 | 0.466 | 0.183 | 0.679 | 0.475 | 0.183 |
| Rep1.Fold03 | 0.669 | 0.461 | 0.190 | 0.680 | 0.483 | 0.182 | 0.679 | 0.477 | 0.183 |
| Rep1.Fold04 | 0.685 | 0.507 | 0.184 | 0.689 | 0.510 | 0.178 | 0.691 | 0.510 | 0.180 |
| Rep1.Fold05 | 0.691 | 0.444 | 0.189 | 0.710 | 0.479 | 0.179 | 0.712 | 0.478 | 0.180 |
| Rep1.Fold06 | 0.695 | 0.481 | 0.183 | 0.713 | 0.491 | 0.177 | 0.717 | 0.496 | 0.179 |
| Rep1.Fold07 | 0.659 | 0.449 | 0.193 | 0.697 | 0.481 | 0.181 | 0.691 | 0.462 | 0.183 |
| Rep1.Fold08 | 0.657 | 0.413 | 0.196 | 0.675 | 0.438 | 0.187 | 0.676 | 0.440 | 0.186 |
| Rep1.Fold09 | 0.710 | 0.506 | 0.179 | 0.716 | 0.531 | 0.174 | 0.715 | 0.528 | 0.177 |
| Rep1.Fold10 | 0.678 | 0.496 | 0.186 | 0.690 | 0.499 | 0.180 | 0.700 | 0.517 | 0.179 |
| Rep2.Fold01 | 0.676 | 0.500 | 0.186 | 0.696 | 0.503 | 0.179 | 0.705 | 0.509 | 0.179 |
| Rep2.Fold02 | 0.674 | 0.460 | 0.190 | 0.680 | 0.481 | 0.182 | 0.677 | 0.470 | 0.184 |
| Rep2.Fold03 | 0.650 | 0.455 | 0.192 | 0.689 | 0.475 | 0.182 | 0.685 | 0.474 | 0.183 |
| Rep2.Fold04 | 0.684 | 0.488 | 0.185 | 0.693 | 0.500 | 0.179 | 0.689 | 0.500 | 0.181 |
| Rep2.Fold05 | 0.667 | 0.447 | 0.190 | 0.683 | 0.476 | 0.182 | 0.688 | 0.484 | 0.182 |
| Rep2.Fold06 | 0.666 | 0.442 | 0.193 | 0.690 | 0.465 | 0.183 | 0.687 | 0.463 | 0.184 |
| Rep2.Fold07 | 0.680 | 0.472 | 0.189 | 0.692 | 0.477 | 0.181 | 0.695 | 0.484 | 0.181 |
| Rep2.Fold08 | 0.674 | 0.459 | 0.189 | 0.699 | 0.477 | 0.181 | 0.698 | 0.469 | 0.182 |
| Rep2.Fold09 | 0.692 | 0.463 | 0.186 | 0.693 | 0.489 | 0.180 | 0.704 | 0.493 | 0.180 |
| Rep2.Fold10 | 0.714 | 0.526 | 0.178 | 0.729 | 0.529 | 0.173 | 0.725 | 0.545 | 0.175 |
| Rep3.Fold01 | 0.654 | 0.433 | 0.194 | 0.687 | 0.468 | 0.182 | 0.694 | 0.469 | 0.182 |
| Rep3.Fold02 | 0.675 | 0.465 | 0.189 | 0.696 | 0.470 | 0.181 | 0.690 | 0.464 | 0.183 |
| Rep3.Fold03 | 0.683 | 0.500 | 0.184 | 0.683 | 0.504 | 0.180 | 0.683 | 0.501 | 0.181 |
| Rep3.Fold04 | 0.698 | 0.470 | 0.186 | 0.696 | 0.479 | 0.181 | 0.703 | 0.479 | 0.181 |
| Rep3.Fold05 | 0.651 | 0.436 | 0.195 | 0.660 | 0.434 | 0.190 | 0.672 | 0.460 | 0.185 |
| Rep3.Fold06 | 0.710 | 0.511 | 0.181 | 0.719 | 0.540 | 0.173 | 0.718 | 0.530 | 0.177 |
| Rep3.Fold07 | 0.683 | 0.500 | 0.183 | 0.709 | 0.495 | 0.177 | 0.710 | 0.498 | 0.178 |
| Rep3.Fold08 | 0.670 | 0.440 | 0.192 | 0.686 | 0.465 | 0.182 | 0.682 | 0.468 | 0.183 |
| Rep3.Fold09 | 0.695 | 0.470 | 0.186 | 0.697 | 0.484 | 0.180 | 0.698 | 0.493 | 0.180 |
| Rep3.Fold10 | 0.689 | 0.480 | 0.185 | 0.705 | 0.529 | 0.176 | 0.711 | 0.531 | 0.178 |
| **Mean (SD)** | 0.680 (0.0173) | 0.471 (0.0272) | 0.188 (0.00458) | 0.694 (0.0144) | 0.487 (0.0247) | 0.180 (0.00358) | 0.696 (0.0140) | 0.488 (0.0248) | 0.181 (0.00255) |

Abbreviations: ML, machine learning; CKD-EPI, Chronic Kidney Disease Epidemiology; ROCAUC, area under the receiver operating characteristic curve; PRAUC, area under the precision-recall curve; SD, standard deviation.
