# Supplementary material for: MERWACS: Development and external validation of a non-invasive machine learning tool for identifying subjects to be screened for CKD
Source: PLOS Digit Health. 2026 Jul 9;5(7):e0001486. doi: 10.1371/journal.pdig.0001486 (PMC13349138; doi:10.1371/journal.pdig.0001486)
Supplement: S6 Table — Abbreviations: ML, machine learning; eGFR, estimated glomerular filtration rate; EKFC, European Kidney Function Consortium; CKD-EPI, Chronic Kidney Disease Epidemiology. (DOCX) [file pdig.0001486.s007.docx]

**S6 Table. Hyperparameters tuning and results for the three ML algorithms**

| **Outcome eGFR formula** | **Machine learning algorithms** | **Hyperparameters** |
| --- | --- | --- |
| **EKFC** | Random Forest | mtry=2 |
|  | Model averaged neural network | size=10  decay= 1.196296  bag=FALSE |
|  | Extreme Gradient Boosting | nrounds=129  max_depth=3  eta= 0.03829923  gamma= 3.568553  colsample_bytree= 0.38597  min_child_weight=2  subsample= 0.7259456 |
| **CKD-EPI 2021** | Random Forest | mtry=2 |
|  | Model averaged neural network | size=10  decay= 1.544789  bag=FALSE |
|  | Extreme Gradient Boosting | nrounds=231  max_depth=3  eta= 0.02062629  gamma= 3.34773  colsample_bytree= 0.6964735  min_child_weight=3  subsample= 0.4887014 |
| **CKD-EPI 2009** | Random Forest | mtry=2 |
|  | Model averaged neural network | size=6  decay= 0.1734185  bag=FALSE |
|  | Extreme Gradient Boosting | nrounds=406  max_depth=2  eta= 0.01092244  gamma= 3.249494  colsample_bytree= 0.4377271  min_child_weight=12  subsample= 0.8383812 |

Abbreviations: ML, machine learning; eGFR, estimated glomerular filtration rate; EKFC, European Kidney Function Consortium; CKD-EPI, Chronic Kidney Disease Epidemiology.
