# Supplementary material for: MERWACS: Development and external validation of a non-invasive machine learning tool for identifying subjects to be screened for CKD
Source: PLOS Digit Health. 2026 Jul 9;5(7):e0001486. doi: 10.1371/journal.pdig.0001486 (PMC13349138; doi:10.1371/journal.pdig.0001486)
Supplement: S7 Table — Calibration intercept and slope for the MERWACS model computed for the internal test set (NHANES 1988–2018; n = 4,085) and external validation dataset (KNHANES 2021–2023; n = 6,454), overall and within each 5-year age stratum, for all three eGFR equation-based models. Values are presented as estimate (95% confidence interval). Estimates for the 85–89 and 90+ strata in the internal dataset should be interpreted with caution due to small sample sizes. In the 90+ stratum, the upper confidence interval for the CKD-EPI 2009 calibration slope could not be estimated because the sparse data prevented the profile likelihood from converging to an upper bound. No external estimates are available for these age groups as KNHANES participants were aged up to 80 years. Abbreviations: EKFC, European Kidney Function Consortium; CKD-EPI, Chronic Kidney Disease Epidemiology Collaboration; NHANES, National Health and Nutrition Examination Survey; KNHANES, Korea National Health and Nutrition Examination Survey. (DOCX) [file pdig.0001486.s008.docx]

**S7 Table. Calibration-in-the-large metrics for MERWACS across three eGFR equations, overall and stratified by age group, for internal and external validation datasets**

Calibration intercept and slope for the MERWACS model computed for the internal test set (NHANES 1988–2018; n=4,085) and external validation dataset (KNHANES 2021–2023; n=6,454), overall and within each 5-year age stratum, for all three eGFR equation-based models. Values are presented as estimate (95% confidence interval).

| **Dataset** | **Age**  **group** | **EKFC** | | **CKD-EPI 2021** | | **CKD-EPI 2009** | |
| --- | --- | --- | --- | --- | --- | --- | --- |
|  |  | **Intercept** | **Slope** | **Intercept** | **Slope** | **Intercept** | **Slope** |
| **Internal** | 50-54 | 0.0484 (-0.135;0.227) | 0.789 (0.535;1.05) | 0.177 (-0.0302;0.377) | 0.796 (0.561;1.04) | 0.0872 (-0.108;0.276) | 0.849 (0.551;1.16) |
|  | 55-59 | -0.0611 (-0.262;0.135) | 0.491 (0.248;0.742) | -0.149 (-0.387;0.0787) | 0.195 (0.0320;0.397) | -0.0810 (-0.300;0.130) | 0.551 (0.239;0.867) |
|  | 60-64 | -0.0718 (-0.241;0.0939) | 0.750 (0.553;0.957) | -0.0374 (-0.223;0.144) | 0.745 (0.558;0.939) | 0.00209 (-0.175;0.175) | 1.01 (0.768;1.26) |
|  | 65-69 | 0.0752 (-0.100;0.248) | 0.882 (0.665;1.11) | 0.00830 (-0.188;0.200) | 0.708 (0.508;0.916) | 0.124 (-0.0584;0.303) | 0.928 (0.688;1.18) |
|  | 70-74 | -0.00720 (-0.184;0.167) | 0.673 (0.448;0.908) | 0.0908 (-0.100;0.277) | 0.547 (0.339;0.761) | 0.0131 (-0.172;0.194) | 0.703 (0.459;0.954) |
|  | 75-79 | -0.0539 (-0.278;0.169) | 0.520 (0.254;0.812) | -0.0928 (-0.335;0.143) | 0.573 (0.287;0.870) | -0.0607 (-0.294;0.168) | 0.719 (0.409;1.04) |
|  | 80-84 | -0.0582 (-0.262;0.145) | 0.783 (0.488;1.11) | -0.0715 (-0.288;0.141) | 0.646 (0.377;0.927) | -0.0325 (-0.243;0.175) | 0.687 (0.413;0.975) |
|  | 85-89 | 0.0707 (-0.363;0.507) | 0.339 (-0.197;0.980) | -0.0688 (-0.530;0.377) | 0.0744 (-0.124;0.322) | 0.131 (-0.310;0.565) | -0.0374 (-0.422;0.245) |
|  | 90+ | 0.625 (-0.449;1.72) | 2.52 (0.438;5.59) | 0.542 (-0.581;1.60) | 7.04 (2.07;17.7) | 0.371 (-0.752;1.43) | 13.1 (3.55; NA) |
|  | **Overall** | -0.00990 (-0.0795;0.0593) | 0.754 (0.673;0.838) | -0.00162 (-0.0783;0.0743) | 0.621 (0.543;0.700) | 0.0201 (-0.0528;0.0925) | 0.800 (0.709;0.892) |
| **External** | 50-54 | -0.560 (-0.772;-0.358) | 0.720 (0.410;1.04) | -0.185 (-0.414;0.0317) | 0.540 (0.247;0.872) | -0.533 (-0.749;-0.328) | 1.23 (0.891;1.59) |
|  | 55-59 | -0.288 (-0.477;-0.106) | 0.891 (0.600;1.20) | -0.102 (-0.309;0.0953) | 0.910 (0.606;1.22) | -0.338 (-0.535;-0.149) | 1.08 (0.774;1.40) |
|  | 60-64 | -0.445 (-0.621;-0.275) | 0.978 (0.705;1.26) | -0.353 (-0.550;-0.165) | 1.14 (0.859;1.42) | -0.491 (-0.677;-0.312) | 0.968 (0.702;1.24) |
|  | 65-69 | -0.367 (-0.530;-0.209) | 1.00 (0.738;1.28) | -0.140 (-0.314;0.0273) | 1.18 (0.916;1.45) | -0.329 (-0.497;-0.167) | 1.12 (0.847;1.40) |
|  | 70-74 | -0.380 (-0.554;-0.211) | 1.22 (0.908;1.54) | -0.208 (-0.395;-0.0279) | 1.10 (0.815;1.39) | -0.384 (-0.567;-0.207) | 1.13 (0.823;1.45) |
|  | 75-79 | -0.564 (-0.741;-0.391) | 1.17 (0.836;1.52) | -0.429 (-0.623;-0.242) | 1.21 (0.886;1.56) | -0.515 (-0.702;-0.334) | 1.15 (0.839;1.49) |
|  | 80-84 | -0.506 (-0.695;-0.322) | 0.950 (0.594;1.33) | -0.455 (-0.660;-0.257) | 0.864 (0.514;1.23) | -0.482 (-0.682;-0.288) | 0.922 (0.583;1.27) |
|  | **Overall** | -0.441 (-0.509;-0.374) | 0.957 (0.865;1.05) | -0.270 (-0.344;-0.198) | 0.940 (0.847;1.03) | -0.434 (-0.505;-0.364) | 1.04 (0.946;1.15) |

Estimates for the 85–89 and 90+ strata in the internal dataset should be interpreted with caution due to small sample sizes. In the 90+ stratum, the upper confidence interval for the CKD-EPI 2009 calibration slope could not be estimated because the sparse data prevented the profile likelihood from converging to an upper bound. No external estimates are available for these age groups as KNHANES participants were aged up to 80 years. Abbreviations: EKFC, European Kidney Function Consortium; CKD-EPI, Chronic Kidney Disease Epidemiology Collaboration; NHANES, National Health and Nutrition Examination Survey; KNHANES, Korea National Health and Nutrition Examination Survey.
