# Supplementary material for: MERWACS: Development and external validation of a non-invasive machine learning tool for identifying subjects to be screened for CKD
Source: PLOS Digit Health. 2026 Jul 9;5(7):e0001486. doi: 10.1371/journal.pdig.0001486 (PMC13349138; doi:10.1371/journal.pdig.0001486)
Supplement: S8 Table — Abbreviation: MERWACS, Machineborne Early Renal Warning And Control System; ROCAUC, area under the receiver operating characteristic curve; PRAUC, area under the precision-recall curve. (DOCX) [file pdig.0001486.s009.docx]

**S8 Table. Internal validation of MERWACS across various subpopulations and scenarios**

| **Subpopulations** | | **Number** | **ROCAUC**  **X 100** | **PRAUC**  **X 100** | **PRAUC/Prevalence ratio** | **1-Brier Score**  **(%)** | **Sensitivity**  **(%)** | **Specificity**  **(%)** | **Balanced Accuracy**  **(%)** | **Accuracy (%)** |
| --- | --- | --- | --- | --- | --- | --- | --- | --- | --- | --- |
| **Age** | 50-54 | 670 | 65.9  (61.3-70.5) | 42.8  (34.9-50.0) | 1.64  (1.41-1.90) | 82.0  (80.4-83.4) | 38.3  (30.6-45.3) | 83.5  (80.0-86.6) | 60.9  (57.1-64.7) | 71.7  (68.4-74.8) |
|  | 55-59 | 564 | 59.5  (54.1-65.1) | 36.5  (29.1-43.9) | 1.39  (1.17-1.65) | 80.9  (79.2-82.6) | 37.7  (30.1-45.8) | 76.6  (72.4-80.7) | 57.2  (52.9-61.9) | 66.4  (62.4-70.4) |
|  | 60-64 | 741 | 68.8  (64.6-73.0) | 49.1  (42.6-56.1) | 1.58  (1.41-1.78) | 80.5  (79.2-81.7) | 59.4  (53.5-65.7) | 69.1  (65.2-73.1) | 64.3  (60.7-68.1) | 66.1  (62.9-69.5) |
|  | 65-69 | 637 | 70.7  (66.7-74.9) | 60.4  (53.6-67.0) | 1.64  (1.48-1.82) | 79.6  (78.4-81.0) | 68.9  (62.9-74.8) | 62.8  (58.2-67.5) | 65.9  (62.1-69.7) | 65.1  (61.2-68.8) |
|  | 70-74 | 604 | 65.1  (60.4-69.5) | 53.2  (46.4-59.8) | 1.42  (1.27-1.58) | 78.2  (76.8-79.5) | 67.9  (61.5-73.9) | 50.4  (45.4-55.5) | 59.1  (55.2-63.1) | 57.0  (53.1-60.8) |
|  | 75-79 | 350 | 63.1  (56.8-69.2) | 60.3  (52.5-68.2) | 1.30  (1.16-1.46) | 76.5  (75.1-77.9) | 82.2  (76.3-88.0) | 24.0  (17.9-30.5) | 53.1  (48.8-57.4) | 51.0  (46.0-56.3) |
|  | 80-84 | 414 | 66.9  (61.9-71.9) | 66.1  (59.7-72.9) | 1.32  (1.20-1.44) | 76.9  (75.9-78.0) | 92.7  (89.1-96.1) | 17.1  (11.6-22.2) | 54.9  (51.8-58.2) | 55.1  (50.2-60.1) |
|  | 85-89 | 90 | 60.3  (48.3-71.3) | 64.2  (49.0-78.8) | 1.21  (0.994-1.46) | 75.7  (73.2-77.9) | 93.8  (86.3-100.) | 4.78  (0-12.5) | 49.3  (44.7-53.6) | 52.2  (42.2-62.2) |
|  | $\geq$90 | 15 | 86.1  (62.5-100.) | 89.8  (66.5-100.) | 1.79  (1.20-3.00) | 78.8  (71.0-85.8) | 75.5  (42.9-100.) | 70.9  (33.3-100.) | 73.2  (48.2-94.4) | 73.4  (46.7-93.3) |
| **Gender** | Female | 2079 | 67.7  (65.2-69.9) | 53.1  (49.3-56.8) | 1.54  (1.44-1.64) | 79.5  (78.7-80.2) | 62.9  (59.4-66.0) | 63.3  (60.8-65.9) | 63.1  (60.9-65.2) | 63.2  (61.1-65.2) |
|  | Male | 2006 | 70.1  (67.7-72.5) | 56.9  (53.1-60.9) | 1.57  (1.48-1.67) | 79.5  (78.8-80.3) | 69.2  (65.9-72.4) | 58.7  (56.1-61.2) | 64.0  (61.9-66.1) | 62.5  (60.4-64.7) |

Abbreviation: MERWACS, Machineborne Early Renal Warning And Control System; ROCAUC, area under the receiver operating characteristic curve; PRAUC, area under the precision-recall curve.

**S8 Table. Internal validation of MERWACS across various subpopulations and scenarios (cont.)**

| **Subpopulations** | | | **Number** | **ROCAUC**  **X 100** | **PRAUC**  **X 100** | **PRAUC/Prevalence ratio** | **1-Brier Score**  **(%)** | **Sensitivity**  **(%)** | **Specificity**  **(%)** | **Balanced Accuracy**  **(%)** | **Accuracy**  **(%)** |
| --- | --- | --- | --- | --- | --- | --- | --- | --- | --- | --- | --- |
| **Age & Gender** | 50-54 | Female | 352 | 64.4  (57.9-70.6) | 43.7  (34.5-53.4) | 1.48  (1.22-1.77) | 80.2  (77.9-82.4) | 35.6  (26.4-44.5) | 84.6  (80.2-88.8) | 60.1  (54.9-65.2) | 70.1  (65.3-74.7) |
|  |  | Male | 318 | 70.6  (63.4-77.2) | 43.3  (32.6-54.0) | 1.94  (1.53-2.46) | 83.9  (82.0-85.8) | 42.1  (31.3-53.0) | 82.1  (77.3-86.5) | 62.1  (56.0-67.9) | 73.2  (68.5-78.0) |
|  | 55-59 | Female | 275 | 59.3  (51.3-67.1) | 33.5  (23.9-44.5) | 1.38  (1.06-1.76) | 81.5  (79.2-83.9) | 36.3  (25.4-47.5) | 77.0  (71.5-82.7) | 56.7  (50.2-63.0) | 67.1  (61.8-72.4) |
|  |  | Male | 289 | 59.7  (52.7-66.5) | 40.7  (30.7-51.1) | 1.44  (1.14-1.80) | 80.3  (78.1-82.7) | 39.0  (29.0-49.3) | 76.4  (70.3-82.1) | 57.7  (51.9-63.6) | 65.8  (60.2-71.6) |
|  | 60-64 | Female | 383 | 70.9  (65.2-76.3) | 52.3  (43.3-61.3) | 1.60  (1.38-1.85) | 80.4  (78.6-82.2) | 60.1  (51.3-69.1) | 72.6  (66.9-77.7) | 66.3  (60.9-71.6) | 68.5  (63.7-73.1) |
|  |  | Male | 358 | 67.2  (60.5-73.0) | 47.8  (38.8-58.2) | 1.62  (1.35-1.92) | 80.7  (78.9-82.5) | 58.6  (49.1-67.6) | 66.0  (60.5-71.3) | 62.3  (56.3-67.5) | 63.8  (58.7-68.4) |
|  | 65-69 | Female | 326 | 72.2  (66.1-77.8) | 56.6  (46.1-66.1) | 1.75  (1.47-2.05) | 81.0  (79.1-82.9) | 68.6  (60.4-77.6) | 66.4  (60.3-72.4) | 67.5  (62.3-73.0) | 67.1  (62.0-72.1) |
|  |  | Male | 311 | 68.5  (62.5-74.5) | 63.9  (54.7-71.5) | 1.54  (1.36-1.76) | 78.2  (76.1-80.0) | 69.0  (60.4-76.9) | 58.8  (51.6-66.1) | 63.9  (58.7-69.1) | 63.0  (57.9-68.2) |
|  | 70-74 | Female | 317 | 59.0  (52.0-65.7) | 47.1  (38.4-56.5) | 1.28  (1.08-1.50) | 77.1  (75.2-79.1) | 60.1  (51.2-69.1) | 50.0  (43.5-57.0) | 55.0  (49.4-61.1) | 53.7  (48.3-59.3) |
|  |  | Male | 287 | 71.9  (65.9-77.6) | 60.9  (51.3-70.2) | 1.60  (1.39-1.83) | 79.4  (77.7-81.0) | 76.4  (68.6-83.7) | 50.9  (43.4-58.2) | 63.7  (57.8-69.0) | 60.7  (54.7-66.2) |
|  | 75-79 | Female | 176 | 63.2  (54.7-71.6) | 57.9  (46.3-69.7) | 1.44  (1.19-1.74) | 77.3  (75.5-79.1) | 83.1  (74.3-91.2) | 25.0  (17.3-33.9) | 54.0  (48.1-60.5) | 48.5  (41.5-56.2) |
|  |  | Male | 174 | 62.2  (54.0-70.7) | 62.8  (52.1-73.4) | 1.19  (1.05-1.37) | 75.7  (73.7-77.6) | 81.5  (73.2-89.5) | 23.2  (14.6-32.9) | 52.3  (46.6-58.4) | 53.9  (46.6-60.9) |
|  | 80-84 | Female | 194 | 70.5  (62.8-77.7) | 73.3  (63.6-82.2) | 1.40  (1.22-1.59) | 77.8  (76.1-79.5) | 90.2  (84.5-95.7) | 29.6  (19.2-39.3) | 59.9  (54.3-65.5) | 61.5  (54.6-68.6) |
|  |  | Male | 220 | 64.0  (56.8-71.0) | 59.7  (49.6-69.2) | 1.25  (1.10-1.43) | 76.3  (75.0-77.6) | 95.3  (90.8-99.0) | 6.99  (2.68-12.2) | 51.1  (47.9-54.3) | 49.3  (42.7-55.9) |
|  | 85-89 | Female | 48 | 55.0  (37.6-72.0) | 56.7  (36.3-77.5) | 1.18  (0.876-1.61) | 74.8  (71.0-78.3) | 87.0  (70.8-100.) | 7.87  (0-20.7) | 47.4  (38.6-56.3) | 46.0  (33.3-60.4) |
|  |  | Male | 42 | 64.9  (47.2-81.9) | 72.7  (50.5-90.4) | 1.22  (0.964-1.57) | 76.6  (73.6-79.8) | 100.  (100.-100.) | 0  (0-0) | 50.0  (50.0-50.0) | 59.9  (42.9-76.2) |
|  | $\geq$90 | Female | 8 | 66.7  (14.3-100.) | 63.1  (7.14-100.) | 2.79  (0.571-8.00) | 83.6  (71.9-91.6) | 50.1  (0-100.) | 83.4  (50.0-100.) | 66.7  (30.8-100.) | 74.8  (50.0-100.) |
|  |  | Male | 7 | 65.5  (25.0-100.) | 93.5  (74.3-100.) | 1.11  (0.981-1.55) | 73.1  (64.6-80.5) | 83.2  (50.0-100.) | 0  (0-0) | 41.3  (20.0-50.0) | 71.3  (28.6-100.) |

Abbreviation: MERWACS, Machineborne Early Renal Warning And Control System; ROCAUC, area under the receiver operating characteristic curve; PRAUC, area under the precision-recall curve.

**S8 Table. Internal validation of MERWACS across various subpopulations and scenarios (cont.)**

| **Subpopulations** | | **Number** | **ROCAUC**  **X 100** | **PRAUC**  **X 100** | **PRAUC/Prevalence ratio** | **1-Brier Score**  **(%)** | **Sensitivity**  **(%)** | **Specificity**  **(%)** | **Balanced Accuracy**  **(%)** | **Accuracy**  **(%)** |
| --- | --- | --- | --- | --- | --- | --- | --- | --- | --- | --- |
| **Smoking**  **(cotinine in ng/mL)** | Lower than 14 | 3340 | 69.4  (67.5-71.3) | 56.7  (53.5-59.6) | 1.59  (1.52-1.67) | 79.6  (79.0-80.2) | 67.1  (64.4-69.9) | 60.3  (58.1-62.5) | 63.7  (62.0-65.4) | 62.7  (61.0-64.4) |
|  | Higher than or equal to 14 | 745 | 66.8  (62.7-70.8) | 47.8  (42.1-53.8) | 1.38  (1.24-1.53) | 78.9  (77.6-80.2) | 61.4  (55.2-67.2) | 64.9  (60.6-68.9) | 63.1  (59.5-66.7) | 63.7  (60.3-67.0) |
| **Ethnicity** | Mexican American | 587 | 67.9  (62.6-72.5) | 45.9  (37.6-54.0) | 1.76  (1.48-2.07) | 82.2  (80.9-83.6) | 52.9  (45.2-61.0) | 69.6  (65.3-74.0) | 61.3  (56.7-65.5) | 65.3  (61.5-69.0) |
|  | Other Hispanic | 162 | 69.7  (59.7-78.8) | 47.9  (32.9-62.2) | 1.91  (1.38-2.52) | 82.9  (79.8-85.6) | 44.0  (28.6-59.1) | 81.9  (75.0-88.6) | 63.0  (54.4-71.2) | 72.3  (65.4-79.0) |
|  | Non-Hispanic White | 2421 | 66.6  (64.3-68.7) | 51.8  (48.4-55.4) | 1.49  (1.41-1.59) | 79.2  (78.5-79.9) | 59.9  (56.7-63.2) | 64.7  (62.3-67.1) | 62.3  (60.3-64.3) | 63.0  (61.0-64.9) |
|  | Non-Hispanic Black | 722 | 66.9  (63.0-70.7) | 65.4  (60.2-70.6) | 1.33  (1.24-1.43) | 77.2  (76.3-78.0) | 92.1  (89.3-94.7) | 21.9  (18.1-26.2) | 57.0  (54.7-59.6) | 56.4  (52.9-60.1) |
|  | Other multirace | 193 | 68.1  (59.1-77.0) | 45.4  (32.8-60.5) | 1.62  (1.26-2.16) | 81.6  (78.5-84.4) | 43.9  (30.6-58.3) | 80.6  (73.8-87.0) | 62.3  (54.5-69.9) | 70.3  (63.7-76.7) |
| **BMI**  **(kg/m2)** | Lower than 30 | 2649 | 69.1  (66.9-71.3) | 53.7  (50.0-57.2) | 1.59  (1.50-1.70) | 80.0  (79.3-80.7) | 60.5  (57.1-63.8) | 66.3  (63.9-68.6) | 63.4  (61.3-65.4) | 64.3  (62.4-66.1) |
|  | Higher than or equal to 30 | 1436 | 67.9  (65.0-70.7) | 57.1  (52.8-61.3) | 1.49  (1.39-1.59) | 78.6  (77.7-79.4) | 75.0  (71.4-78.3) | 50.9  (47.8-54.2) | 62.9  (60.6-65.2) | 60.1  (57.8-62.5) |
| **Income**  **(poverty income ratio)** | Less than 1 | 585 | 66.4  (62.0-70.9) | 53.9  (47.2-61.4) | 1.43  (1.29-1.58) | 78.3  (76.9-79.5) | 72.0  (65.6-77.7) | 52.0  (46.8-57.3) | 62.0  (57.9-65.9) | 59.5  (55.6-63.8) |
|  | Between 1 and 2 | 1072 | 70.0  (67.0-73.1) | 60.1  (54.5-65.1) | 1.53  (1.42-1.65) | 78.9  (78.0-79.9) | 72.5  (68.1-76.7) | 52.7  (49.2-56.2) | 62.6  (59.9-65.4) | 60.5  (57.8-63.2) |
|  | Between 2 and 4 | 1181 | 69.4  (66.2-72.6) | 54.5  (49.2-59.5) | 1.58  (1.45-1.72) | 79.8  (78.8-80.8) | 69.7  (65.2-74.2) | 57.5  (54.0-60.9) | 63.6  (60.9-66.1) | 61.7  (59.0-64.3) |
|  | Greater than 4 | 1247 | 67.2  (63.7-70.6) | 50.8  (45.5-56.4) | 1.61  (1.46-1.77) | 80.3  (79.3-81.4) | 52.2  (47.1-57.3) | 74.9  (72.0-77.8) | 63.6  (60.5-66.5) | 67.7  (65.0-70.3) |
| **Education** | Up to high school graduation | 2295 | 70.5  (68.3-72.7) | 57.4  (53.7-60.9) | 1.56  (1.48-1.65) | 79.5  (78.9-80.2) | 71.6  (68.6-74.8) | 56.9  (54.2-59.3) | 64.2  (62.1-66.2) | 62.3  (60.2-64.3) |
|  | Higher education | 1790 | 66.2  (63.7-68.9) | 50.9  (46.9-55.3) | 1.52  (1.41-1.64) | 79.5  (78.7-80.3) | 58.3  (54.4-61.9) | 66.4  (63.9-68.9) | 62.3  (60.0-64.6) | 63.7  (61.5-65.8) |

Abbreviation: MERWACS, Machineborne Early Renal Warning And Control System; ROCAUC, area under the receiver operating characteristic curve; PRAUC, area under the precision-recall curve.
