# Supplementary figures and images for: MERWACS: Development and external validation of a non-invasive machine learning tool for identifying subjects to be screened for CKD
Source: PLOS Digit Health. 2026 Jul 9;5(7):e0001486. doi: 10.1371/journal.pdig.0001486 (PMC13349138; doi:10.1371/journal.pdig.0001486)

**S1 Fig.** Flowchart detailing the number of participants involved in the study


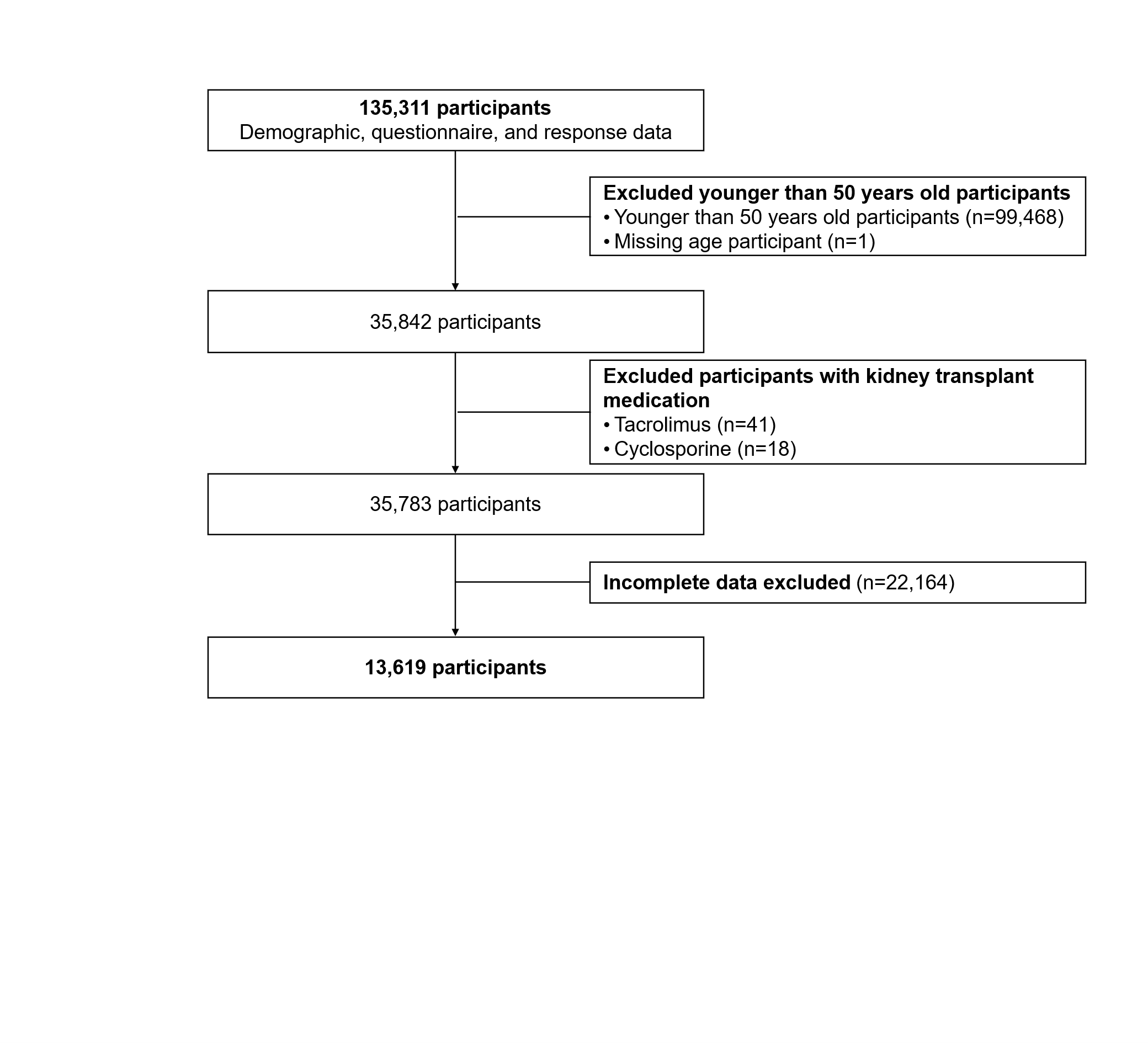

Supplement: S1 Fig — (DOCX) [file pdig.0001486.s010.docx]
