# Supplementary material for: MERWACS: Development and external validation of a non-invasive machine learning tool for identifying subjects to be screened for CKD
Source: PLOS Digit Health. 2026 Jul 9;5(7):e0001486. doi: 10.1371/journal.pdig.0001486 (PMC13349138; doi:10.1371/journal.pdig.0001486)
Supplement: S3 Fig — The figure compares the performance of the MERWACS model on the internal test and external validation datasets for the outcome defined using the CKD-EPI 2009 equation. (A) Discrimination performance across key metrics, calculated using an optimal probability cut-off of 0.284. (B) PRAUC-to-prevalence ratio. (C) Calibration plot. Abbreviations: MERWACS, Machineborne Early Renal Warning And Control System; CKD-EPI, Chronic Kidney Disease Epidemiology; ROCAUC, area under the receiver operating characteristic curve; PRAUC, area under the precision-recall curve; Sens., sensitivity; Spec., specificity; Bal. Acc., balanced accuracy; Acc., accuracy. (DOCX) [file pdig.0001486.s012.docx]

**S3 Fig.** Discrimination and calibration performances of MERWACS in predicting reduced kidney health status using CKD-EPI 2009 equation.

The figure compares the performance of the MERWACS model on the internal test and external validation datasets for the outcome defined using the CKD-EPI 2009 equation. **(A)** Discrimination performance across key metrics, calculated using an optimal probability cut-off of 0.284. **(B)** PRAUC-to-prevalence ratio. **(C)** Calibration plot.


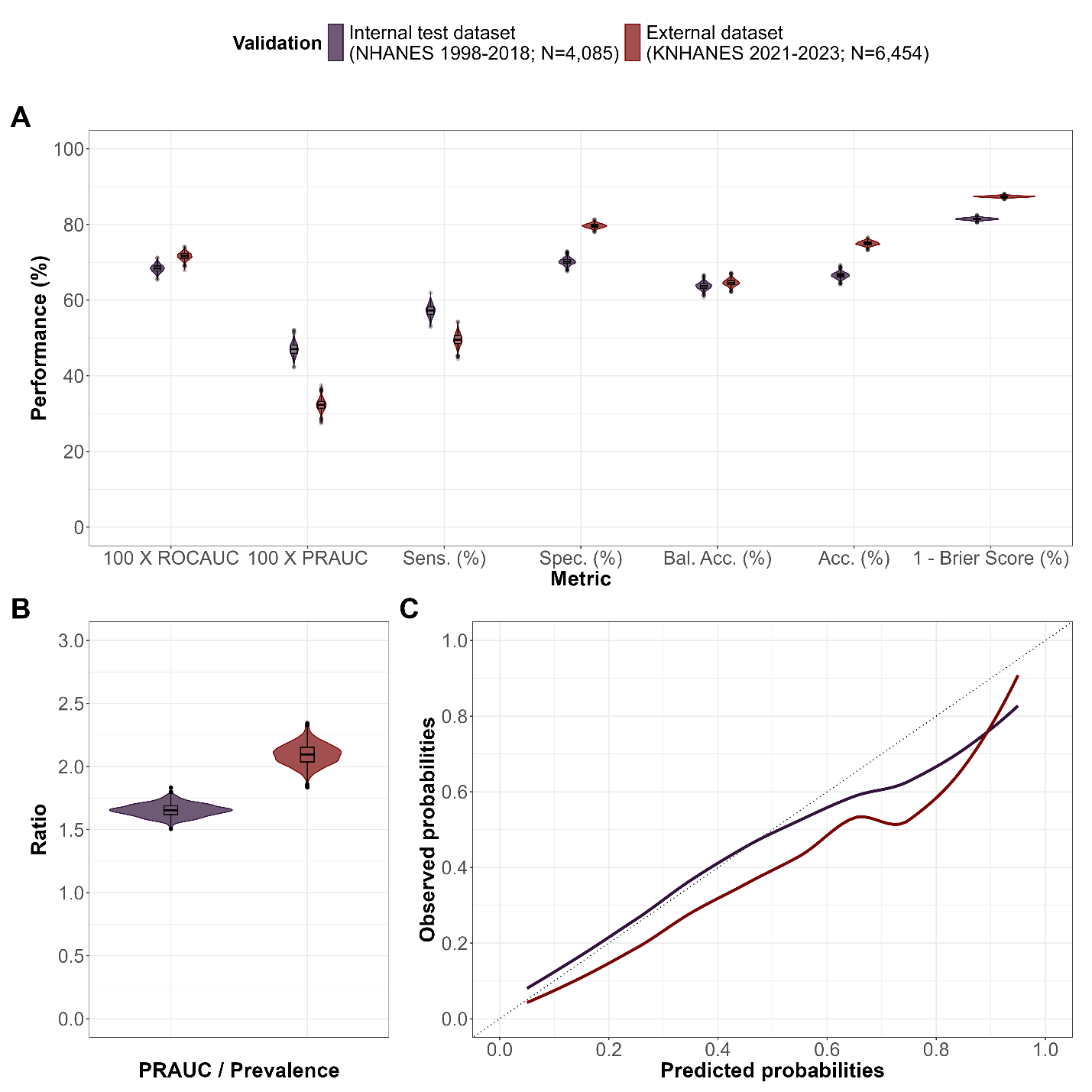


Abbreviations: MERWACS, Machineborne Early Renal Warning And Control System; CKD-EPI, Chronic Kidney Disease Epidemiology; ROCAUC, area under the receiver operating characteristic curve; PRAUC, area under the precision-recall curve; Sens., sensitivity; Spec., specificity; Bal. Acc., balanced accuracy; Acc., accuracy.
