# Supplementary material for: MERWACS: Development and external validation of a non-invasive machine learning tool for identifying subjects to be screened for CKD
Source: PLOS Digit Health. 2026 Jul 9;5(7):e0001486. doi: 10.1371/journal.pdig.0001486 (PMC13349138; doi:10.1371/journal.pdig.0001486)

**S4 Fig.** User Interface and functionality of the MERWACS online application.

The figure demonstrates the two primary functions of the open-access MERWACS web application, a tool designed to facilitate self-assessment and support clinical conversations about kidney health screening. (A) Prediction and ‘What-If’ Simulation Interface. This panel shows the main user interface. The specific example shown uses the European Kidney Function Consortium (EKFC)-based model to assess an 86-year-old individual with uncontrolled hypertension (systolic/diastolic blood pressure of 157/96 mmHg) and a weight of 80 kg, resulting in a “Current Predicted Probability” of 45.8%. The “Explore ‘What If’ Scenarios” feature then demonstrates that if this individual were to achieve a target weight of 54 kg and better blood pressure control (138/81 mmHg), the predicted probability drops substantially to 25.5%. The application provides general recommendations based on these probability tiers. (B) Case-Specific Prediction Interpretation (LIME). This panel displays the “How Parameters Impact Probability” tab, which provides model interpretability using a Local Interpretable Model-agnostic Explanations (LIME) plot. The plot visualizes the contribution of each input feature to the final predicted probability. Features with red bars are identified as increasing the probability, while features with blue bars are identified as decreasing it. The length of each bar is proportional to the magnitude of that feature’s influence, offering a transparent explanation of the “why” behind a user’s result. Abbreviation: MERWACS, Machineborne Early Renal Warning And Control System.


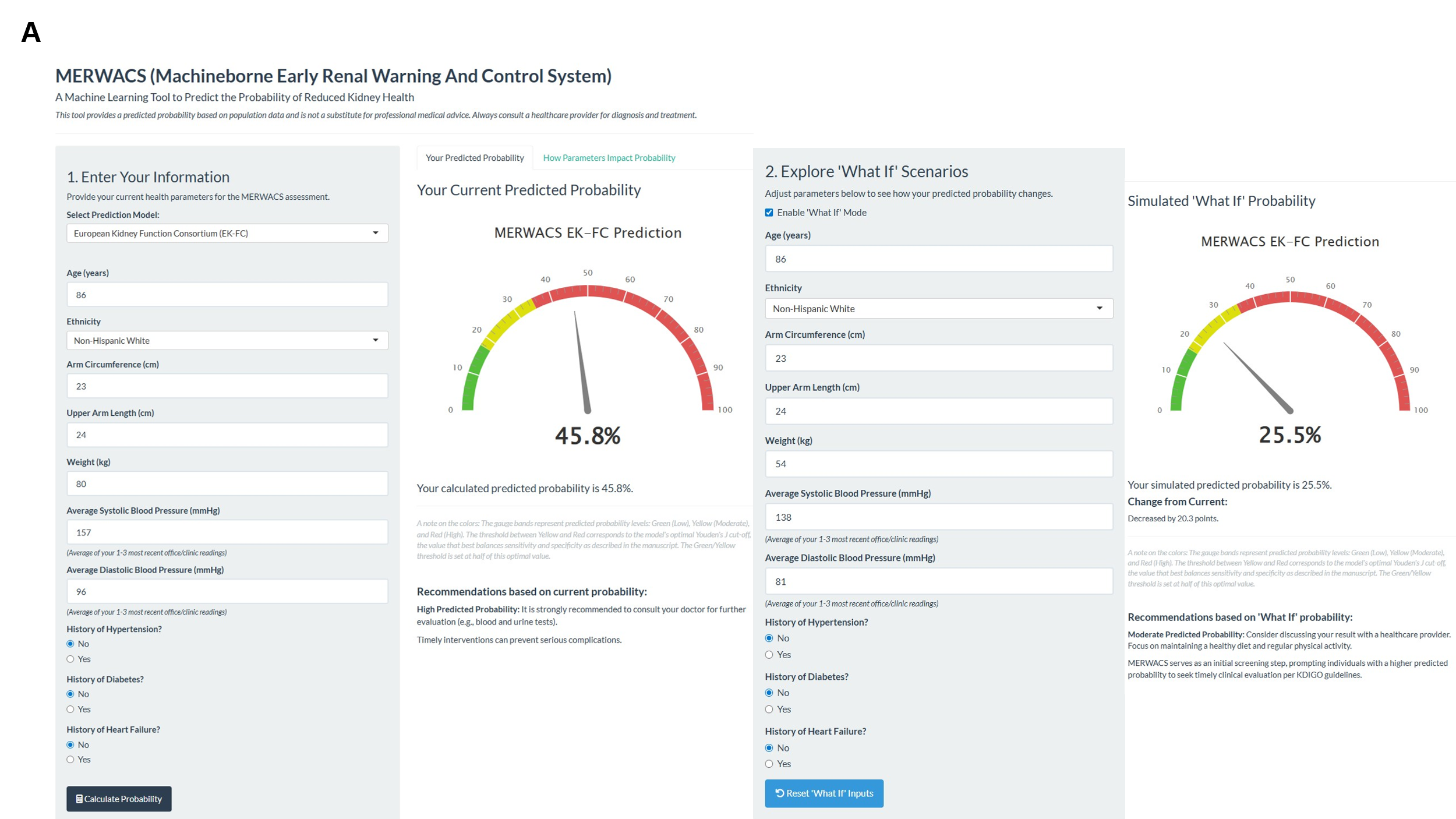

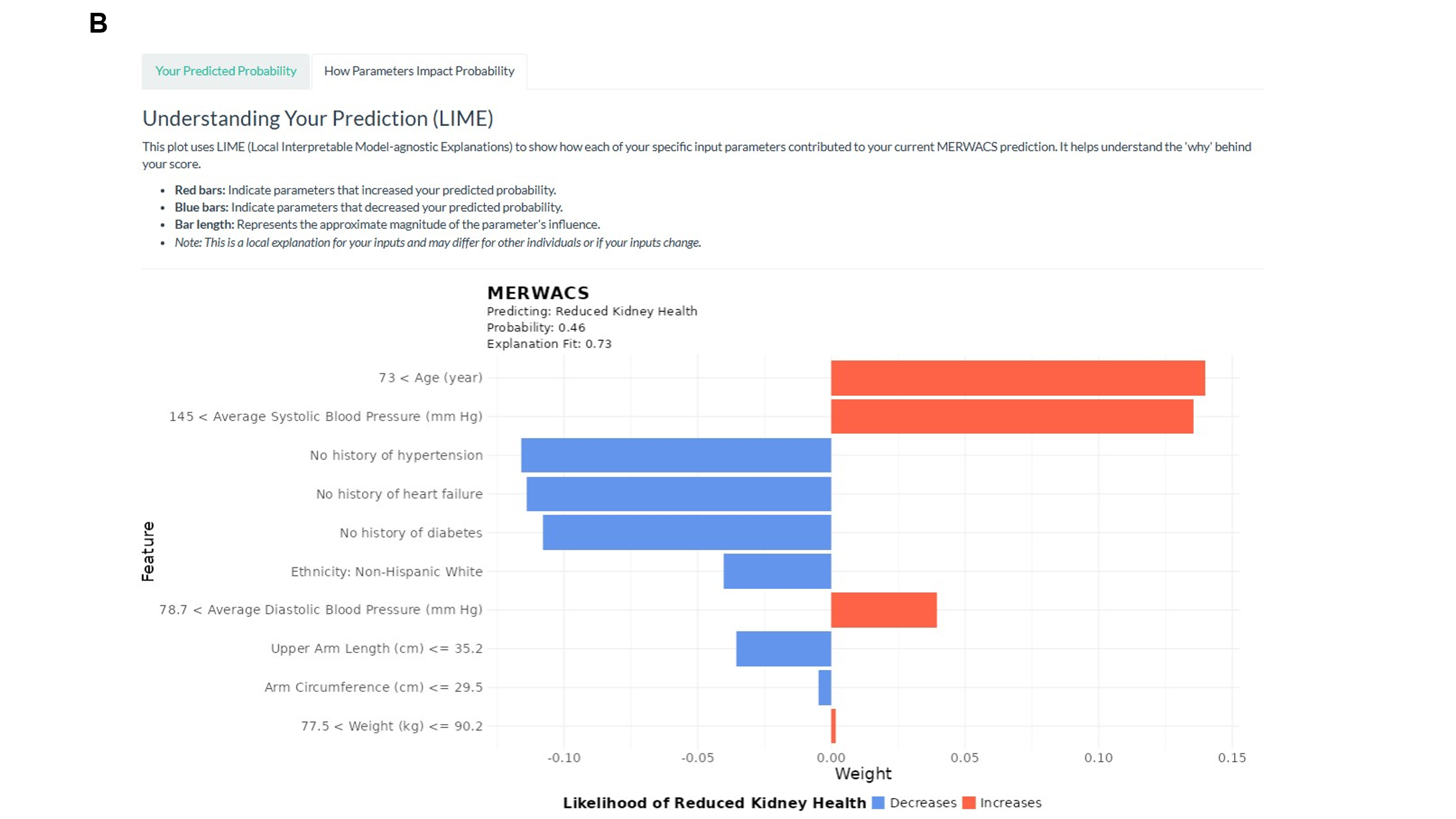

Supplement: S4 Fig — The figure demonstrates the two primary functions of the open-access MERWACS web application, a tool designed to facilitate self-assessment and support clinical conversations about kidney health screening. (A) Prediction and ‘What-If’ Simulation Interface. This panel shows the main user interface. The specific example shown uses the European Kidney Function Consortium (EKFC)-based model to assess an 86-year-old individual with uncontrolled hypertension (systolic/diastolic blood pressure of 157/96 mmHg) and a weight of 80 kg, resulting in a “Current Predicted Probability” of 45.8%. The “Explore ‘What If’ Scenarios” feature then demonstrates that if this individual were to achieve a target weight of 54 kg and better blood pressure control (138/81 mmHg), the predicted probability drops substantially to 25.5%. The application provides general recommendations based on these probability tiers. (B) Case-Specific Prediction Interpretation (LIME). This panel displays the “How Parameters Impact Probability” tab, which provides model interpretability using a Local Interpretable Model-agnostic Explanations (LIME) plot. The plot visualizes the contribution of each input feature to the final predicted probability. Features with red bars are identified as increasing the probability, while features with blue bars are identified as decreasing it. The length of each bar is proportional to the magnitude of that feature’s influence, offering a transparent explanation of the “why” behind a user’s result. Abbreviation: MERWACS, Machineborne Early Renal Warning And Control System. (DOCX) [file pdig.0001486.s013.docx]
