# Supplementary material for: MERWACS: Development and external validation of a non-invasive machine learning tool for identifying subjects to be screened for CKD
Source: PLOS Digit Health. 2026 Jul 9;5(7):e0001486. doi: 10.1371/journal.pdig.0001486 (PMC13349138; doi:10.1371/journal.pdig.0001486)
Supplement: Attachment — Submitted filename: reviewers_response_1st_revision.docx [file pdig.0001486.s015.docx]

**Authors’ responses to reviewers’ comments regarding the manuscript PDIG-D-25-00923 “MERWACS: Development and External Validation of a Non-Invasive Machine Learning Tool for Identifying Subject to be Screened for CKD”**

**Reviewer #1**

This manuscript presents the development and external validation of MERWACS, a non-invasive machine-learning–based screening tool for identifying adults who should undergo laboratory testing for chronic kidney disease (CKD). The study is carefully executed, methodologically rigorous, and transparently reported, with notable strengths including use of long-term NHANES data, external validation in a geographically distinct population (KNHANES), and strong emphasis on interpretability and usability via an open-access application. The work addresses an important public health gap: scalable, accessible pre-screening for CKD in the general adult population. However, several conceptual and methodological issues require clarification or strengthening to ensure appropriate interpretation, generalizability, and positioning of the tool within clinical and public health practice.

We sincerely thank the Reviewer for the thorough and constructive evaluation of our manuscript. The comments reflect a careful reading of the work and raise important points that we believe have substantially strengthened the manuscript. We have addressed each comment in detail below, and all changes are reflected in the revised manuscript with tracked changes.

**Major Issues**

**Comment 1:**

Conceptual framing of the outcome and its implications. The composite outcome, albuminuria (uACR ≥30 mg/g) or eGFR below the age- and sex-specific 2.5th percentile, is innovative and biologically motivated. However, this definition likely results in very high prevalence estimates (e.g., >30% using EKFC), which diverge substantially from conventional CKD prevalence estimates. While the authors acknowledge this, the implications are not fully explored. Specifically: how should users such as clinicians, patients, policymakers interpret a predicted probability of “reduced kidney health” when the outcome includes both pathological disease and early deviation from healthy reference norms?, does the tool risk over-identification and potential overuse of downstream testing, particularly in older adults? and, how does this definition align or intentionally not align with KDIGO staging and guideline based decision thresholds? A clearer articulation of how MERWACS complements (rather than conflicts with) existing CKD definitions and screening frameworks is needed.

We thank the reviewer for this insightful comment, which helps us clarify a key conceptual point. We agree that a more explicit discussion is warranted, and we have expanded the relevant sections accordingly.

We wish to first clarify the intended framing of “reduced kidney health.” This term was deliberately chosen to distinguish MERWACS’ outcome from a formal CKD diagnosis under KDIGO criteria, which requires persistence of markers over ≥3 months [KDIGO 2024 ref]. MERWACS identifies individuals who deviate from what would be expected for a healthy peer of the same age and sex, a signal that warrants further clinical evaluation, not a diagnosis in itself. The parallel with other screening tools is instructive: a positive mammography result does not constitute a cancer diagnosis, but it appropriately triggers definitive follow-up. MERWACS functions identically as a pre-screening triage tool.

On the risk of over-identification, particularly in older adults: we acknowledge this is a real trade-off inherent to the outcome definition. However, this is partly the point: the age- and sex-specific 2.5th percentile threshold was explicitly chosen to avoid the opposite error: mislabeling a healthy 80-year-old’s physiologically low eGFR as pathological under the conventional fixed <60 mL/min/1.73m^2^ cutoff. The consequence is indeed a higher screen-positive rate, which is appropriate at a population pre-screening stage (high sensitivity prioritized). The appropriate placement of MERWACS is upstream of, not instead of, clinical evaluation. To address this concern transparently, we have added the following passage to the Discussion:

*“It is important to contextualize what a screen-positive MERWACS result means. The tool is not designed to diagnose CKD under KDIGO criteria, which requires persistent markers of kidney damage for more than three months. Rather, a high predicted probability should be interpreted as a signal warranting clinical evaluation, including measurement of eGFR and uACR, which then feeds into formal KDIGO staging, positioning MERWACS explicitly upstream of existing clinical frameworks and complementary to them. Consistent with established screening principles, the tool prioritizes sensitivity over specificity to minimize missed cases at the population level [Wilson & Jungner, 1968], accepting a higher referral rate as a low-cost, low-risk trade-off relative to undiagnosed CKD.”*

**Comment 2:**

Clinical and public health utility relative to modest discrimination. The reported discrimination (ROCAUC ~0.68–0.73) is consistently moderate. While the authors correctly frame this as an intentional trade-off for accessibility, the manuscript would benefit from a more explicit discussion of decision utility. Authors should discuss on the use-case scenarios, how MERWACS would change practice beyond existing approaches.

We agree that moderate discrimination requires explicit justification through the intended use. A ROCAUC of ~0.70 would indeed be insufficient for a standalone diagnostic tool, but this is not MERWACS’ role. For population-level pre-screening, where the cost of a false negative (missed undiagnosed CKD) substantially outweighs the cost of a false positive (an unnecessary but inexpensive clinical conversation), moderate discrimination is operationally acceptable and consistent with other validated screening instruments in comparable settings [Bang et al., 2007; SCORED]. We have added the following to the Discussion to make the use-case context and decision logic explicit:

*“In practice, MERWACS adds value in three settings: (1) identifying individuals without a formal diagnosis of diabetes or hypertension but with a high cumulative burden of continuous risk factors; (2) supporting triage in non-clinical or low-resource environments where laboratory testing is unavailable; and (3) empowering individuals to self-assess and initiate informed clinical conversations. In each scenario, MERWACS prompts evaluation rather than replacing it. The observed discrimination (ROCAUC ~0.68–0.73) is operationally appropriate for this pre-screening role, consistent with other validated non-invasive CKD screening tools [Bang et al., 2007; SCORED], and reflects a deliberate trade-off that prioritizes sensitivity over specificity in line with established screening principles [Wilson & Jungner, 1968].”*

**Comment 3:**

Transportability and external validity beyond KNHANES. This is a major strength, however several limitations exist and poorly addressed. These are, 1) the outcome definition relies on age- and sex-specific eGFR percentiles derived from European reference cohorts, which may not be appropriate for Asian, African, or other populations; 2) Ethnicity is treated differently across datasets, raising concerns about stability of predictor effects.

We thank the reviewer for raising these important transportability concerns. Regarding the European-derived eGFR percentile thresholds, we agree this is a limitation and have strengthened the relevant passage in the limitations section.

*“The applicability of these specific percentile cutoffs to non-European ethnic populations is uncertain and represents a limitation, particularly for populations of African or South Asian origin where eGFR distributions and creatinine metabolism may differ systematically. Validating the outcome definition itself, not just the model, in these populations is an important future step.”*

Regarding ethnicity, we acknowledge that this predictor is not collected in KNHANES as it surveys an ethnically homogeneous Korean population [Kweon et al., 2019; KNHANES], and therefore required harmonization during external validation. More broadly, this means the stability of the ethnicity predictor across diverse populations cannot be fully assessed from the current data, and we have added explicit acknowledgment of this to the limitations.

*“Additionally, ethnicity (one of the predictors in MERWACS) is a variable that KNHANES does not collect, as it surveys an ethnically homogeneous Korean population [Kweon et al., 2019; KNHANES]. This structural difference limits our ability to assess predictor stability across ethnic groups, and future validation in ethnically diverse non-U.S. populations would be needed to fully characterize this source of variability.”*

**Comment 4:**

Cross-sectional design and inability to predict incident disease. I think MERWACS predicts prevalent reduced kidney function, not incident CKD. This must be fixed in entire manuscript.

We agree that the prevalent nature of the prediction must be unambiguous throughout. We note that the limitations section already explicitly states that “MERWACS predicts the probability of prevalent (existing) reduced kidney function, not incident (future) disease”. However, we agree this distinction should be visible earlier in the manuscript. We have added the word “prevalent” explicitly to the abstract and ensured the introduction does not overstate the tool’s scope regarding incident disease prediction.

**Manuscript changes:**

1. Abstract

Current: “The model predicts a composite outcome of reduced kidney function…”

Revised: *“The model predicts a composite outcome of prevalent reduced kidney function…”*

2. Abstract conclusion

Current: “MERWACS is a robust, externally validated, non-invasive tool that helps in identifying which adult and elderly individuals should be screened for CKD.”

Revised: *“MERWACS is a robust, externally validated, non-invasive tool that identifies adults and elderly individuals with a high probability of currently having reduced kidney health, helping prioritize them for definitive CKD screening.”*

3. Introduction

Current: “With the recent advent of highly effective therapies that can slow CKD progression … the imperative for early detection has never been greater.”

Revision*: “With the recent advent of highly effective therapies that can slow CKD progression … the imperative for identifying undiagnosed prevalent CKD in the general population has never been greater.”*

**Comment 5:**

Discussion would benefit from a more explicit treatment of implementation science considerations like safeguards, communication strategies, and integration with healthcare systems.

We agree that implementation science considerations are important for a tool intended for real-world deployment and have added a dedicated paragraph to the Discussion addressing safeguards, communication strategies, and integration with healthcare pathways.

*“Translating MERWACS into practice raises important implementation considerations. First, regarding safeguards, the online application is explicitly framed as a pre-screening aid rather than a diagnostic tool, and results are accompanied by a recommendation to seek clinical evaluation for individuals with elevated predicted probabilities. To minimize misinterpretation, probability outputs are presented in tiered categories with plain-language guidance rather than as raw numerical values alone. Second, regarding communication, the tool is designed for dual use: individuals can self-assess and arrive at clinical encounters with greater awareness of their kidney health status, while clinicians can use the predicted probability to support and document screening decisions. Third, regarding integration with healthcare systems, MERWACS is most naturally positioned at points of care with broad population reach but limited laboratory access, such as community pharmacies, telemedicine platforms, or public health screening programs. Future implementation should involve prospective evaluation of whether MERWACS-guided triage increases the rate of appropriate laboratory testing and early CKD detection in these settings.”*

**Minor issues**

**Comment 1:**

Model comparison interpretation. Differences between XGBoost and logistic regression are modest. The rationale for preferring ML over simpler models should be more carefully framed around interpretability and flexibility rather than performance alone.

We thank the reviewer for this observation. We agree that the primary justification for XGBoost over logistic regression should rest on interpretability and flexibility rather than marginal performance gains. We have revised the relevant passage in the Discussion accordingly.

*“Furthermore, the primary rationale for adopting a non-linear XGBoost model over logistic regression lies in its flexibility to capture complex, non-linear predictor interactions and its compatibility with SHAP and LIME explainability frameworks, which are essential for clinical interpretability and user trust. The modest but consistent discrimination advantage over logistic regression across both internal and external validation datasets provides additional, though secondary, support for this choice.”*

**Comment 2:**

Exclusion of dietary variables is reasonable, but a brief justification of why other non-invasive variables (e.g., physical activity) were not retained would improve transparency.

Physical activity is indeed an example of a non-invasive predictor that aligns with the accessibility criteria of MERWACS, but it was excluded due to data limitations inherent to the 30-year NHANES dataset. Questions regarding physical activity changed substantially across survey cycles, resulting in high rates of missing data for harmonized variables in our final dataset, for example, 40.0% missing for the muscle strengthening question (PAD440) and 65.2% missing for moderate household tasks (PAQ100) among the 13,619 study participants. As our candidate predictor filtering applied a 20% missingness threshold to ensure data continuity and statistical robustness, these variables were excluded at that stage. We have added a brief clarification to the Methods to make this reasoning transparent.

*“For example, physical activity variables, though conceptually aligned with our accessibility criteria, were excluded at this stage due to substantial missingness arising from changes in survey questionnaires across NHANES cycles (e.g., 40.0% missing for PAD440 and 65.2% for PAQ100).”*

**Comment 3:**

Calibration plots are informative, but numerical calibration metrics stratified by age group would further strengthen confidence, given the age-centric outcome definition.

We now have computed calibration-in-the-large metrics, specifically intercept and slope with 95% confidence intervals, stratified by 5-year age group for both the internal and external validation datasets, across all three eGFR equation-based models. The methodology has been added to the Methods section (Model Performance Evaluation), with the following text inserted after the bootstrap CI sentence:

*“In addition, we quantified calibration numerically using two summary metrics: the calibration-in-the-large intercept, which captures systematic bias in predicted probabilities overall (perfect value = 0; negative values indicate overprediction), and the calibration slope, which captures whether predicted probabilities are appropriately spread (perfect value = 1; values below 1 indicate overconfidence). Both metrics were estimated via logistic regression of the binary outcome on the log-odds of the calibrated predicted probabilities [Van Calister et al., 2016].”*

For the primary EKFC-based model, the overall internal intercept was -0.010 (95% CI -0.080 to 0.059) and slope was 0.754 (95% CI 0.673 to 0.838), indicating near-zero systematic bias but modest overconfidence in predicted probabilities. The following sentence has been added to the “MERWACS Performance on Internal Validation” section:

*“Calibration-in-the-large metrics for the primary EKFC-based model confirmed good overall calibration on the internal test set, with an intercept of -0.010 (95% CI -0.080 to 0.059) and a slope of 0.754 (95% CI 0.673 to 0.838), indicating near-zero systematic bias but modest overconfidence in predicted probabilities. Age-stratified calibration metrics are provided in* ***Supplementary Table S7****.”*

Externally, the overall intercept was -0.441 (95% CI -0.509 to -0.374), reflecting a systematic tendency toward overprediction in the KNHANES population, while the slope of 0.957 (95% CI 0.865 to 1.05) remained close to 1. The following sentence has been added to the “External Validation of MERWACS” section:

*“Calibration-in-the-large metrics indicated a systematic tendency toward overprediction in the KNHANES population, with an overall intercept of -0.441 (95% CI -0.509 to -0.374), consistent across all age groups. The calibration slope of 0.957 (95% CI 0.865 to 1.05) was close to 1, suggesting the spread of predicted probabilities was appropriate. Full age-stratified calibration metrics are provided in* ***Supplementary Table S7****.”*

Full age-stratified results across all three equations are provided in the new **Supplementary Table S7**, reproduced below for convenience:

**Supplementary Table S7. Calibration-in-the-large metrics for MERWACS across three eGFR equations, overall and stratified by age group, for internal and external validation datasets**

Calibration intercept and slope for the MERWACS model computed for the internal test set (NHANES 1988–2018; n=4,085) and external validation dataset (KNHANES 2021–2023; n=6,454), overall and within each 5-year age stratum, for all three eGFR equation-based models. Values are presented as estimate (95% confidence interval).

| **Dataset** | **Age**  **group** | **EKFC** | | **CKD-EPI 2021** | | **CKD-EPI 2009** | |
| --- | --- | --- | --- | --- | --- | --- | --- |
|  |  | **Intercept** | **Slope** | **Intercept** | **Slope** | **Intercept** | **Slope** |
| **Internal** | 50-54 | 0.0484 (-0.135;0.227) | 0.789 (0.535;1.05) | 0.177 (-0.0302;0.377) | 0.796 (0.561;1.04) | 0.0872 (-0.108;0.276) | 0.849 (0.551;1.16) |
|  | 55-59 | -0.0611 (-0.262;0.135) | 0.491 (0.248;0.742) | -0.149 (-0.387;0.0787) | 0.195 (0.0320;0.397) | -0.0810 (-0.300;0.130) | 0.551 (0.239;0.867) |
|  | 60-64 | -0.0718 (-0.241;0.0939) | 0.750 (0.553;0.957) | -0.0374 (-0.223;0.144) | 0.745 (0.558;0.939) | 0.00209 (-0.175;0.175) | 1.01 (0.768;1.26) |
|  | 65-69 | 0.0752 (-0.100;0.248) | 0.882 (0.665;1.11) | 0.00830 (-0.188;0.200) | 0.708 (0.508;0.916) | 0.124 (-0.0584;0.303) | 0.928 (0.688;1.18) |
|  | 70-74 | -0.00720 (-0.184;0.167) | 0.673 (0.448;0.908) | 0.0908 (-0.100;0.277) | 0.547 (0.339;0.761) | 0.0131 (-0.172;0.194) | 0.703 (0.459;0.954) |
|  | 75-79 | -0.0539 (-0.278;0.169) | 0.520 (0.254;0.812) | -0.0928 (-0.335;0.143) | 0.573 (0.287;0.870) | -0.0607 (-0.294;0.168) | 0.719 (0.409;1.04) |
|  | 80-84 | -0.0582 (-0.262;0.145) | 0.783 (0.488;1.11) | -0.0715 (-0.288;0.141) | 0.646 (0.377;0.927) | -0.0325 (-0.243;0.175) | 0.687 (0.413;0.975) |
|  | 85-89 | 0.0707 (-0.363;0.507) | 0.339 (-0.197;0.980) | -0.0688 (-0.530;0.377) | 0.0744 (-0.124;0.322) | 0.131 (-0.310;0.565) | -0.0374 (-0.422;0.245) |
|  | 90+ | 0.625 (-0.449;1.72) | 2.52 (0.438;5.59) | 0.542 (-0.581;1.60) | 7.04 (2.07;17.7) | 0.371 (-0.752;1.43) | 13.1 (3.55; NA) |
|  | **Overall** | -0.00990 (-0.0795;0.0593) | 0.754 (0.673;0.838) | -0.00162 (-0.0783;0.0743) | 0.621 (0.543;0.700) | 0.0201 (-0.0528;0.0925) | 0.800 (0.709;0.892) |
| **External** | 50-54 | -0.560 (-0.772;-0.358) | 0.720 (0.410;1.04) | -0.185 (-0.414;0.0317) | 0.540 (0.247;0.872) | -0.533 (-0.749;-0.328) | 1.23 (0.891;1.59) |
|  | 55-59 | -0.288 (-0.477;-0.106) | 0.891 (0.600;1.20) | -0.102 (-0.309;0.0953) | 0.910 (0.606;1.22) | -0.338 (-0.535;-0.149) | 1.08 (0.774;1.40) |
|  | 60-64 | -0.445 (-0.621;-0.275) | 0.978 (0.705;1.26) | -0.353 (-0.550;-0.165) | 1.14 (0.859;1.42) | -0.491 (-0.677;-0.312) | 0.968 (0.702;1.24) |
|  | 65-69 | -0.367 (-0.530;-0.209) | 1.00 (0.738;1.28) | -0.140 (-0.314;0.0273) | 1.18 (0.916;1.45) | -0.329 (-0.497;-0.167) | 1.12 (0.847;1.40) |
|  | 70-74 | -0.380 (-0.554;-0.211) | 1.22 (0.908;1.54) | -0.208 (-0.395;-0.0279) | 1.10 (0.815;1.39) | -0.384 (-0.567;-0.207) | 1.13 (0.823;1.45) |
|  | 75-79 | -0.564 (-0.741;-0.391) | 1.17 (0.836;1.52) | -0.429 (-0.623;-0.242) | 1.21 (0.886;1.56) | -0.515 (-0.702;-0.334) | 1.15 (0.839;1.49) |
|  | 80-84 | -0.506 (-0.695;-0.322) | 0.950 (0.594;1.33) | -0.455 (-0.660;-0.257) | 0.864 (0.514;1.23) | -0.482 (-0.682;-0.288) | 0.922 (0.583;1.27) |
|  | **Overall** | -0.441 (-0.509;-0.374) | 0.957 (0.865;1.05) | -0.270 (-0.344;-0.198) | 0.940 (0.847;1.03) | -0.434 (-0.505;-0.364) | 1.04 (0.946;1.15) |

**Comment 4:**

Editorial issues (e.g., “Subject” vs. “Subjects” in the title) should be corrected.

The title has been corrected from “Subject” to “Subjects.”

**Comment 5:**

Some figures are dense; improving labeling

We have reviewed the figures and improved label clarity in Figure 5, where the six-panel LIME layout contained the densest labeling. In addition, all figures have been reformatted to comply with PLOS Digital Health figure requirements, including updated dimensions, resolution, and font sizes. Updated figure files are included with the revised submission, and the revised Figure 5 is shown below for convenience.

**Figure 5. Case-specific interpretations of MERWACS predictions using LIME**

**Reviewer #2:**

**Comment 1:**

The use of European-derived age- and sex-specific eGFR percentiles as the outcome reference for U.S. and Korean populations may introduce systematic misclassification. I suggest justifying the transferability of European eGFR percentiles, because applying these norms to U.S. and Korean populations may bias outcome classification across ethnic groups.

We thank the reviewer for raising this concern, which was also noted by Reviewer #1. We fully acknowledge that applying European-derived eGFR percentile thresholds to U.S. and Korean populations may introduce systematic misclassification, particularly across ethnic groups where creatinine metabolism and eGFR distributions may differ. We have strengthened the limitations section to reflect this explicitly, with the following addition:

*“The applicability of these specific percentile cutoffs to non-European ethnic populations is uncertain and represents a limitation, particularly for populations of African or South Asian origin where eGFR distributions and creatinine metabolism may differ systematically. Validating the outcome definition itself, not just the model, in these populations is an important future step.”*

As an additional mitigating argument, we note that our three-equation robustness framework, which includes the race-free CKD-EPI 2021 equation, was specifically designed to reduce dependence on any single set of population-derived norms. The consistent performance across all three equations increases our confidence that the findings are not driven by the specific characteristics of the European reference cohort. Nevertheless, we agree that validating the outcome definition itself in non-European populations remains an important future step, and this is now stated explicitly in the revised limitations.

**Comment 2:**

The very high prevalence of the composite outcome (up to ~35%) risks blurring the distinction between “screen-positive” and “clinically meaningful disease.”

I recommend quantifying potential misclassification, since the 2.5th percentile threshold leads to unusually high prevalence and may label physiologic aging as “reduced kidney function.”

We thank the reviewer for this important point. We agree that transparently quantifying the contribution of each outcome component to the overall composite prevalence is valuable, and we have added a new **Supplementary Table S2** to address this directly.

As shown in **Supplementary Table S2**, for the primary EKFC-based model in NHANES, the composite prevalence of 35.3% comprises three mutually exclusive groups: participants classified by low eGFR alone (19.0%), by elevated uACR alone (10.4%), and by both criteria simultaneously (6.0%). Two points are important in interpreting these numbers. First, the relatively large eGFR-only contribution under the EKFC equation reflects the sensitivity of this equation to age-related eGFR decline, which is by design — the 2.5th percentile threshold was explicitly chosen to be more conservative than the fixed eGFR < 60 cutoff for older adults, as demonstrated by Eriksen et al. (2020), where the 2.5th percentile for many elderly strata falls below 60 mL/min/1.73m^2^. Second, for the CKD-EPI 2021 and CKD-EPI 2009 equations, the eGFR-only contribution drops substantially to 9.7% and 11.7% respectively, converging with the uACR-only contribution, suggesting the high EKFC prevalence is largely equation-specific rather than reflecting systematic mislabeling of physiologic aging. In KNHANES, the uACR component dominates (9.4–10.3%) over the eGFR component (1.9–4.9%). These findings support the validity of the composite outcome design and are now acknowledged in the manuscript.

**Prevalence of Reduced Kidney Function**

*“To further characterize the composite outcome, we decomposed the prevalence into mutually exclusive contributions from each criterion. For the primary EKFC-based model in NHANES, 19.0% of participants were classified by low eGFR alone, 10.4% by elevated uACR alone, and 6.0% by both criteria simultaneously. The eGFR-only contribution was lower for the CKD-EPI 2021 (9.7%) and CKD-EPI 2009 (11.7%) equations, reflecting differences in equation sensitivity to age-related eGFR decline. Full decomposition results for both datasets are provided in* ***Supplementary Table S2****.”*

**External Validation of MERWACS**

*“Decomposition of the composite outcome in KNHANES revealed that the uACR component was the predominant driver of classification, with 9.4% of participants classified by elevated uACR alone compared to 4.9% by low eGFR alone (EKFC-based model). Full decomposition results are provided in* ***Supplementary Table S2****.”*

**Supplementary Table S2. Decomposition of the composite reduced kidney function outcome by individual criterion, for NHANES and KNHANES datasets across three eGFR equations**

The table presents the number and percentage of participants classified into four mutually exclusive groups based on the two components of the composite outcome: low eGFR (below the age- and sex-specific 2.5^th^ percentile from Eriksen et al., 2020) and elevated uACR (urine albumin-to-creatinine ratio ≥ 30 mg/g). The composite row corresponds to the primary outcome used in the manuscript (one or both criteria met) and serves as an internal consistency check against the prevalence figures reported in Table 1.

| **Outcome component** | **NHANES (n=13,619)** | | | **KNHANES (n=6,454)** | | |
| --- | --- | --- | --- | --- | --- | --- |
|  | **EKFC** | **CKD-EPI 2021** | **CKD-EPI 2009** | **EKFC** | **CKD-EPI 2021** | **CKD-EPI 2009** |
| Neither | 8805 (64.7%) | 10069 (73.9%) | 9798 (71.9%) | 5345 (82.8%) | 5538 (85.8%) | 5459 (84.6%) |
| Low eGFR only | 2585 (19%) | 1321 (9.7%) | 1592 (11.7%) | 318 (4.9%) | 125 (1.9%) | 204 (3.2%) |
| High uACR only | 1417 (10.4%) | 1665 (12.2%) | 1611 (11.8%) | 605 (9.4%) | 664 (10.3%) | 636 (9.9%) |
| Both | 812 (6%) | 564 (4.1%) | 618 (4.5%) | 186 (2.9%) | 127 (2%) | 155 (2.4%) |
| **Composite (any criterion)** | 4814 (35.3%) | 3550 (26.1%) | 3821 (28.1%) | 1109 (17.2%) | 916 (14.2%) | 995 (15.4%) |

Abbreviations: CKD-EPI, Chronic Kidney Disease Epidemiology Collaboration; EKFC, European Kidney Function Consortium; KNHANES, Korea National Health and Nutrition Examination Survey; NHANES, National Health and Nutrition Examination Survey; uACR, urine albumin-to-creatinine ratio.

**Comment 3:**

The external validation relies partly on imputed predictors, which weakens claims of real-world deployability. Suggest expanding discussion on external imputation, as imputing unavailable KNHANES predictors could inflate external validation performance.

We agree that imputing unavailable predictors during external validation could in principle inflate performance estimates, and we want to highlight that we specifically anticipated and addressed this concern through a pre-specified sensitivity analysis. In this analysis, we evaluated a model version that entirely excluded the two predictors unavailable in KNHANES, a history of congestive heart failure and arm length, rather than imputing them. This reduced model achieved a ROCAUC of 0.711 (95% CI 0.694–0.727) on the external dataset, compared to 0.712 for the full model, confirming that the imputation had a negligible impact on external validation performance. We have expanded the limitations section to make this reasoning more explicit, with the following addition to the existing imputation limitation paragraph.

*“Importantly, our pre-specified sensitivity analysis demonstrated that excluding these imputed predictors entirely yielded virtually identical external validation performance (ROCAUC 0.711 vs. 0.712), providing direct empirical evidence that the imputation did not meaningfully inflate the reported external validation results”*

**Comment 4:**

The incremental value over simpler logistic regression is statistically modest. please clarify the clinical meaning of a positive MERWACS result, because “screen-positive” does not clearly map to guideline-based CKD diagnosis.

We thank the reviewer for these important questions. As we also noted in our response to Reviewer #1 regarding the incremental value over logistic regression, we agree the performance difference is modest. Accordingly, we have revised the Discussion to clarify that the primary justification for XGBoost lies in its flexibility to capture non-linear predictor interactions and its compatibility with SHAP and LIME explainability frameworks, rather than discrimination gain alone (Discussion).

*“Furthermore, the primary rationale for adopting a non-linear XGBoost model over logistic regression lies in its flexibility to capture complex, non-linear predictor interactions and its compatibility with SHAP and LIME explainability frameworks, which are essential for clinical interpretability and user trust. The modest but consistent discrimination advantage over logistic regression across both internal and external validation datasets provides additional, though secondary, support for this choice.”*

Regarding the clinical meaning of a screen-positive result, we fully agree this needed clarification and have added a dedicated paragraph to the Discussion addressing this explicitly. A high predicted probability from MERWACS should not be interpreted as a CKD diagnosis under KDIGO criteria, which requires persistent markers of kidney damage over more than three months. Rather, it identifies individuals who deviate sufficiently from their healthy age- and sex-matched peers to warrant clinical evaluation, including measurement of eGFR and uACR, which then feeds into formal KDIGO staging. The following text has been added to the Discussion:

*“It is important to contextualize what a screen-positive MERWACS result means. The tool is not designed to diagnose CKD under KDIGO criteria, which requires persistent markers of kidney damage for more than three months. Rather, a high predicted probability should be interpreted as a signal warranting clinical evaluation, including measurement of eGFR and uACR, which then feeds into formal KDIGO staging, positioning MERWACS explicitly upstream of existing clinical frameworks and complementary to them. Consistent with established screening principles, the tool prioritizes sensitivity over specificity to minimize missed cases at the population level [Wilson and Jungner, 1968], accepting a higher referral rate as a low-cost, low-risk trade-off relative to undiagnosed CKD.”*

**Comment 5:**

need to report decision-curve analysis

We now have conducted a decision curve analysis (DCA) on the internal test set (NHANES; n=4,085) using the primary EKFC-based outcome, comparing MERWACS against five reference strategies: Screen All, Screen None, age-based screening (≥55 years ; De Nicola et al., 2025), hypertension-based screening, and a simplified KDIGO-based strategy (known diabetes or hypertension; KDIGO 2024). The following text has been added as a new subsection in the Methods:

**Decision Curve Analysis**

*“To evaluate the clinical utility of MERWACS, we conducted a decision curve analysis (DCA) on the internal test set using the primary EKFC-based outcome [Vickers et al., 2016; DCA]. DCA quantifies the net benefit of a prediction model across a range of threshold probabilities, accounting for both the benefit of true positives and the harm of false positives weighted by the chosen threshold. We compared MERWACS against five strategies: (1) Screen All, (2) Screen None, (3) age-based screening, defined as all individuals aged 55 years or older, consistent with cost-effectiveness estimates for CKD screening in the general population, (4) hypertension-based screening, defined as a self-reported diagnosis of hypertension, and (5) a simplified KDIGO-based strategy, defined as a self-reported diagnosis of diabetes or hypertension, consistent with KDIGO 2024 screening recommendations.”*

The following text has been added as a new subsection in the Results:

**Decision Curve Analysis**

*“To evaluate the clinical utility of MERWACS, we conducted a decision curve analysis comparing MERWACS against five reference strategies: Screen All, Screen None, age-based screening (≥55 years), hypertension-based screening, and a simplified KDIGO-based strategy (known diabetes or hypertension). As shown in Figure 6, MERWACS provided consistently higher net benefit than all three active comparator strategies across the full range of threshold probabilities. Age-based and Screen All strategies declined rapidly, reaching negative net benefit around 35%. The KDIGO-simplified and hypertension-based strategies went negative around 50%, while MERWACS maintained positive net benefit up to approximately 75%.”*

The DCA figure is included as the new Figure 6 in the revised manuscript.

**Figure 6. Decision curve analysis of MERWACS for the primary EKFC-based outcome**

We performed a decision curve analysis (DCA) to assess the clinical utility of MERWACS in identifying individuals with reduced kidney health. DCA compared six strategies: (1) Screen All: screening all individuals, (2) Screen None: screening no individuals, (3) Age-based: screening all individuals aged ≥55 years, consistent with cost-effectiveness estimates for CKD screening (De Nicola et al., 2025), (4) Hypertension-based: screening individuals with a self-reported diagnosis of hypertension, (5) KDIGO-simplified: screening individuals with a self-reported diagnosis of diabetes or hypertension, consistent with KDIGO 2024 recommendations, and (6) MERWACS: selecting individuals for screening based on the calibrated predicted probability. The analysis accounted for false positive and false negative rates to determine the net benefit of each strategy. Abbreviations: DM, diabetes mellitus; EKFC, European Kidney Function Consortium; HTN, hypertension; KDIGO, Kidney Disease Improving Global Outcomes; MERWACS, Machineborne Early Renal Warning And Control System.

**Comment 6.**

comparing performance to age-only or hypertension-only models

We incorporated these directly as comparator strategies within the decision curve analysis (**Figure 6**), which provides a clinically informative and integrated comparison. In addition, we included a simplified KDIGO-based strategy (known diabetes or hypertension; KDIGO 2024) as a further comparator, representing current guideline-based screening practice. The DCA demonstrates that age-based screening (≥55 years; De Nicola et al., 2025), hypertension-based screening, and the KDIGO-simplified strategy are all outperformed by MERWACS across the full range of threshold probabilities, with all three strategies going negative well before MERWACS, which maintains positive net benefit up to approximately 75%.

**Comment 7.**

propose adding calibration-in-the-large metrics

We now have added calibration-in-the-large metrics, specifically intercept and slope with 95% confidence intervals, for both the internal and external validation datasets, overall and stratified by 5-year age group, across all three eGFR equation-based models. The methodology has been added to the Methods section (Model Performance Evaluation), with the following text:

*“In addition, we quantified calibration numerically using two summary metrics: the calibration-in-the-large intercept, which captures systematic bias in predicted probabilities overall (perfect value = 0; negative values indicate overprediction), and the calibration slope, which captures whether predicted probabilities are appropriately spread (perfect value = 1; values below 1 indicate overconfidence). Both metrics were estimated via logistic regression of the binary outcome on the log-odds of the calibrated predicted probabilities [Van Calister et al., 2016].”*

Key overall values have been added to the Results section. For the primary EKFC-based model, the internal overall intercept was -0.010 (95% CI -0.080 to 0.059) and slope was 0.754 (95% CI 0.673 to 0.838). Externally, the intercept of -0.441 (95% CI -0.509 to -0.374) indicated systematic overprediction in the KNHANES population, likely reflecting the lower outcome prevalence in that dataset (17.2% vs. 35.3% in NHANES), while the slope of 0.957 (95% CI 0.865 to 1.05) was close to 1. Full age-stratified results are provided in the new **Supplementary Table S7**, reproduced below for convenience:

**Supplementary Table S7. Calibration-in-the-large metrics for MERWACS across three eGFR equations, overall and stratified by age group, for internal and external validation datasets**

Calibration intercept and slope for the MERWACS model computed for the internal test set (NHANES 1988–2018; n=4,085) and external validation dataset (KNHANES 2021–2023; n=6,454), overall and within each 5-year age stratum, for all three eGFR equation-based models. Values are presented as estimate (95% confidence interval).

| **Dataset** | **Age**  **group** | **EKFC** | | **CKD-EPI 2021** | | **CKD-EPI 2009** | |
| --- | --- | --- | --- | --- | --- | --- | --- |
|  |  | **Intercept** | **Slope** | **Intercept** | **Slope** | **Intercept** | **Slope** |
| **Internal** | 50-54 | 0.0484 (-0.135;0.227) | 0.789 (0.535;1.05) | 0.177 (-0.0302;0.377) | 0.796 (0.561;1.04) | 0.0872 (-0.108;0.276) | 0.849 (0.551;1.16) |
|  | 55-59 | -0.0611 (-0.262;0.135) | 0.491 (0.248;0.742) | -0.149 (-0.387;0.0787) | 0.195 (0.0320;0.397) | -0.0810 (-0.300;0.130) | 0.551 (0.239;0.867) |
|  | 60-64 | -0.0718 (-0.241;0.0939) | 0.750 (0.553;0.957) | -0.0374 (-0.223;0.144) | 0.745 (0.558;0.939) | 0.00209 (-0.175;0.175) | 1.01 (0.768;1.26) |
|  | 65-69 | 0.0752 (-0.100;0.248) | 0.882 (0.665;1.11) | 0.00830 (-0.188;0.200) | 0.708 (0.508;0.916) | 0.124 (-0.0584;0.303) | 0.928 (0.688;1.18) |
|  | 70-74 | -0.00720 (-0.184;0.167) | 0.673 (0.448;0.908) | 0.0908 (-0.100;0.277) | 0.547 (0.339;0.761) | 0.0131 (-0.172;0.194) | 0.703 (0.459;0.954) |
|  | 75-79 | -0.0539 (-0.278;0.169) | 0.520 (0.254;0.812) | -0.0928 (-0.335;0.143) | 0.573 (0.287;0.870) | -0.0607 (-0.294;0.168) | 0.719 (0.409;1.04) |
|  | 80-84 | -0.0582 (-0.262;0.145) | 0.783 (0.488;1.11) | -0.0715 (-0.288;0.141) | 0.646 (0.377;0.927) | -0.0325 (-0.243;0.175) | 0.687 (0.413;0.975) |
|  | 85-89 | 0.0707 (-0.363;0.507) | 0.339 (-0.197;0.980) | -0.0688 (-0.530;0.377) | 0.0744 (-0.124;0.322) | 0.131 (-0.310;0.565) | -0.0374 (-0.422;0.245) |
|  | 90+ | 0.625 (-0.449;1.72) | 2.52 (0.438;5.59) | 0.542 (-0.581;1.60) | 7.04 (2.07;17.7) | 0.371 (-0.752;1.43) | 13.1 (3.55; NA) |
|  | **Overall** | -0.00990 (-0.0795;0.0593) | 0.754 (0.673;0.838) | -0.00162 (-0.0783;0.0743) | 0.621 (0.543;0.700) | 0.0201 (-0.0528;0.0925) | 0.800 (0.709;0.892) |
| **External** | 50-54 | -0.560 (-0.772;-0.358) | 0.720 (0.410;1.04) | -0.185 (-0.414;0.0317) | 0.540 (0.247;0.872) | -0.533 (-0.749;-0.328) | 1.23 (0.891;1.59) |
|  | 55-59 | -0.288 (-0.477;-0.106) | 0.891 (0.600;1.20) | -0.102 (-0.309;0.0953) | 0.910 (0.606;1.22) | -0.338 (-0.535;-0.149) | 1.08 (0.774;1.40) |
|  | 60-64 | -0.445 (-0.621;-0.275) | 0.978 (0.705;1.26) | -0.353 (-0.550;-0.165) | 1.14 (0.859;1.42) | -0.491 (-0.677;-0.312) | 0.968 (0.702;1.24) |
|  | 65-69 | -0.367 (-0.530;-0.209) | 1.00 (0.738;1.28) | -0.140 (-0.314;0.0273) | 1.18 (0.916;1.45) | -0.329 (-0.497;-0.167) | 1.12 (0.847;1.40) |
|  | 70-74 | -0.380 (-0.554;-0.211) | 1.22 (0.908;1.54) | -0.208 (-0.395;-0.0279) | 1.10 (0.815;1.39) | -0.384 (-0.567;-0.207) | 1.13 (0.823;1.45) |
|  | 75-79 | -0.564 (-0.741;-0.391) | 1.17 (0.836;1.52) | -0.429 (-0.623;-0.242) | 1.21 (0.886;1.56) | -0.515 (-0.702;-0.334) | 1.15 (0.839;1.49) |
|  | 80-84 | -0.506 (-0.695;-0.322) | 0.950 (0.594;1.33) | -0.455 (-0.660;-0.257) | 0.864 (0.514;1.23) | -0.482 (-0.682;-0.288) | 0.922 (0.583;1.27) |
|  | **Overall** | -0.441 (-0.509;-0.374) | 0.957 (0.865;1.05) | -0.270 (-0.344;-0.198) | 0.940 (0.847;1.03) | -0.434 (-0.505;-0.364) | 1.04 (0.946;1.15) |

Estimates for the 85–89 and 90+ strata in the internal dataset should be interpreted with caution due to small sample sizes. In the 90+ stratum, the upper confidence interval for the CKD-EPI 2009 calibration slope could not be estimated because the sparse data prevented the profile likelihood from converging to an upper bound. No external estimates are available for these age groups as KNHANES participants were aged up to 80 years. Abbreviations: EKFC, European Kidney Function Consortium; CKD-EPI, Chronic Kidney Disease Epidemiology Collaboration; NHANES, National Health and Nutrition Examination Survey; KNHANES, Korea National Health and Nutrition Examination Survey
