# Supplementary material for: MERWACS: Development and external validation of a non-invasive machine learning tool for identifying subjects to be screened for CKD
Source: PLOS Digit Health. 2026 Jul 9;5(7):e0001486. doi: 10.1371/journal.pdig.0001486 (PMC13349138; doi:10.1371/journal.pdig.0001486)
Supplement: Peer review history

# Peer review: Decision Letter 0

**DOI:** 10.1371/journal.pdig.0001486.r001

**Authors:** Yuzhe Yang

9 Mar 2026

PDIG-D-25-00923

MERWACS: Development and External Validation of a Non-Invasive Machine Learning Tool for Identifying Subject to be Screened for CKD

PLOS Digital Health

Dear Dr. Yoo,

Thank you for submitting your manuscript to PLOS Digital Health. After careful consideration, we feel that it has merit but does not fully meet PLOS Digital Health's publication criteria as it currently stands. Therefore, we invite you to submit a revised version of the manuscript that addresses the points raised during the review process.

Please submit your revised manuscript by May 08 2026 11:59PM. If you will need more time than this to complete your revisions, please reply to this message or contact the journal office at digitalhealth@plos.org. When you're ready to submit your revision, log on to https://www.editorialmanager.com/pdig/ and select the 'Submissions Needing Revision' folder to locate your manuscript file.

Please include the following items when submitting your revised manuscript:

* A letter that responds to each point raised by the editor and reviewer(s). You should upload this letter as a separate file labeled 'Response to Reviewers'. This file does not need to include responses to any formatting updates and technical items listed in the 'Journal Requirements' section below.

* A marked-up copy of your manuscript that highlights changes made to the original version. You should upload this as a separate file labeled 'Revised Manuscript with Track Changes'.

* An unmarked version of your revised paper without tracked changes. You should upload this as a separate file labeled 'Manuscript'.

If you would like to make changes to your financial disclosure, competing interests statement, or data availability statement, please make these updates within the submission form at the time of resubmission. Guidelines for resubmitting your figure files are available below the reviewer comments at the end of this letter.

We look forward to receiving your revised manuscript.

Kind regards,

Wisit Cheungpasitporn

Academic Editor

PLOS Digital Health

Yuzhe Yang

Section Editor

PLOS Digital Health

Leo Anthony Celi

Editor-in-Chief

PLOS Digital Health

orcid.org/0000-0001-6712-6626



**Journal Requirements:**

1. Please send a completed 'Competing Interests' statement, including any COIs declared by your co-authors. If you have no competing interests to declare, please state "The authors have declared that no competing interests exist". Otherwise please declare all competing interests beginning with the statement "I have read the journal's policy and the authors of this manuscript have the following competing interests:"

2. Please ensure that your Ethics Statement is available in its entirety at the beginning of your Methods section, under a subheading 'Ethics Statement'.

3. Please upload separate figure files in .tif or .eps format. Also, remove the figures from your manuscript file but keep the legends

For more information about figure files please see our guidelines:

https://journals.plos.org/digitalhealth/s/figures

https://journals.plos.org/digitalhealth/s/figures#loc-file-requirements

4. We have noticed that you have uploaded Supporting Information files, but you have not included a list of legends. Please add a full list of legends for your Supporting Information files after the references list.

If the reviewer comments include a recommendation to cite specific previously published works, please review and evaluate these publications to determine whether they are relevant and should be cited. There is no requirement to cite these works unless the editor has indicated otherwise.

**Additional Editor Comments (if provided):**

[Note: HTML markup is below. Please do not edit.]

**Reviewers' Comments:**

Reviewer's Responses to Questions

**Comments to the Author**

1. Does this manuscript meet PLOS Digital Health’s publication criteria? Is the manuscript technically sound, and do the data support the conclusions? The manuscript must describe methodologically and ethically rigorous research with conclusions that are appropriately drawn based on the data presented.

Reviewer #1: Yes

Reviewer #2: Yes

**********

2. Has the statistical analysis been performed appropriately and rigorously?

Reviewer #1: Yes

Reviewer #2: Yes

**********

3. Have the authors made all data underlying the findings in their manuscript fully available (please refer to the Data Availability Statement at the start of the manuscript PDF file)?

The PLOS Data policy requires authors to make all data underlying the findings described in their manuscript fully available without restriction, with rare exception. The data should be provided as part of the manuscript or its supporting information, or deposited to a public repository. For example, in addition to summary statistics, the data points behind means, medians and variance measures should be available. If there are restrictions on publicly sharing data—e.g. participant privacy or use of data from a third party—those must be specified.

Reviewer #1: Yes

Reviewer #2: Yes

**********

4. Is the manuscript presented in an intelligible fashion and written in standard English?

PLOS Digital Health does not copyedit accepted manuscripts, so the language in submitted articles must be clear, correct, and unambiguous. Any typographical or grammatical errors should be corrected at revision, so please note any specific errors here.

Reviewer #1: Yes

Reviewer #2: Yes

**********

5. Review Comments to the Author

Please use the space provided to explain your answers to the questions above. You may also include additional comments for the author, including concerns about dual publication, research ethics, or publication ethics. (Please upload your review as an attachment if it exceeds 20,000 characters)

**Reviewer #1** : This manuscript presents the development and external validation of MERWACS, a non-invasive machine-learning–based screening tool for identifying adults who should undergo laboratory testing for chronic kidney disease (CKD). The study is carefully executed, methodologically rigorous, and transparently reported, with notable strengths including use of long-term NHANES data, external validation in a geographically distinct population (KNHANES), and strong emphasis on interpretability and usability via an open-access application. The work addresses an important public health gap: scalable, accessible pre-screening for CKD in the general adult population. However, several conceptual and methodological issues require clarification or strengthening to ensure appropriate interpretation, generalizability, and positioning of the tool within clinical and public health practice.

Major Issues

1. Conceptual framing of the outcome and its implications. The composite outcome, albuminuria (uACR ≥30 mg/g) or eGFR below the age- and sex-specific 2.5th percentile, is innovative and biologically motivated. However, this definition likely results in very high prevalence estimates (e.g., >30% using EKFC), which diverge substantially from conventional CKD prevalence estimates. While the authors acknowledge this, the implications are not fully explored. Specifically: how should users such as clinicians, patients, policymakers interpret a predicted probability of “reduced kidney health” when the outcome includes both pathological disease and early deviation from healthy reference norms?, does the tool risk over-identification and potential overuse of downstream testing, particularly in older adults? and, how does this definition align or intentionally not align with KDIGO staging and guideline based decision thresholds? A clearer articulation of how MERWACS complements (rather than conflicts with) existing CKD definitions and screening frameworks is needed.

2. Clinical and public health utility relative to modest discrimination. The reported discrimination (ROCAUC ~0.68–0.73) is consistently moderate. While the authors correctly frame this as an intentional trade-off for accessibility, the manuscript would benefit from a more explicit discussion of decision utility. Authors should discuss on the use-case scenarios, how MERWACS would change practice beyond existing approaches.

3. Transportability and external validity beyond KNHANES. This is a major strength, however several limitations exist and poorly addressed. These are, 1) the outcome definition relies on age- and sex-specific eGFR percentiles derived from European reference cohorts, which may not be appropriate for Asian, African, or other populations; 2) Ethnicity is treated differently across datasets, raising concerns about stability of predictor effects.

4. Cross-sectional design and inability to predict incident disease. I think MERWACS predicts prevalent reduced kidney function, not incident CKD. This must be fixed in entire manuscript.

5. Discussion would benefit from a more explicit treatment of implementation science considerations like safeguards, communication strategies, and integration with healthcare systems.

Minor issues

• Model comparison interpretation. Differences between XGBoost and logistic regression are modest. The rationale for preferring ML over simpler models should be more carefully framed around interpretability and flexibility rather than performance alone.

• Exclusion of dietary variables is reasonable, but a brief justification of why other non-invasive variables (e.g., physical activity) were not retained would improve transparency.

• Calibration plots are informative, but numerical calibration metrics stratified by age group would further strengthen confidence, given the age-centric outcome definition.

• Editorial issues (e.g., “Subject” vs. “Subjects” in the title) should be corrected.

• Some figures are dense; improving labeling

**Reviewer #2** : 1.The use of European-derived age- and sex-specific eGFR percentiles as the outcome reference for U.S. and Korean populations may introduce systematic misclassification. I suggest justifying the transferability of European eGFR percentiles, because applying these norms to U.S. and Korean populations may bias outcome classification across ethnic groups.

2. The very high prevalence of the composite outcome (up to ~35%) risks blurring the distinction between “screen-positive” and “clinically meaningful disease.”

I recommend quantifying potential misclassification, since the 2.5th percentile threshold leads to unusually high prevalence and may label physiologic aging as “reduced kidney function.”

3. The external validation relies partly on imputed predictors, which weakens claims of real-world deployability. Suggest expanding discussion on external imputation, as imputing unavailable KNHANES predictors could inflate external validation performance.

4. The incremental value over simpler logistic regression is statistically modest. please clarify the clinical meaning of a positive MERWACS result, because “screen-positive” does not clearly map to guideline-based CKD diagnosis.

5. need to report decision-curve analysis

6. comparing performance to age-only or hypertension-only models

7. propose adding calibration-in-the-large metrics

**********

6. PLOS authors have the option to publish the peer review history of their article (what does this mean?). If published, this will include your full peer review and any attached files.

**Do you want your identity to be public for this peer review?**  If you choose “no”, your identity will remain anonymous but your review may still be made public.

For information about this choice, including consent withdrawal, please see our Privacy Policy.

Reviewer #1: No

Reviewer #2: No

**********

[NOTE: If reviewer comments were submitted as an attachment file, they will be attached to this email and accessible via the submission site. Please log into your account, locate the manuscript record, and check for the action link "View Attachments". If this link does not appear, there are no attachment files.]

**Figure resubmission:**

While revising your submission, we strongly recommend that you use PLOS’s NAAS tool (https://ngplosjournals.pagemajik.ai/artanalysis) to test your figure files. NAAS can convert your figure files to the TIFF file type and meet basic requirements (such as print size, resolution), or provide you with a report on issues that do not meet our requirements and that NAAS cannot fix.

After uploading your figures to PLOS’s NAAS tool - https://ngplosjournals.pagemajik.ai/artanalysis, NAAS will process the files provided and display the results in the "Uploaded Files" section of the page as the processing is complete. If the uploaded figures meet our requirements (or NAAS is able to fix the files to meet our requirements), the figure will be marked as "fixed" above. If NAAS is unable to fix the files, a red "failed" label will appear above. When NAAS has confirmed that the figure files meet our requirements, please download the file via the download option, and include these NAAS processed figure files when submitting your revised manuscript.

**Reproducibility:**

To enhance the reproducibility of your results, we recommend that authors of applicable studies deposit laboratory protocols in protocols.io, where a protocol can be assigned its own identifier (DOI) such that it can be cited independently in the future. Additionally, PLOS ONE offers an option to publish peer-reviewed clinical study protocols. Read more information on sharing protocols at https://plos.org/protocols?utm_medium=editorial-email&utm_source=authorletters&utm_campaign=protocols

# Author comment: Author response to Decision Letter 1

**DOI:** 10.1371/journal.pdig.0001486.r002

25 Mar 2026

Attachment Submitted filename: reviewers_response_1st_revision.docx

# Peer review: Decision Letter 1

**DOI:** 10.1371/journal.pdig.0001486.r003

**Authors:** Mathew Kiang

22 May 2026

MERWACS: Development and External Validation of a Non-Invasive Machine Learning Tool for Identifying Subject to be Screened for CKD

PDIG-D-25-00923R1

Dear Dr. Yoo,

We are pleased to inform you that your manuscript 'MERWACS: Development and External Validation of a Non-Invasive Machine Learning Tool for Identifying Subject to be Screened for CKD' has been provisionally accepted for publication in PLOS Digital Health.

Before your manuscript can be formally accepted you will need to complete some formatting changes, which you will receive in a follow-up email from a member of our team.

Please note that your manuscript will not be scheduled for publication until you have made the required changes, so a swift response is appreciated.

IMPORTANT: The editorial review process is now complete. PLOS will only permit corrections to spelling, formatting or significant scientific errors from this point onwards. Requests for major changes, or any which affect the scientific understanding of your work, will cause delays to the publication date of your manuscript.

If your institution or institutions have a press office, please notify them about your upcoming paper to help maximize its impact. If they'll be preparing press materials, please inform our press team as soon as possible -- no later than 48 hours after receiving the formal acceptance. Your manuscript will remain under strict press embargo until 2 pm Eastern Time on the date of publication. For more information, please contact digitalhealth@plos.org.

Thank you again for supporting Open Access publishing; we are looking forward to publishing your work in PLOS Digital Health.

Best regards,

Wisit Cheungpasitporn, MD

Academic Editor

PLOS Digital Health

***********************************************************

**Additional Editor Comments (if provided):**

**Reviewer Comments (if any, and for reference):**

Reviewer's Responses to Questions

**Comments to the Author**

1. If the authors have adequately addressed your comments raised in a previous round of review and you feel that this manuscript is now acceptable for publication, you may indicate that here to bypass the “Comments to the Author” section, enter your conflict of interest statement in the “Confidential to Editor” section, and submit your "Accept" recommendation.

Reviewer #2: All comments have been addressed

**********

2. Does this manuscript meet PLOS Digital Health’s publication criteria? Is the manuscript technically sound, and do the data support the conclusions? The manuscript must describe methodologically and ethically rigorous research with conclusions that are appropriately drawn based on the data presented.

Reviewer #2: Yes

**********

3. Has the statistical analysis been performed appropriately and rigorously?

Reviewer #2: Yes

**********

4. Have the authors made all data underlying the findings in their manuscript fully available (please refer to the Data Availability Statement at the start of the manuscript PDF file)?

The PLOS Data policy requires authors to make all data underlying the findings described in their manuscript fully available without restriction, with rare exception. The data should be provided as part of the manuscript or its supporting information, or deposited to a public repository. For example, in addition to summary statistics, the data points behind means, medians and variance measures should be available. If there are restrictions on publicly sharing data—e.g. participant privacy or use of data from a third party—those must be specified.

Reviewer #2: Yes

**********

5. Is the manuscript presented in an intelligible fashion and written in standard English?

PLOS Digital Health does not copyedit accepted manuscripts, so the language in submitted articles must be clear, correct, and unambiguous. Any typographical or grammatical errors should be corrected at revision, so please note any specific errors here.

Reviewer #2: Yes

**********

6. Review Comments to the Author

Please use the space provided to explain your answers to the questions above. You may also include additional comments for the author, including concerns about dual publication, research ethics, or publication ethics. (Please upload your review as an attachment if it exceeds 20,000 characters)

Reviewer #2: (No Response)

**********

7. PLOS authors have the option to publish the peer review history of their article (what does this mean?). If published, this will include your full peer review and any attached files.

**Do you want your identity to be public for this peer review?**  If you choose “no”, your identity will remain anonymous but your review may still be made public.

For information about this choice, including consent withdrawal, please see our Privacy Policy.

Reviewer #2: No

**********
